# Systems metabolic engineering of *Corynebacterium glutamicum* for efficient production of L-isoleucine

**DOI:** 10.1186/s40104-026-01459-y

**Published:** 2026-07-08

**Authors:** Junkun Cao, Ming Huang, Qi Sheng, Landong Zhang, Gang Meng, Chunguang Zhao, Aiying Wei, Jia Liu, Liming Liu

**Affiliations:** 1 School of Food Science and Engineering, Anhui Science and Technology University, Chuzhou, Anhui 239000 China; 2 School of Biotechnology and Key Laboratory of Industrial Biotechnology of Ministry of Education, Jiangnan University, Wuxi, Jiangsu 214122 China; 3 School of Life Sciences and Health Engineering, Jiangnan University, Wuxi, Jiangsu 214122 China; 4 Ningxia Eppen Biotech Co., Ltd., Yinchuan, Ningxia 750000 China

**Keywords:** Atmospheric and room-temperature plasma (ARTP) mutagenesis, *Corynebacterium glutamicum*, Fed-batch fermentation, L-Isoleucine, Modular metabolic engineering

## Abstract

**Background:**

L-Isoleucine is an essential branched-chain amino acid for livestock and poultry, supporting protein accretion and regulating energy metabolism, immune function, and stress resilience.

**Results:**

A genetically stable L-isoleucine producer, *Corynebacterium glutamicum* cgl-Ile0, was obtained via biosensor-assisted ARTP mutagenesis, and produced 11.52 g/L L-isoleucine in a 5-L fermenter, with a yield of 0.11 g/g and a productivity of 0.24 g/L/h. Whole-genome resequencing revealed four mutations associated with the phenotype, including *aspB*^V346I^, *asd*^P27E^, *brnE*^L87S^, and *brnF*^R28P^. Structure-guided protein engineering of two rate-limiting enzymes—threonine dehydratase (TD) and acetohydroxyacid synthase (AHAS)—generated strain cgl-Ile1, increasing titer, yield, and productivity by 39.50%, 27.27%, and 37.50%, respectively, relative to strain cgl-Ile0. Subsequent modular optimization of L-isoleucine biosynthesis, oxaloacetate supply module, cofactor-supply module, and transport/export module yielded the final strain cgl-Ile8. Through optimization pH and dissolved oxygen, the titer, yield and productivity of L-isoleucine produced by strain cgl-Ile8 in a 5-L fermenter were 48.49 g/L, 0.31 g/g and 1.01 g/L/h, which were 4.21-, 2.82-, and 4.21-fold those of strain cgl-Ile0, respectively. Scale-up to a 50-L fermenter further increased the titer, yield and productivity to 50.12 g/L, 0.32 g/g and 1.04 g/L/h, respectively, representing the highest reported L-isoleucine titer in *C. glutamicum* to date.

**Conclusions:**

We developed an industrial L-isoleucine-producing *C. glutamicum* strain by integrating biosensor-guided ARTP mutagenesis, structure-guided protein engineering, and modular pathway rewiring, providing a practical and transferable framework for constructing GRAS amino-acid producers for animal nutrition.

**Supplementary Information:**

The online version contains supplementary material available at 10.1186/s40104-026-01459-y.

## Introduction

As an essential branched-chain amino acid for livestock and poultry, L-isoleucine is required not only as a direct substrate for protein biosynthesis but also as a key contributor to energy metabolism, immune modulation, and stress resilience [1, 2]. With the increasing application of low-protein feed strategies, the importance of L-isoleucine in modern feed formulation has been further reinforced. In line with this trend, global demand continues to rise: the market was valued at USD 2.32 billion in 2023 and is projected to reach USD 3.03 billion by 2028, corresponding to a compound annual growth rate (CAGR) of 4.46%. Therefore, the development of efficient and sustainable industrial production processes is important to ensure the reliable supply of L-isoleucine [3].

Microbial fermentation still is the predominant industrial route for L-isoleucine production, with *Escherichia coli* and *C. glutamicum* serving as the principal industrial strain [4, 5]. *E. coli* has been preferred owing to its well-characterized genetic background, facile genetic manipulation, rapid growth, and low nutritional requirements [4]. Therefore, it has been extensively engineered for the biosynthesis of a serial of amino acids, such as L-arginine [6], L-alanine [7], L-valine [8], L-leucine [9], L-threonine [10], L-tryptophan [11], L-phenylalanine [12], and L-tyrosine [13]. By combining metabolic rewiring with protein engineering, L-isoleucine-producing capacity of *E. coli* has been substantially enhanced [14]. Compared with *E. coli*, *C. glutamicum* offers advantages that are particularly relevant to large-scale amino-acid fermentation, including high biosafety and robustness (generally recognized as safe, GRAS status), strong stress tolerance and environmental adaptability [15]. However, the reported L-isoleucine titers in *C. glutamicum* have generally remained below industrial requirements [16, 17], largely due to the complexity of its regulatory architecture and the limited implementation of coordinated, system-level engineering strategies.

The L-isoleucine biosynthetic capacity of *C. glutamicum* is primarily constrained by three factors [18]. First, the pathway is metabolically complex, involving an extended reaction sequence and multiple regulatory checkpoints. Starting from oxaloacetate, L-isoleucine formation proceeds through 11 enzymatic reactions, and carbon flux is strongly restricted by at least five major branch points, including the pyruvate–oxaloacetate node (oxaloacetate supply), the α-ketoglutarate node (glutamate supply), the aspartate semialdehyde node (competition with L-lysine biosynthesis), the homoserine node (competition with L-methionine biosynthesis), and the glucose-6-phosphate node (NADPH supply) [19, 20]. Second, pathway enzymes are tightly controlled by feedback inhibition: threonine dehydratase (TD; encoded by *ilvA*) and acetohydroxyacid synthase (AHAS; encoded by *ilvBN*) are inhibited by L-isoleucine, whereas aspartokinase (LysC), homoserine dehydrogenase (Hom), and homoserine kinase (ThrB) are inhibited by the precursor L-threonine [21]. Third, L-isoleucine biosynthesis requiring four mole NADPH and one mole ATP when produce a mole L-isoleucine [22, 23]. Collectively, pathway complexity, stringent regulation, and higher redox requirements complicate rational, systems-level engineering of *C. glutamicum* for enhanced L-isoleucine production [24].

In this study, we addressed these intrinsic limitations by developing an integrated systematic metabolic engineering strategy to improve L-isoleucine biosynthesis in *C. glutamicum*. In this study, irrational and rational metabolic engineering strategies were combined to generate isoleucine producing strains by combining biosensor-assisted high-throughput screening (HTS) with genome-wide analysis, protein engineering to relieve the inhibition of pathway enzymes by end products, and modular optimization of precursor supply and cofactor metabolism. Unlike conventional studies that rely on empirical trials or single-layer interventions, this study focused on the precise identification and targeted reprogramming of key enzymes, metabolic mechanisms, and regulatory constraints for branched-chain amino acid biosynthesis. Taken together, this study is expected to provide mechanistic insights into the regulation of L-isoleucine metabolism and to establish a general paradigm for amino acid production relevant to animal nutrition and biotechnology.

## Materials and methods

### Strains, plasmids and primers

All strains, plasmids, and primers used in this study are listed in Tables S1 and S8. The genomic integration sites, heterologous genes, genetic engineering targets, and promoter sets used for pathway construction are summarized in Supplementary Tables S2–S7. *C. glutamicum* ATCC 13032 was subjected to random mutagenesis to generate production candidates, from which an L-isoleucine–producing chassis strain was selected. *E. coli* DH5α was used for routine plasmid propagation and vector construction. The suicide vector pK18mob*sacB* was employed for chromosomal gene editing*.*

### Genome manipulation

Genome editing in *C. glutamicum* was performed using a pK18mob*sacB*-derived suicide vector carrying the mutated or target fragment flanked by two homologous arms (~1,000 bp each). For chromosomal modification, 1 μg of the corresponding plasmid (e.g., pK18-*thrABC*) was introduced into *C. glutamicum* by electroporation, and mutants were obtained via two-step homologous recombination. Briefly, transformants were selected on LBGB agar supplemented with kanamycin (25 μg/mL) to enrich single-crossover integrants. Recombinants were then cultured in LBGB medium without kanamycin for 4 h, serially diluted, and plated on LBGB agar containing 10% (w/v) sucrose to isolate double-crossover mutants. Candidate colonies were confirmed by colony PCR and Sanger sequencing.

Construction of the *Ec*TD(P441F) mutant expression strain. The plasmid and mutagenic primer sequences are provided in Tables S1 and S8, respectively. The *Ec*TD(P441F) mutant was generated by whole-plasmid PCR-based site-directed mutagenesis. Briefly, PCR amplification was carried out using the recombinant plasmid as the template. The amplified product was purified and digested with DpnI for 30 min to remove the methylated parental plasmid DNA. The digested product was then transformed into chemically competent *E. coli* BL21 (DE3) cells by chemical transformation, and the transformants were cultured for subsequent experiments [25].

### The construction and characterization of biosensors

The *lrp* gene and the promoter *brnEF*^N7^ were amplified from the genomic DNA of *C. glutamicum* ATCC 13032 by PCR using DNA polymerase and the corresponding primer pairs listed in Table S7. The reporter gene *gfp* was obtained by gene synthesis. The *lrp*, P_*brnEF*_^N7^, and *gfp* fragments were individually assembled into the plasmid backbones pECXK99E, pXMJ19, and pZK001 by homologous recombination using AB cloning enzymes according to the manufacturer’s instructions, generating the biosensor plasmids pECXK99E-Sensor^Ile^, pXMJ19-Sensor^Ile^, and pZK001-Sensor^Ile^, respectively [14, 26].

All three constructs contained the same sensing module consisting of *lrp*, P_*brnEF*_^N7^, and *gfp*, whereas they differed in plasmid backbone. Specifically, pECXK99E provided a relatively simple expression background commonly used for single-gene expression in *C. glutamicum*, pXMJ19 provided a shuttle-vector background suitable for relatively stronger and more flexible gene expression, and pZK001 was a laboratory-constructed plasmid used as an alternative backbone for comparison. Therefore, the three biosensor constructs were expected to exhibit different sensing performances due to differences in vector context.

The recombinant plasmids were verified by colony PCR and Sanger sequencing before being used for subsequent biosensor evaluation. To characterize the biosensors, strains harboring the different constructs were cultivated under identical experimental conditions, and their fluorescence responses were measured over a range of L-isoleucine concentrations. Biosensor performance was compared based on response intensity and response range. Among the three constructs, pECXK99E-SensorIle showed the best sensing performance and was therefore selected for subsequent screening.

### Atmospheric and room temperature plasma (ARTP) mutagenesis

An automated atmospheric and room temperature plasma (ARTP) mutagenesis system (Siqingyuan Biotechnology Co., Ltd., Wuxi, China) was used to randomly mutagenize wild-type *C. glutamicum* ATCC 13032 as previously described [27]. Helium was used as the working gas to generate the plasma jet. The nozzle-to-sample distance was maintained at 2 mm, the helium flow rate was set to 10 L/min, and the radiofrequency power was fixed at 100 W [28]. To determine the optimal exposure duration, the lethality of *C. glutamicum* ATCC 13032 was evaluated across a range of treatment times [29]. Briefly, 10 μL of a freshly prepared exponential-phase cell suspension was evenly spread onto a sterile metal plate and exposed to the ARTP jet for 80, 100, 120, 140, or 160 s. After treatment, cells were immediately resuspended by transferring the metal plate into a sterile microcentrifuge tube containing 1 mL of sterile distilled water. The suspension was serially diluted, plated on solid medium, and incubated at 30 °C for 48 h. The lethality rate was calculated:

$$\text{Lethality rate}~(\%) = [(\mathrm{C} - \mathrm{S}) / \mathrm{C}] \times 100,$$

where C is the mean colony count of untreated controls and S is the mean colony count of ARTP-treated samples. Colony counts were determined in duplicate using a colony-forming unit (CFU) assay.

### Mutation strain genetic stability test screening

The detailed operation process for evaluating genetic stability at 15 generations is as follows: Seed culture: The strain cgl-Ile0 was activated on LBGB solid medium at 30 °C for 24 h, and a single colony was picked and inoculated into 50 mL of LBGB liquid medium, cultured at 30 °C, 220 r/min for 12 h to obtain seed liquid. Continuous passage: 1 mL of seed liquid was inoculated into 50 mL of fresh LBGB liquid medium (inoculation amount 2%, v/v), cultured under the same conditions until the logarithmic growth phase (OD_600_ ≈ 1.0–1.2), which was defined as one generation. This operation was repeated continuously for 15 generations. Stability detection: After the 1^st^, 5^th^, 10^th^, and 15^th^ generations, the strains were respectively inoculated into the L-isoleucine fermentation medium, cultured at 30 °C, 220 r/min for 36 h. The L-isoleucine yield and cell growth of each generation were measured to verify the genetic stability of the mutant strains [30, 31].

### Whole-genome sequencing and data analyses

Whole-genome resequencing was performed for mutant strain cgl-Ile0 to identify mutation sites. Genomic DNA was extracted using the cetyltrimethylammonium bromide (CTAB) method, and DNA quality was assessed by 1% agarose gel electrophoresis. Illumina paired-end (PE) library construction and sequencing were conducted by Suzhou Genewiz Biotechnology Co., Ltd.

For bioinformatic analysis, raw reads were trimmed and quality-filtered using fastp to remove low-quality sequences [32]. Clean reads were aligned to the reference genome of the parental *C. glutamicum* ATCC 13032 using the BWA-MEM algorith. PCR duplicates were removed using Picard MarkDuplicates [33]. Sequencing depth and genome coverage were calculated using SAMtools based on the resulting BAM files. Variants were identified using Snippy followed by filtering based on sequencing depth and mapping quality [34]. The retained variants were functionally annotated using SnpEff to predict their potential genomic effects.

### Protein expression and purification

Wild-type and engineered TD and AHAS enzymes were overexpressed in *E. coli* BL21 (DE3). Cells were cultured in 30 mL LB medium containing kanamycin (50 μg/mL) at 37 °C to an OD_600_ of 0.6–0.8. Protein expression was induced by addition of isopropyl β-D-1-thiogalactopyranoside (IPTG) to a final concentration of 0.42 mmol/L, followed by incubation at 25 °C for 14 h. Cells were harvested by centrifugation at 4,629 × *g* for 10 min, washed twice, and resuspended in Buffer A (20 mmol/L sodium phosphate, 0.5 mol/L NaCl, 20 mmol/L imidazole, 1 mmol/L dithiothreitol (DTT), pH 7.4) [35]. Cell lysis was achieved by sonication, and the clarified lysate was subjected to immobilized metal affinity chromatography using a His SpinTrap column (GE Healthcare; 28-4013-53) according to the manufacturer’s instructions, except that Buffer B (20 mmol/L sodium phosphate, 0.5 mol/L NaCl, 500 mmol/L imidazole, 1 mmol/L DTT, pH 7.4) was used for elution. Purified proteins were concentrated and buffer-exchanged into Buffer C (20 mmol/L sodium phosphate, 0.15 mol/L NaCl, 0.1 mmol/L DTT, pH 7.5) using an Amicon Ultra-4 centrifugal filter unit with a 30-kDa cutoff (Ultracel-30; Millipore, USA). Protein purity was assessed by SDS–PAGE, and protein concentration was determined using a BCA Protein Assay Kit (Thermo Fisher Scientific).

### In vitro enzyme assays

TD activity was determined by quantifying α-ketobutyrate formation. Standard reactions contained 20 μmol/L pyridoxal 5′-phosphate (PLP), 50 mmol/L potassium phosphate buffer (pH 7.5), threonine (10 mmol/L unless otherwise stated; 0–100 mmol/L as indicated), and purified TD (2 μg/mL). The effect of L-isoleucine on TD activity was evaluated in the presence of 10 mmol/L threonine following previous studies. All assays were performed at 30 °C in triplicate. α-Ketobutyrate was quantified by HPLC (Waters, Milford, MA, USA) equipped with an Aminex HPX-87H column (7.8 mm × 300 mm) using 5 mmol/L H₂SO₄ as the mobile phase (flow rate, 0.6 mL/min; column temperature, 52 °C) with detection at 210 nm.

AHAS activity was measured as previously described. The 1-mL reaction mixture consisted of 100 mmol/L potassium phosphate buffer (pH 7.8) containing 100 mmol/L sodium pyruvate, 100 mmol/L 2-ketobutyrate, 10 mmol/L MgCl_2_, and 0.2 mmol/L thiamine pyrophosphate. Reactions were initiated by adding purified AHAS and incubated at 37 °C for 1 h. The reaction was quenched by adding 100 μL of 3 mol/L H_2_SO_4_, followed by incubation at 65 °C for 15 min. Subsequently, 1 mL of 0.5% (w/v) creatine and 1 mL of an α-naphthol solution freshly prepared in 2.5 mol/L NaOH were added, and the mixture was incubated at 65 °C for 20 min and then cooled to room temperature. Absorbance was measured at 525 nm (OD_525_). Standards and controls were processed in parallel under identical conditions. Feedback inhibition by L-leucine was assessed at different L-leucine concentrations. One unit of activity was defined as the amount of enzyme required to form 1 mol of 2-aceto-2-hydroxybutyrate per minute at 30 °C, and specific activity was expressed as units per gram of protein. All assays were performed in triplicate.

### Measurement of intracellular NADPH

The intracellular NADPH/NADP^+^ ratio was quantified using a commercial NADP/NADPH assay kit (BioAssay Systems, Hayward, CA, USA) according to the manufacturer’s instructions. Detailed procedures are provided in Method S1.

### Culture medium

The culture media used in this study are listed in Tables S8–S12. Luria–Bertani (LB) medium containing 5 g/L yeast extract, 10 g/L tryptone, and 10 g/L NaCl was used for plasmid construction. Prior to cultivation, the medium pH was adjusted with NH₄OH (25%, v/v). The glucose feeding solution was prepared at a final concentration of 800 g/L.

### Shaking flask fermentation

*C. glutamicum* strains were initially cultivated on agar plates at 30 °C for 24 h. Colonies from a single plate were collected and inoculated into 100 mL of seed medium in a 500-mL baffled shake flask, followed by incubation at 30 °C and 220 r/min for 36 h. During cultivation, 2-mL samples were collected every 12 h for amino acid analysis.

For bioreactor inoculum preparation, cells from four plates were inoculated in parallel into four 500-mL shake flasks, each containing 100 mL of seed medium, and cultivated at 30 °C and 220 r/min for 18 h. The resulting seed cultures (total volume, 400 mL) were pooled and transferred into a 5-L bioreactor containing 2.5 L of fermentation medium.

### Fed-batch fermentation in 5-L fermenter

Bioreactor fermentations were performed at 30 °C. The pH was automatically maintained at 7.0 by the addition of aqueous ammonia (50%, v/v). Aeration was set to 2.0 vvm, and dissolved oxygen (DO) was controlled at 30%–40%. Samples (3–5 mL) were collected every 4 h for determination of amino acid and glucose concentrations. Residual glucose was maintained at approximately 5 g/L by intermittent feeding of a concentrated glucose solution (800 g/L) as needed.

### Control of fermentation conditions

Cell density was monitored by measuring the optical density at 600 nm (OD_600_) using a UV-1800 spectrophotometer (Shimadzu, Japan). For glucose determination, culture samples were centrifuged at 15,871 × *g* for 15 min, and the supernatant was analyzed for residual glucose using an M-100 biosensor analyzer.

### HPLC L-isoleucine quantification

Concentrations of amino acids and organic acids were determined by high-performance liquid chromatography (HPLC) using pre-column o-phthaldialdehyde (OPA) derivatization and gradient elution. The mobile phases and operating conditions are provided in Method S2.

## Results

### Biosensor-assisted ARTP mutagenesis of *C. glutamicum* to screen L-isoleucine producing strains

**Fig. 1:**
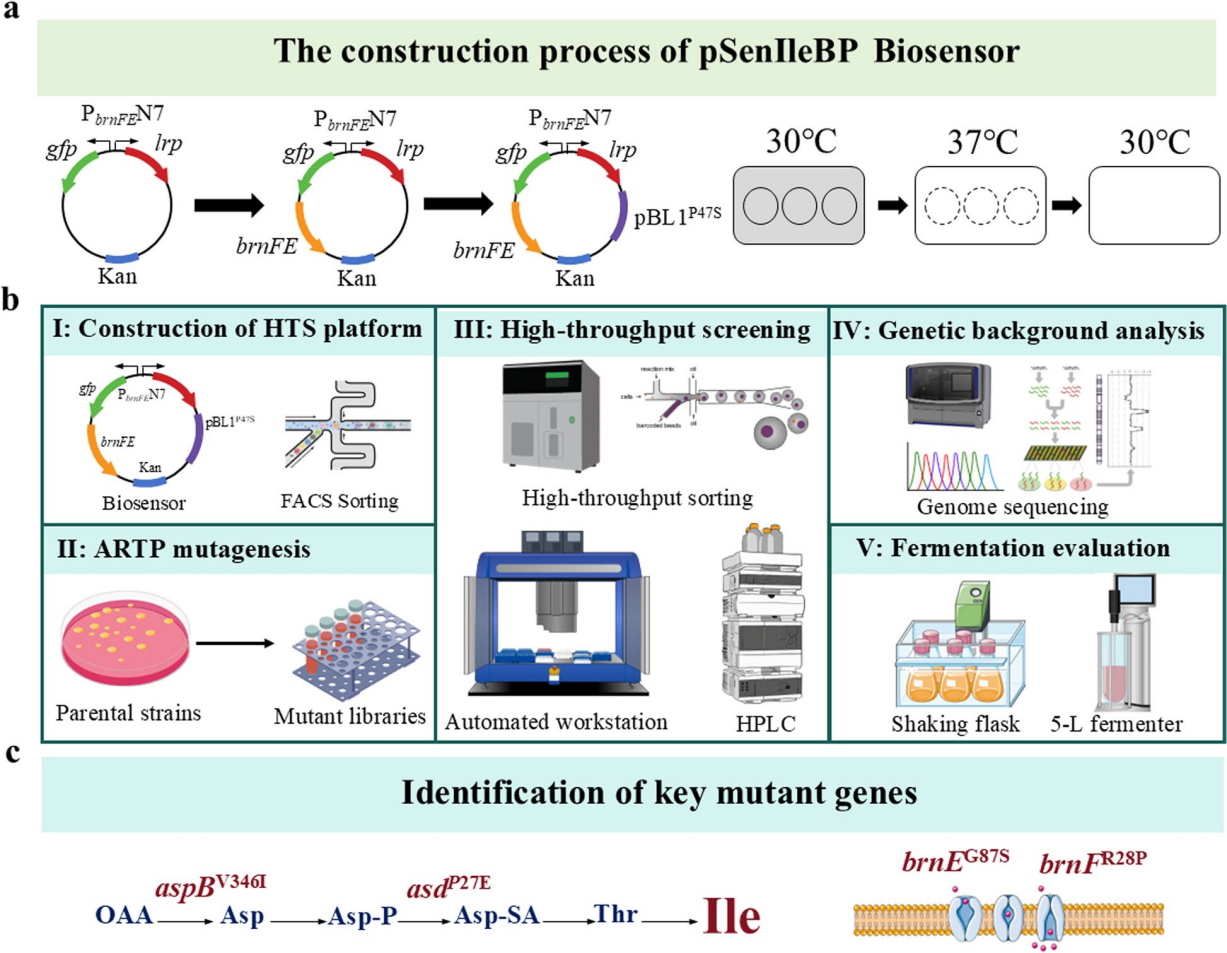
Construction of isoleucine biosensors and high-throughput screening of mutagenic strains.** a** Construction and optimization of the isoleucine biosensor pSenIleBP. **b** Screening of high-producing strains based on HTS platform. **c** Identification of key mutant genes in mutagenic strain cgl-Ile0. OAA, oxaloacetate; Asp, aspartate; Asp-P, aspartate-phosphate; Asp-SA, aspartate-semialdehyde; Thr, threonine; *aspB*^V346I^, encoding mutant of aspartate aminotransferase; *asd*^P27E^, encoding mutant of aspartate-semialdehyde dehydrogenase

To establish a HTS workflow for isolating L-isoleucine overproducers, an optimized L-isoleucine biosensor was integrated with fluorescence-activated cell sorting (FACS) (Fig. 1 and Fig. S1). The biosensor consisted of three functional components: the Lrp (an L-isoleucine-responsive TF), the Lrp-regulated promoter P_brnEF_^N7^, and the reporter gene *gfp*. These components were assembled into three plasmid backbones—pECXK99E, pXMJ19, and pZK001—yielding pECXK99E-Sensor^Ile^, pXMJ19-Sensor^Ile^, and pZK001-Sensor^Ile^, respectively. In the BT medium, the three constructs exhibited distinct dynamic response ranges to exogenous L-isoleucine (0–35 mmol/L, 0–20 mmol/L, and 0–15 mmol/L for pECXK99E-Sensor^Ile^, pXMJ19-Sensor^Ile^, and pZK001-Sensor^Ile^) (Fig. S1a–c). Since pECXK99E-Sensor^Ile^ produced the highest fluorescence output and displayed the greatest sensitivity to changes in L-isoleucine concentration, it was selected as the working biosensor and renamed pSenIle (Fig. 1a). To further broaden the sensing window, the *brnEF* operon, encoding branched-chain amino acid exporters, was linked to the reporter module, resulting in pSenIleB, which extended the dynamic response range to 0–45 mmol/L in BT medium (Fig. S1d). Finally, to enable plug-and-play deployment of the sensor, the replicon of pSenIleB was replaced with the temperature-sensitive replicon pBL1P47S, which leads to plasmid loss at 37 °C, yielding pSenIleBLP (Fig. 1a). The combination of pSenIleBLP and FACS formed the HTS platform used for screening high L-isoleucine-producing strains (Fig. 1b).

To obtain *C. glutamicum* strains capable of over-accumulating L-isoleucine, ARTP mutagenesis was applied to *C. glutamicum* ATCC 13032, followed by a dual-stage selection that integrated the FACS-based HTS platform and structural analog screening (Fig. 1 and Figs. S2–S6). An irradiation time of 140 s was used in both rounds of ARTP mutagenesis, giving a lethality rate of 98.07% (Fig. S2). After the first round, the top 1% fluorescent population was sorted by FACS and recovered by cultivation. The recovered cells were then subjected to a second round of ARTP mutagenesis, after which the top 1% fluorescent cells were again collected by FACS, plated on α-aminobutyric acid-containing medium, and further evaluated in shake flasks. Through this iterative enrichment and screening procedure, 45 colonies were isolated, and several candidate mutants with improved L-isoleucine production were obtained. (Fig. 1b and Fig. S3). Deep-well plate fermentation identified three candidates (cgl-11, cgl-22, and cgl-35) producing 1.94–2.07 g/L L-isoleucine (Fig. S4). After 15 generations, strain cgl-22 and cgl-35 showed significant production decay, with titers decreasing to 1.16 and 0.95 g/L (reductions of 40.21% and 54.11%, respectively), whereas cgl-11 maintained stable production at 1.82 g/L (Fig. S5). cgl-11 produced 4.11 g/L L-isoleucine in shake flasks (36 h) (Fig. S6a) and achieved 11.52 g/L in a 5-L fermenter (48 h), with a yield of 0.11 g/g and a productivity of 0.24 g/L/h. Thus, strain cgl-11 was selected as the genetically stable starting strain and renamed cgl-Ile0 (Fig. S6b).

To elucidate the genetic basis underlying the improved phenotype of strain cgl-Ile0, comparative genomic analysis was performed against the parental strain *C. glutamicum* ATCC 13032 (Fig. 1 and Fig. S7). The genome of strain cgl-Ile0 comprised of a single circular chromosome of 3,349,026 bp with a GC content of 53.93%, which was comparable to that of *C. glutamicum* ATCC 13032 (53.81%) (Fig. S7). Gene Ontology enrichment analysis and KEGG pathway mapping were performed on the mutated genes identified in cgl-Ile0 rather than on the entire genome (Figs. S8 and S9). Gene Ontology (GO) enrichment analysis using the Goplot R package (FDR < 0.05) indicated that the mutated genes were predominantly associated with catalytic activity and metabolic processes, which is consistent with a potential role in L-isoleucine accumulation (Fig. S9). KEGG pathway mapping further assigned these genes to 39 pathways, with major enrichment in global metabolic regulation, carbohydrate metabolism, and amino acid metabolism. Notably, mutations linked to cofactor/vitamin metabolism, energy metabolism, and membrane transport were also detected, which may collectively support more efficient L-isoleucine biosynthesis (Fig. S8). To further delineate the molecular determinants of the mutant phenotype, key mutated loci in strain cgl-Ile0 were examined at the sequence level (Fig. 1a). From a pathway-centric perspective, single-nucleotide variations (SNVs) were identified in four genes closely connected to L-isoleucine biosynthesis: *aspB*^V346I^ (encoding transaminase), *asd*^P27E^ (encoding aspartate semi-aldehyde dehydrogenase), *brnE*^L87S^ (encoding branched-chain amino acid export transporter BrnE), and *brnF*^R28P^ (encoding branched-chain amino acid export transporter BrnF) (Fig. 1c). Among them AspB and Asd are central enzymes in aspartate-family amino acid biosynthesis, and altered *aspB*/*asd* activity or expression may be expected to reshape flux distribution within the aspartate-family branch. In addition, BrnEF has been reported as an export transporter for branched-chain amino acids; increased *brnEF* activity can enhance product secretion and, at least in part, mitigate end-product feedback inhibition by lowering intracellular branched-chain amino acid levels. Collectively, these genomic features provided a mechanistic rationale for selecting strain cgl-Ile0 as a robust chassis for subsequent rounds of protein and metabolic engineering.

### Protein engineering of rate-limiting enzymes to relieve feedback inhibition

**Fig. 2:**
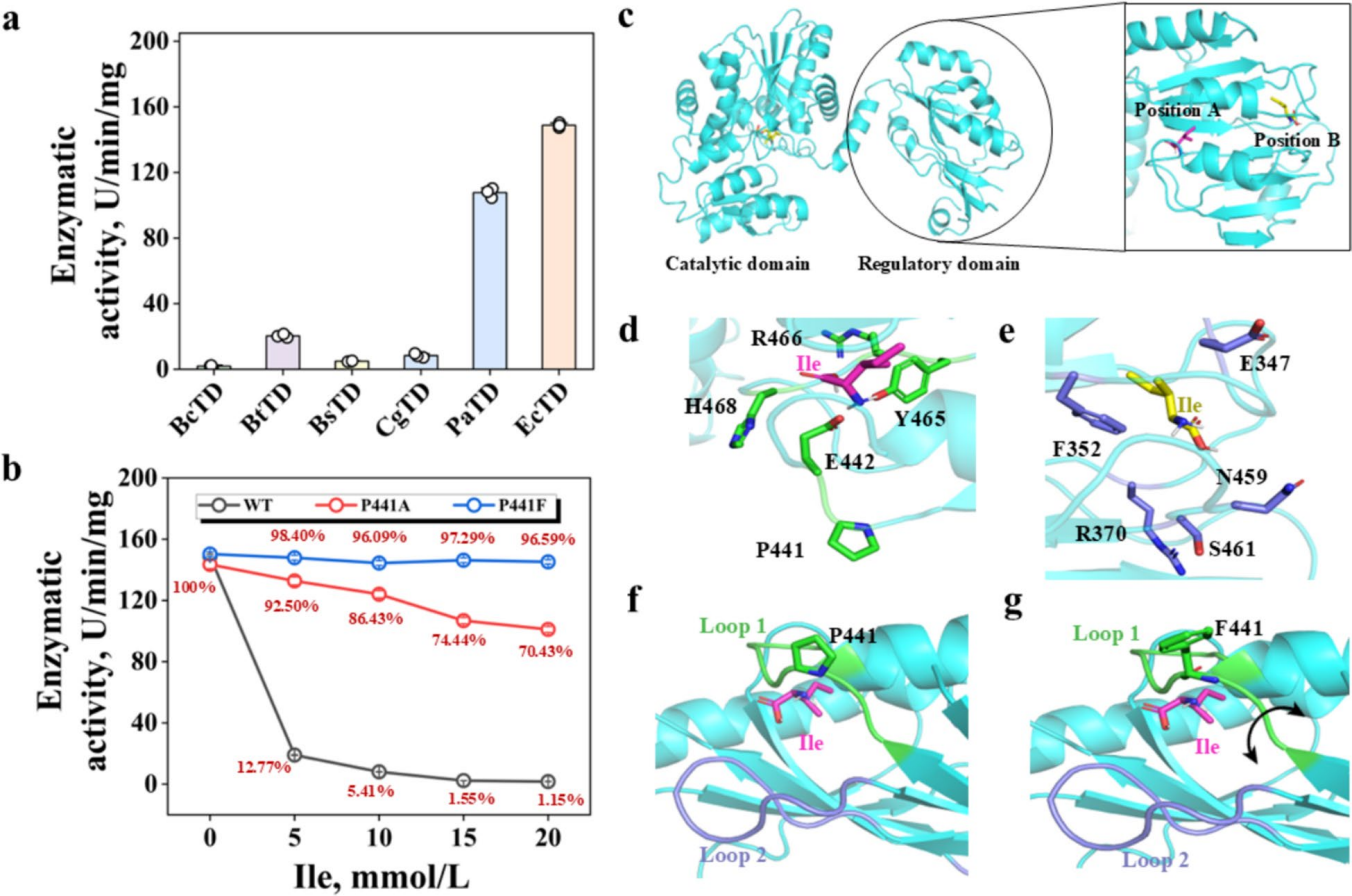
Screening and protein engineering of threonine dehydratase.** a** Screening of threonine dehydratase. *Bc*TD, threonine dehydratase derived from *B. cereus*; *Bt*TD, threonine dehydratase derived from *B. thuringiensis*; *Bs*TD, threonine dehydratase derived from *B. subtilis*; *Cg*TD, threonine dehydratase derived from *C. glutamicum*; *Pa*TD, threonine dehydratase derived from *P. putida*; *Ec*TD, threonine dehydratase derived from *E. coli*. **b** Feedback inhibition tolerance ability of threonine dehydratase and its mutants. **c** Analysis of the protein structure of threonine dehydratase. **d** and **e** Molecular docking of threonine dehydratase and key amino acid residues. **f** and **g** Comparison of the protein structures of threonine dehydratase and its mutants. Values and error bars represent the mean values and standard deviations of three biological repeats

**Fig. 3:**
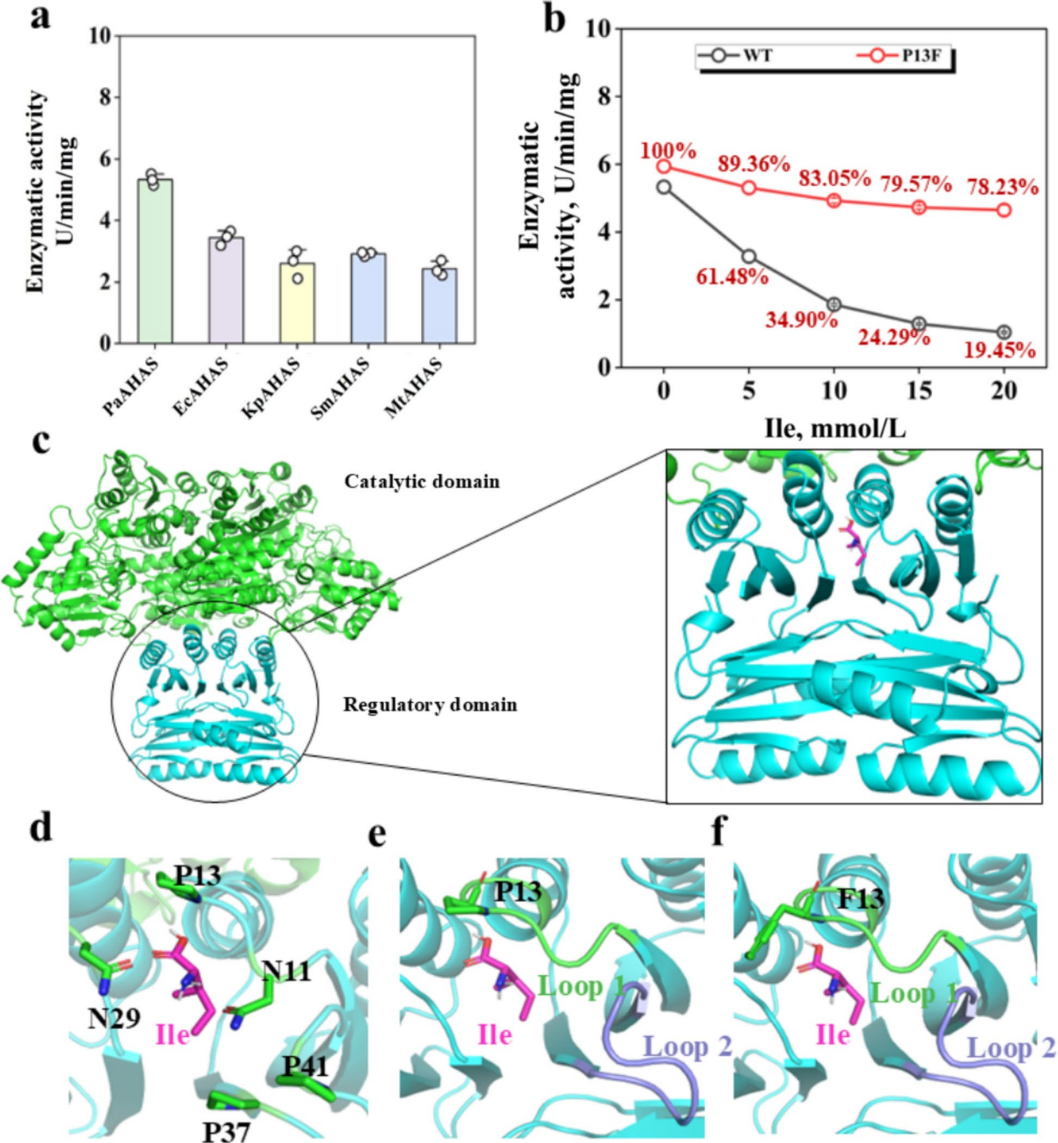
Screening and protein engineering of acetohydroxyacid synthase.** a** Screening of acetolactate synthase. *Pa*AHAS, acetolactate synthetase derived from *P. aeruginosa*; *Ec*AHAS, acetolactate synthetase derived from *E. coli*; *Kp*AHAS, acetolactate synthetase derived from *K. peneumoniae*; *Sm*AHAS, acetolactate synthetase derived from *S. marcescens*; *Mt*AHAS, acetolactate synthetase derived from *M. tuberculosis*. **b** Feedback inhibition tolerance ability of acetolactate synthase and its mutants. **c** Analysis of the protein structure of acetolactate synthase. **d** Molecular docking of acetolactate synthase and key amino acid residues. **e** and **f** Comparison of the protein structures of acetolactate synthase and its mutants. Values and error bars represent the mean values and standard deviations of three biological repeats

TD and AHAS are recognized as key rate-limiting enzymes in the L-isoleucine biosynthetic pathway in *C. glutamicum*. For TD, enzymes from *C. glutamicum*, *E. coli*, *Pseudomonas putida*, *Bacillus thuringiensis*, *Bacillus subtilis*, and *Bacillus cereus* were evaluated, and their phylogenetic relationships are shown in Fig. S10. Among them, the *E. coli* TD (denoted *Ec*TD) exhibited the highest specific activity, reaching 147.45 U/min/mg (Fig. 2a). Among AHAS candidates from *E. coli*, *P. aeruginosa*, *Klebsiella pneumoniae*, *Serratia marcescens*, and *Mycobacterium tuberculosis* were examined, the *P. aeruginosa* enzyme (*Pa*AHAS) showed the highest activity (5.33 U/min/mg; Fig. 3a). In the presence of 5 mmol/L and 20 mmol/L L-isoleucine, *Ec*TD activity decreased to 19.01 and 1.72 U/min/mg, corresponding to 87.23% and 98.95% reductions relative to the uninhibited level (147.45 U/min/mg; Fig. 2b). Similarly, *Pa*AHAS activity declined to 3.28 and 1.04 U/min/mg at 5 mmol/L and 20 mmol/L L-isoleucine, representing 38.52% and 80.55% reductions compared with the uninhibited level (5.33 U/min/mg; Fig. 3b).

Based on structural analysis of *Ec*TD (PDB: 1EDJ), the enzyme was found to comprise a regulatory domain and a catalytic domain. L-Isoleucine was predicted to act as an allosteric effector by binding to either effector position A or B within the regulatory domain, thereby modulating dimer formation and enzymatic activity (Fig. 2c). Guided by the predicted effector-binding region, ten residues (P441, E442, Y465, R466, H468, E437, F352, R370, N459, and S461) were selected for alanine scanning (Fig. 2d–e). Among the resulting variants, *Ec*TD(P441A) maintained an activity comparable to the wild-type enzyme under uninhibited conditions and, importantly, retained 92.50% and 70.43% residual activity in the presence of 5 mmol/L and 20 mmol/L L-isoleucine, respectively (Fig. 2b and Fig. S11). Saturation mutagenesis at position 441 further identified *Ec*TD(P441F) as a superior feedback inhibition-resistant variant, preserving 98.40% and 96.59% activity even at 5 mmol/L and 20 mmol/L L-isoleucine (Fig. S12). Structural analysis of the mutant *Ec*TD(P441F) revealed that this mutation reduces the binding affinity of the effector to the regulatory domain by influencing the conformational dynamics of the key loop (Fig. 2f and g).

A search of UniProt for structurally related AHAS crystal structures revealed strong similarity to the *Arabidopsis* AHAS (Fig. 3c and Fig. S13). Based on this similarity, L-isoleucine was docked into the ACT regulatory domain to identify candidate residues near the putative effector-binding pocket, including N11, P13, N29, P37, and P4 (Fig. 3d). These positions were subsequently subjected to aromatic/large-residue (ALF) scanning to introduce steric perturbations. Among the variants, *Pa*AHAS(P13F) exhibited near wild-type activity in the absence of effector and maintained 89.36% and 78.23% residual activity in the presence of 5 mmol/L and 20 mmol/L L-isoleucine, respectively (Fig. 3b). Structural analysis of the mutant *Pa*AHAS(P13F) revealed that this mutation alleviates feedback inhibition by increasing spatial hindrance between the effector and the regulatory domain.

Building on the identification of two anti-feedback inhibitory mutants, this study enhanced the isoleucine synthesis pathway by increasing the copy number of the target gene. Specifically, an additional chromosomal copy of *E. coli ilvA*^*P441F*^ and *Pa ilvIH*^*P13F*^ was integrated at the NCgl0008 and NCgl0032 loci, respectively, yielding strain cgl-Ile1. In a 5-L fermenter (48 h), strain cgl-Ile1 produced 16.07 g/L L-isoleucine with a yield of 0.14 g/g and a productivity of 0.33 g/L/h, corresponding to improvements of 39.50%, 27.27%, and 37.50% relative to strain cgl-Ile0 (11.52 g/L, 0.11 g/g, and 0.24 g/L/h, respectively) (Fig. S14).

### Modular engineering for efficient L-isoleucine biosynthesis

**Fig. 4:**
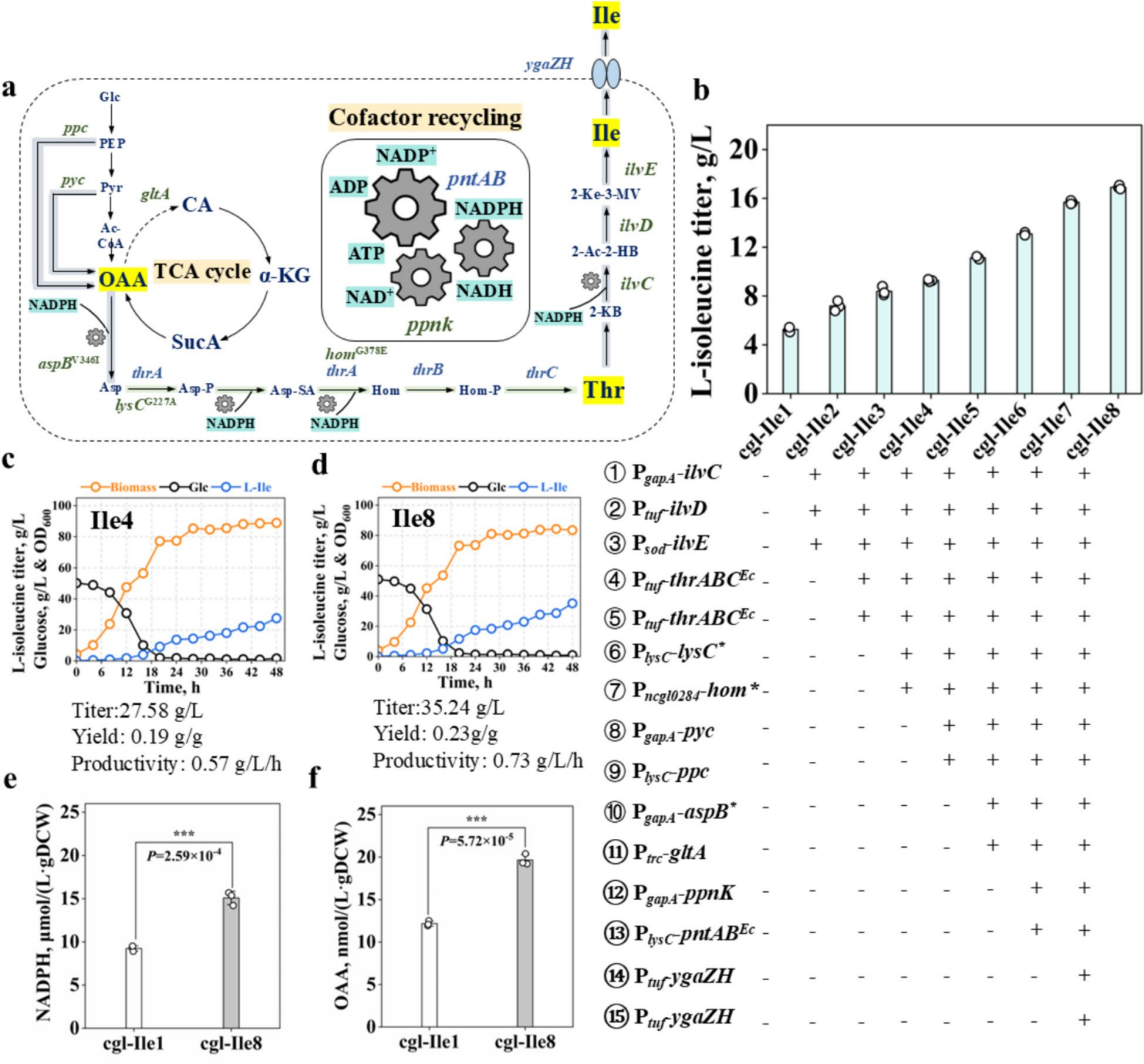
Modular engineering of strain cgl-Ile1.** a** The synthetic metabolic pathway of isoleucine. Glc, glucose; PEP, phosphoenolpyruvate; Ac-CoA, acetyl coenzyme A; OAA, oxaloacetate; Asp, aspartate; Asp-P, aspartate-phosphate; Asp-SA, aspartate-semialdehyde; Hom, homoserine; Hom-P, homoserine-phosphate; Thar, threonine; 2-KB, acetohydroxy butyrate; 2-Ac-2-HB, dihydrexymethyl valerate; 2-Ke-3-MV, ketomethyl valerate; *ppc*, encoding phosphoenolpyruvate carboxylase derived from *C. glutamicum*; *pyc*, encoding pyruvate carboxylase derived from *C. glutamicum*; *gltA*, encoding citrate synthase derived from *C. glutamicum*; *aspB*^V346I^, encoding mutant of aspartate aminotransferase derived from *C. glutamicum*; *asd*^P27E^, encoding mutant of aspartate-semialdehyde dehydrogenase derived from *C. glutamicum*; *lysC*^G227A^, encoding mutant of aspartate kinase derived from *C. glutamicum*; *hom*^G378E^, encoding mutant of homoserine dehydrogenase derived from *C. glutamicum*; *ilvC*, encoding ketol-acid reductoisomerase derived from *C. glutamicum*; *ilvD*, encoding dihydroxy-acid dehydratase derived from *C. glutamicum*; *ilvE*, encoding branched-chain-amino-acid aminotransferase derived from *C. glutamicum*; *ppnK*, encoding NAD kinase derived from *C. glutamicum*; *thrA*, encoding fused aspartate kinase/homoserine dehydrogenase 1 derived from *E. coli*; *thrB*, encoding homoserine kinase derived from *E. coli*; *thrC*, encoding threonine synthase derived from *E. coli*; *ygaZH*, encoding branched-chain amino acids exporter. **b** The isoleucine titer and gene targets of strain cgl-Ile1 – 8. **c** The fermentation curve of strain cgl-Ile4 in a 5-L fermenter. **d** The fermentation curve of strain cgl-Ile8 in a 5-L fermenter. **e** The intracellular cofactor NADPH content of strains cgl-Ile1 and cgl-Ile8.** f** The intracellular oxaloacetate content of strains cgl-Ile1 and cgl-Ile8. Statistical significance is denoted as ^*^P < 0.05, ^**^P < 0.01, and ^***^P < 0.001. Values and error bars represent the mean values and standard deviations of three biological repeats

Accordingly, the cellular network was partitioned into four functional modules for coordinated regulation: (i) the L-isoleucine biosynthesis module (aspartate → L-isoleucine) was composed of core pathway genes, including *ilvC*, *ilvD*, *ilvE*, *hom*, and *lysC*, together with the heterologous *thrABC* operon from *E. coli*; (ii) The oxaloacetate (OAA) supply module was constructed to strengthen anaplerotic replenishment and included *pyc*, *ppc*, *aspB*, and *gltA*; (iii) To increase reducing-power availability, the cofactor supply module was designed to enhance NADPH generation via *ppnK* and introduction of the *E. coli pntAB* transhydrogenase operon; (iv) Finally, the transport module (Fig. 4a) was implemented to improve substrate uptake and/or intracellular metabolite trafficking through the *E. coli ygaZH* operon.

Using promoter engineering as the primary control lever, stepwise module optimization was performed (Fig. 4 and Figs. S15–S19). (1) For the L-isoleucine biosynthesis module, three well-characterized promoters (P_*tuf*_, P_*sod*_, and P_*gapA*_) were combinatorially deployed to modulate *ilvC*, *ilvD*, and *ilvE* expression in the strain cgl-Ile1, generating strains cgl-Ile1-1 to cgl-Ile1-27 (Fig. S15). Among them, strain cgl-Ile1-21 (P_*gapA*_-*ilvC*–P_*tuf*_-*ilvD*–P_*sod*_-*ilvE*) was the best, producing 7.18 g/L L-isoleucine, a 34.21% increase over strain cgl-Ile1 (5.25 g/L). This strain was designated strain cgl-Ile2 (Fig. 4b and Fig. S15). (2) To strengthen precursor supply toward L-threonine and subsequent L-isoleucine formation, two chromosomal copies of the *E. coli thrABC* operon were integrated into cgl-Ile2 at the NCgl0056 and NCgl0070 loci, yielding strain cgl-Ile3 (Fig. 4b). This modification increased the L-isoleucine titer to 8.39 g/L, 16.85% higher than that of strain cgl-Ile2 (7.18 g/L) (Fig. 4b). To further alleviate feedback constraints at the precursor layer, a feedback-resistant homoserine dehydrogenase variant (*hom*^G378E^) and a feedback-resistant aspartokinase variant (*lysC*^G277A^) were introduced, and their expression was combinatorically tuned using P_*lysC*_, P_ncgl0281_, and P_*sod*_, and strains cgl-Ile3-1 to cgl-Ile3-8 were constructed accordingly (Fig. S16). Among these strains, strain cgl-Ile3-1 (P_*lysC*_-*hom*^*G378E*^–P_*ncgl0281*_-*lysC*^G277A^) achieved the highest titer (9.27 g/L), a 15.85% increase over cgl-Ile3, and was renamed strain cgl-Ile4 (Fig. 4b and Fig. S16).

Based on strain cgl-Ile4, the OAA supply, cofactor supply, and transport modules were subsequently optimized (Fig. 4 and Figs. S17–S19). For the OAA supply module, *pyc* and *ppc* expression was tuned in strain cgl-Ile4 using promoters P_*gapA*_, P_*lysC*_, and P_*sod*_, generating strains cgl-Ile4-1 to cgl-Ile4-8 (Fig. S17), among them, strain cgl-Ile4-3 (P_*gapA*_-*pyc*–P_*lysC*_-*ppc*) produced 11.13 g/L L-isoleucine in shake flasks, was higher 20.06% than strain cgl-Ile4, and was designated cgl-Ile5 (Fig. 4b and Fig. S17). Subsequently, *aspB* and *gltA* expression was tuned using P_*gapA*_, P_*sod*_, and P_*trc*_, generating strains cgl-Ile5-1 to cgl-Ile5-8 (Fig. S18). The highest titer was achieved by cgl-Ile5-5 (P_*gapA*_*-aspB–*P_*trc*_*-gltA*), reaching 13.09 g/L, which corresponded to a 14.57% improvement relative to cgl-Ile5; this strain was renamed cgl-Ile6 (Fig. 4b and Fig. S19). To meet the high NADPH demand of L-isoleucine biosynthesis, the cofactor supply module was engineered by integrating the *E. coli pntAB* operon, encoding a membrane-bound transhydrogenase for NADPH regeneration, into the NCgl0992 locus of cgl-Ile6, followed by combinatorial tuning of *ppnK* (NAD^+^ kinase) and *pntAB* expression using P_*ncgl0281*_, P_*gapA*_, and P_*lysC*_ to generate strains cgl-Ile6-1 to cgl-Ile6-8 (Fig. S17). Among these strains, strain cgl-Ile6-4 (P_*gapA*_*-ppnK–*P_*lysC*_*-pntAB*) produced 15.68 g/L L-isoleucine and was designated cgl-Ile7 (Fig. 4b and Fig. S17). Finally, the transport module was strengthened by introducing an additional chromosomal copy of the *E. coli ygaZH* operon at each of the NCgl0077 and NCgl0092 loci, yielding strain cgl-Ile8. This modification further increased the L-isoleucine titer to 16.91 g/L, representing a 7.84% improvement over cgl-Ile7 (15.68 g/L) (Fig. 4b).

Bioreactor fermentations were conducted for strain cgl-Ile4 and cgl-Ile8 in 5-L fermenters over 48 h (Fig. 4). Strain cgl-Ile4 produced 27.58 g/L L-isoleucine with a yield of 0.19 g/g and a productivity of 0.57 g/L/h, whereas strain cgl-Ile8 reached 35.24 g/L with a yield of 0.23 g/g and a productivity of 0.73 g/L/h (Fig. 4c). Compared to the initial engineered strain cgl-Ile1 (16.07 g/L, 0.14 g/g, 0.33 g/L/h), cgl-Ile4 showed 1.72-, 1.21-, and 1.73-fold increases in titer, yield, and productivity, respectively, while cgl-Ile8 achieved 2.19-, 1.64-, and 2.21-fold improvements (Fig. 4d). Consistent with the design rationale, intracellular metabolite profiling at 36 h revealed expanded pools of both OAA and NADPH in cgl-Ile8 (19.64 nmol/g DCW and 15.09 μmol/g DCW, respectively) compared to cgl-Ile1 (12.17 nmol/g DCW and 9.24 μmol/g DCW), representing increases of 61.38% and 63.31%, respectively (Fig. 4e–f). These data collectively indicate that the modular rewiring of precursor supply and cofactor regeneration enhanced intracellular OAA and NADPH availability, thereby promoting L-isoleucine biosynthesis and improving overall fermentation performance.

### Optimization of fermentation process in 5-L and 50-L fermenter

**Fig. 5:**
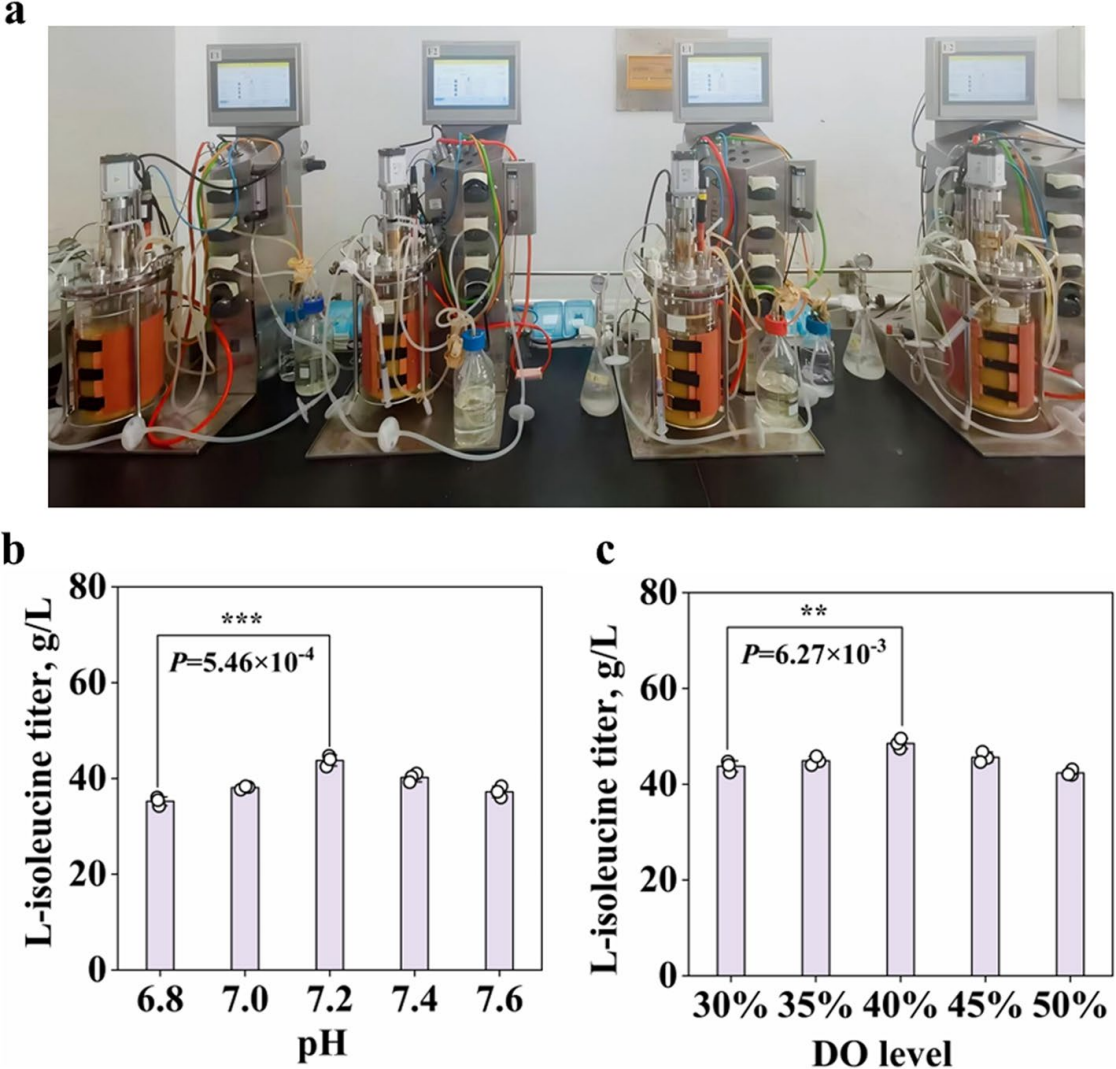
Optimization of the fermentation process for strain cgl-Ile8.** a** The fermentation process of strain cgl-Ile8 in a 5-L fermenter. **b** Optimization of pH during the fermentation process of strain cgl-Ile8. **c** Optimization of dissolved oxygen levels during the fermentation process of strain cgl-Ile8. Statistical significance is denoted as ^*^P < 0.05, ^**^P < 0.01, and ^***^P < 0.001. Values and error bars represent the mean values and standard deviations of three biological repeats

To further improve the fermentation performance of strain cgl-Ile8, two key process parameters—pH and DO—were optimized in 5-L fermenter (Fig. 5 and Fig. S20). First, pH was evaluated at 6.8, 7.0, 7.2, 7.4, and 7.6 across three parallel batches (Fig. 5a); under these conditions, L-isoleucine titers of 35.24, 38.09, 43.75, 40.20, and 37.20 g/L were obtained, respectively (Fig. 5b). The highest titer was achieved at pH 7.2, representing a 24.15% increase relative to pH 6.8. At pH 7.2, DO was subsequently controlled at 30%, 35%, 40%, 45%, and 50% (Fig. 5a), resulting in L-isoleucine titers of 43.75, 44.87, 48.49, 45.63, and 42.37 g/L, respectively (Fig. 5c). The highest titer was obtained at a DO setpoint of 40% (48.49 g/L), which represented a 10.83% increase compared with DO at 30%. Based on these results, pH 7.2 and a DO setpoint of 40% were identified as the optimal conditions for strain cgl-Ile8 in 5-L fermenters. Process scalability was then assessed in a 50-L fermenter, in which the titer, yield, and productivity increased to 50.12 g/L, 0.32 g/g, and 1.04 g/L/h, respectively (Fig. S20). To the best of our knowledge, this represents the highest reported L-isoleucine production achieved in *C. glutamicum* to date.

## Discussion

Our results indicate that the limitations of L-isoleucine biosynthesis in *C. glutamicum* are not attributable to a single bottleneck, but rather arise from the coupled constraints of enzymatic regulation, metabolic flux distribution, and redox–precursor balance. In this study, *C. glutamicum* ATCC 13032 was used as the starting strain, and an industrial L-isoleucine producer (cgl-Ile8) was constructed through an integrated engineering workflow that combined biosensor-assisted ARTP mutagenesis, protein engineering of key rate-limiting enzymes, and modular, promoter-guided regulation of metabolic flux. Following bioprocess optimization, the titer, yield and productivity of L-isoleucine by strain cgl-Ile8 in a 50-L fermenter was 50.12 g/L, 0.32 g/g, and 1.04 g/L/h, respectively. To the best of our knowledge, this represents the highest reported L-isoleucine production performance in *C. glutamicum* to date, providing a robust foundation for industrial-scale manufacturing.

Similar to L-isoleucine, microbial fermentation is the primary approach for L-leucine production, with *E. coli* and *C. glutamicum* as the dominant industrial hosts. To date, the highest reported L-leucine titer in *E. coli* has reached 63.29 g/L [9], which is significantly higher than the 28.47 g/L achieved in *C. glutamicum* [36]. This difference is consistent with their respective performances in L-isoleucine production, indicating a clear advantage of *E. coli* in branched-chain amino acid biosynthesis. This superiority of *E. coli* can be explained by several key factors. First, although both organisms share highly similar biosynthetic pathways characterized by long reaction sequences, multiple metabolic nodes, and competition with lysine and methionine branches, *C. glutamicum* is subject to more stringent feedback inhibition, which is more difficult to relieve and restricts carbon flux toward product formation. Second, despite identical NADPH requirements, *C. glutamicum* experiences stronger cofactor competition due to its naturally robust capacity for synthesizing multiple amino acids, resulting in a more pronounced NADPH limitation. In addition, *E. coli* benefits from a more flexible regulatory network and a well-established genetic toolbox, enabling more efficient metabolic rewiring and dynamic pathway optimization. Nevertheless, *C. glutamicum* possesses distinct industrial advantages, including its GRAS status, high robustness, and superior tolerance to osmotic stress and product accumulation, which contribute to stable large-scale fermentation. Therefore, while *E. coli* currently achieves higher production titers, *C. glutamicum* remains a promising alternative host with significant potential for future development through advanced metabolic engineering strategies.

Using the pSenIleBLP–FACS HTS platform, the model strain *C. glutamicum* ATCC 13032 was subjected to ARTP mutagenesis, enabling the isolation of a genetically stable L-isoleucine-producing strain (cgl-Ile0), which was subsequently characterized in terms of its genetic background and fermentation performance. Efficient identification of amino-acid overproducers from large mutant libraries critically depends on screening strategies that are high-throughput, accurate, and rapid. Current approaches for developing amino-acid overproducing strains can be broadly classified into three categories: (i) auxotrophy-based selection [30], (ii) translation-coupled selection [37], and (iii) biosensor-guided selection [38]. Auxotrophy-based schemes generally lack the resolution required to reliably discriminate high-producing single cells, whereas translation-coupled strategies can impose unintended perturbations beyond protein synthesis. In addition, selection escape can arise through multiple adaptive routes, and these approaches are often constrained by the cost, instability, and toxicity of amino-acid analogs. By contrast, biosensor-guided screening is operationally straightforward, highly sensitive, and compatible with large screening throughput, and has been successfully applied to isolate high-producing strains for L-lysine, L-threonine, L-arginine, L-isoleucine, and L-tryptophan. For example, a growth-coupled biosensor (pABIO10) enabled rapid isolation of an industrially *E. coli* strain ARG28 from a mutant library; subsequent systematic rewiring of the L-arginine biosynthetic pathway, the tricarboxylic acid (TCA) cycle, and the arginine export system yielded strain ARG28, which could produce 132 g/L L-arginine with 0.51 g/g yield, and 2.75 g/L/h productivity in a 5-L fermenter, representing the highest level reported to date [39]. In this work, the sensing range was expanded and operational usability was improved by optimizing the plasmid backbone, incorporating the branched-chain amino-acid exporter *brnEF*, and replacing the replicon with a temperature-sensitive variant to enable plug-and-play plasmid curing after screening. Using the resulting pSenIleBLP–FACS HTS platform, a genetically stable strain capable of L-isoleucine overaccumulation (cgl-Ile0) was isolated from the ARTP mutant library. Comparative genomic analysis further identified four key mutations—*aspB*^V346I^, *asd*^P27E^, *brnE*^L87S^, and *brnF*^R28P^—that were closely associated with the improved phenotype. In 5-L fermentation, the titer, yield, and productivity of strain cgl-Ile0 were increased to 11.52 g/L, 0.11 g/g, and 0.24 g/L/h, respectively.

In this study, structure-guided protein engineering effectively alleviated end-product feedback inhibition of two key rate-limiting enzymes in the L-isoleucine pathway—TD and AHAS to yield two optimal variants, *Ec*TD(P441F) and *Pa*AHAS(P13F). Upon supplementation with 5 mmol/L and 20 mmol/L L-isoleucine, residual activities of *Ec*TD(P441F) were maintained at 98.40% and 96.59%, respectively, whereas *Pa*AHAS(P13F) retained 89.36% and 78.23% activity, respectively. Incorporation of these variants generated strain cgl-Ile1, in which the titer, yield, and productivity were increased by 39.50%, 27.27%, and 37.50%, respectively, compared with cgl-Ile0. To overcome end-product inhibition of key enzymes during amino-acid biosynthesis, six general strategies have been developed: (i) sequence-guided protein engineering, (ii) genome mining from mutant libraries, (iii) biosensor-assisted directed evolution, (iv) screening and replacement with highly active heterologous enzymes, (v) metabolic–physiological regulation via the PII signal transduction system, and (vi) structure-guided protein engineering [40]. Among these, structure-guided protein engineering enables rational optimization of catalytic efficiency, stability, and/or substrate specificity, thereby improving amino-acid titer, yield, and productivity. For example, homoserine kinase (HK) from *C. glutamicum* was engineered to an optimal variant, HK(R240I), which exhibited an 8.2-fold decrease in substrate affinity and a 4.3-fold increase in catalytic efficiency (kcat/Km), resulting in an increase in serine titer from 20.8 g/L to 26.8 g/L. In another study, N-acetylglutamate kinase (NAGK) in the L-arginine biosynthetic pathway was engineered to variant NAGK EH3, which displayed a 382-fold increase in the arginine half-inhibitory concentration and a 1.73-fold increase in specific activity relative to the wild-type enzyme. Consequently, the L-arginine titer reached 61.2 g/L, representing a 41.8% improvement over the parental strain.

In this study, modular pathway design enabled fine-grained regulation of the L-isoleucine biosynthesis module, the oxaloacetate (OAA) supply module, the cofactor supply module, and the transport module, resulting in strain cgl-Ile8, in which the titer, yield, and productivity of L-isoleucine reached 35.24 g/L, 0.23 g/g, and 0.73 g/L/h, respectively. As a core methodology in metabolic engineering, modular engineering decomposes complex metabolic networks into functionally separable units that can be assembled and optimized through standardized interfaces, thereby facilitating efficient biosynthesis. This design paradigm simplifies pathway construction, accelerates iterative optimization, and provides a practical route to resolve metabolic bottlenecks and improve production performance. To date, modular engineering has enabled high-level biosynthesis of diverse products, including vinblastine [41], shikimate [42], tryptophan [43], threonine [44], orotate [45], chlorogenic acid [46], β-carotene [47], vitamin B_5_ [48], and vitamin K_2_ [49]. For example, in *E. coli*, combinatorial optimization of four modules—PEP supply, E4P supply, core pathway enzymes, and basal resource allocation—raised shikimate production to 78.4 g/L in strain SA05. In another study, reinforcement of the R-pantothenate biosynthetic pathway, rewiring of TCA-cycle carbon flux, and introduction of a one-carbon module to enhance precursor supply enabled strain DPAC4 to achieve a vitamin B5 titer, yield, and productivity of 148.31 g/L, 0.43 g/g, and 1.54 g/L/h, respectively.

## Conclusion

In this study, a comprehensive, mechanism-based metabolic engineering strategy was developed to enhance L-isoleucine production by *C. glutamicum*. This strategy integrates three core technologies: biosensor-assisted HTS, protein engineering of key rate-limiting enzymes, and modular regulation of metabolic flux, thereby systematically solving key bottlenecks such as complex synthetic pathways, strong feedback inhibition, and imbalance of precursor and cofactor supply. This work bridges mechanistic enzymology and systems metabolic engineering, offering a generalizable paradigm for the rational design of microbial cell factories for feed amino acids.

## Supplementary Information

Additional file 1: Fig. S1. Evaluation of the dynamic response range of the isoleucine biosensor. Fig. S2. ARTP radiation time and cell mortality rate. Fig. S3. Flow cytometry sorting of mutagenized strains. Fig. S4. The isoleucine titer of mutagenic strains. Fig. S5. Genetic stability evaluation of mutagenic strains. Fig. S6. Evaluation of the fermentation performance of strain cgl-Ile0 Fig. S7. Genetic background analysis of strain cgl-Ile0. Fig. S8. Distribution of KEGG functional notes in the mutated genes of strain cgl-Ile0. Fig. S9. Distribution of GO functional notes in the mutated genes of strain cgl-Ile0. Fig. S10. Evolutionary tree of threonine dehydrataseand acetohydroxyacid synthase. Fig. S11. Alanine scanning of the key amino acid residues of threonine dehydratase. Fig. S12. Saturated mutations of the key amino acid residues of threonine dehydratase. Fig. S13. ALF scanning of the key amino acid residues of acetyl lactate synthase. Fig. S14. Evaluation of the fermentation performance of the strain cgl-Ile1. Fig. S15. Evaluation of the fermentation of strain cgl-Ile1-1 ~ 27 in a 500-mL shake flask. Fig. S16. Evaluation of the fermentation of strain cgl-Ile3-1 ~ 8 in a 500-mL shake flask. Fig. S17. Evaluation of the fermentation of strain cgl-Ile4-1 ~ 8 in a 500-mL shake flask. Fig. S18. Evaluation of the fermentation of strain cgl-Ile5-1 ~ 8 in a 500-mL shake flask. Fig. S19. Evaluation of the fermentation of strain cgl-Ile6-1 ~ 8 in a 500-mL shake flask. Fig. S20. Evaluation of the fermentation performance of strain cgl-Ile8 in a 50-L fermenter. Table S1. Strains & Plasmids used in this study. Table S2. Genomic integration sites used in this study. Table S3. Heterologous genes used in this study. Table S4. Genetic modification targets used in this study. Table S5. Promoters used in this study. Table S6. Replacement of the promoters of the target genes. Table S7. Promoter strength reference. Table S8. Primers used in this study. Table S9. The compositions of the Seed solid medium. Table S10. The compositions of the shaking flask medium. Table S11. The compositions of the fermentation tank medium. Table S12. The compositions of LBGB. Table S13. The compositions of the BT medium. Method S1. Measurement of intracellular cofactors. Method S2. HPLC mobile phases and gradient program.

## Data Availability

Data will be made available on request.

## Abbreviations

AHAS: Acetohydroxyacid synthase
ARTP: Atmospheric and room-temperature plasma
DO: Dissolved oxygen
FACS: Fluorescence-activated cell sorting
HTS: High-throughput screening
OAA: Oxaloacetate
OPA: O-phthaldialdehyde
TCA: Tricarboxylic acid
TD: Threonine dehydratase
