# Supplementary material for: Systems metabolic engineering of Corynebacterium glutamicum for efficient production of L-isoleucine
Source: J Anim Sci Biotechnol. 2026 Jul 8;17:142. doi: 10.1186/s40104-026-01459-y (PMC13343988; doi:10.1186/s40104-026-01459-y)
Supplement: Supplementary file 1 — Additional file 1: Fig. S1. Evaluation of the dynamic response range of the isoleucine biosensor. Fig. S2. ARTP radiation time and cell mortality rate. Fig. S3. Flow cytometry sorting of mutagenized strains. Fig. S4. The isoleucine titer of mutagenic strains. Fig. S5. Genetic stability evaluation of mutagenic strains. Fig. S6. Evaluation of the fermentation performance of strain cgl-Ile0 Fig. S7. Genetic background analysis of strain cgl-Ile0. Fig. S8. Distribution of KEGG functional notes in the mutated genes of strain cgl-Ile0. Fig. S9. Distribution of GO functional notes in the mutated genes of strain cgl-Ile0. Fig. S10. Evolutionary tree of threonine dehydrataseand acetohydroxyacid synthase. Fig. S11. Alanine scanning of the key amino acid residues of threonine dehydratase. Fig. S12. Saturated mutations of the key amino acid residues of threonine dehydratase. Fig. S13. ALF scanning of the key amino acid residues of acetyl lactate synthase. Fig. S14. Evaluation of the fermentation performance of the strain cgl-Ile1. Fig. S15. Evaluation of the fermentation of strain cgl-Ile1-1 ~ 27 in a 500-mL shake flask. Fig. S16. Evaluation of the fermentation of strain cgl-Ile3-1 ~ 8 in a 500-mL shake flask. Fig. S17. Evaluation of the fermentation of strain cgl-Ile4-1 ~ 8 in a 500-mL shake flask. Fig. S18. Evaluation of the fermentation of strain cgl-Ile5-1 ~ 8 in a 500-mL shake flask. Fig. S19. Evaluation of the fermentation of strain cgl-Ile6-1 ~ 8 in a 500-mL shake flask. Fig. S20. Evaluation of the fermentation performance of strain cgl-Ile8 in a 50-L fermenter. Table S1. Strains & Plasmids used in this study. Table S2. Genomic integration sites used in this study. Table S3. Heterologous genes used in this study. Table S4. Genetic modification targets used in this study. Table S5. Promoters used in this study. Table S6. Replacement of the promoters of the target genes. Table S7. Promoter strength reference. Table S8. Primers used in this study. Table S9. The compositions [file 40104_2026_1459_MOESM1_ESM.docx]

**Supplementary figures**


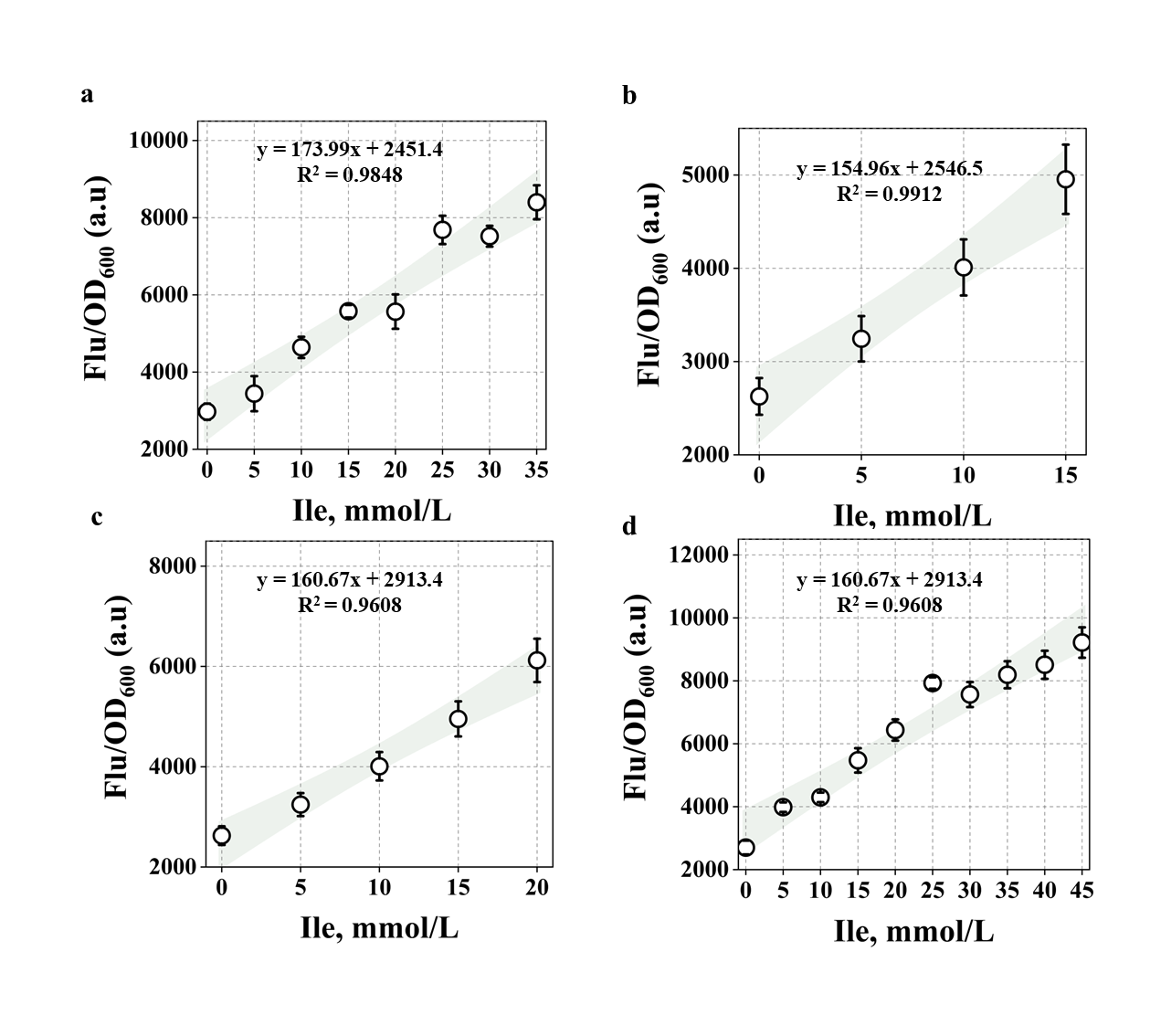


Fig. S1 Evaluation of the dynamic response range of the isoleucine biosensor. **a** Dynamic response range of pECXK99E-Sensor^Ile^. **b** Dynamic response range of pXMJ19-Sensor^Ile^. **c** Dynamic response range of pZK001-Sensor^Ile^. **d** Dynamic response range of pSenIleBP. Values and error bars represent the mean values and standard deviations of three biological repeats


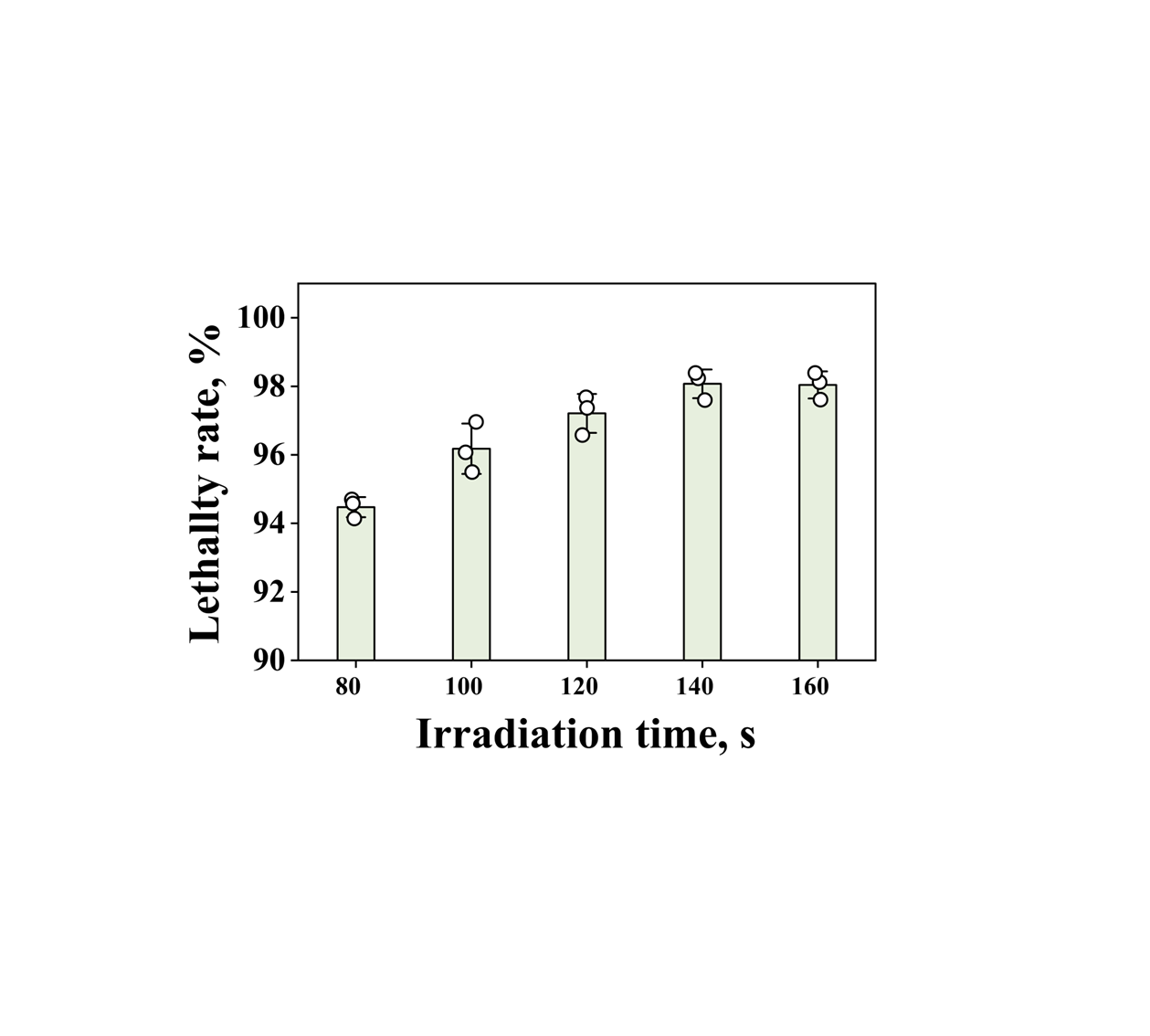


**Fig. S2** ARTP radiation time and cell mortality rate. Values and error bars represent the mean values and standard deviations of three biological repeats


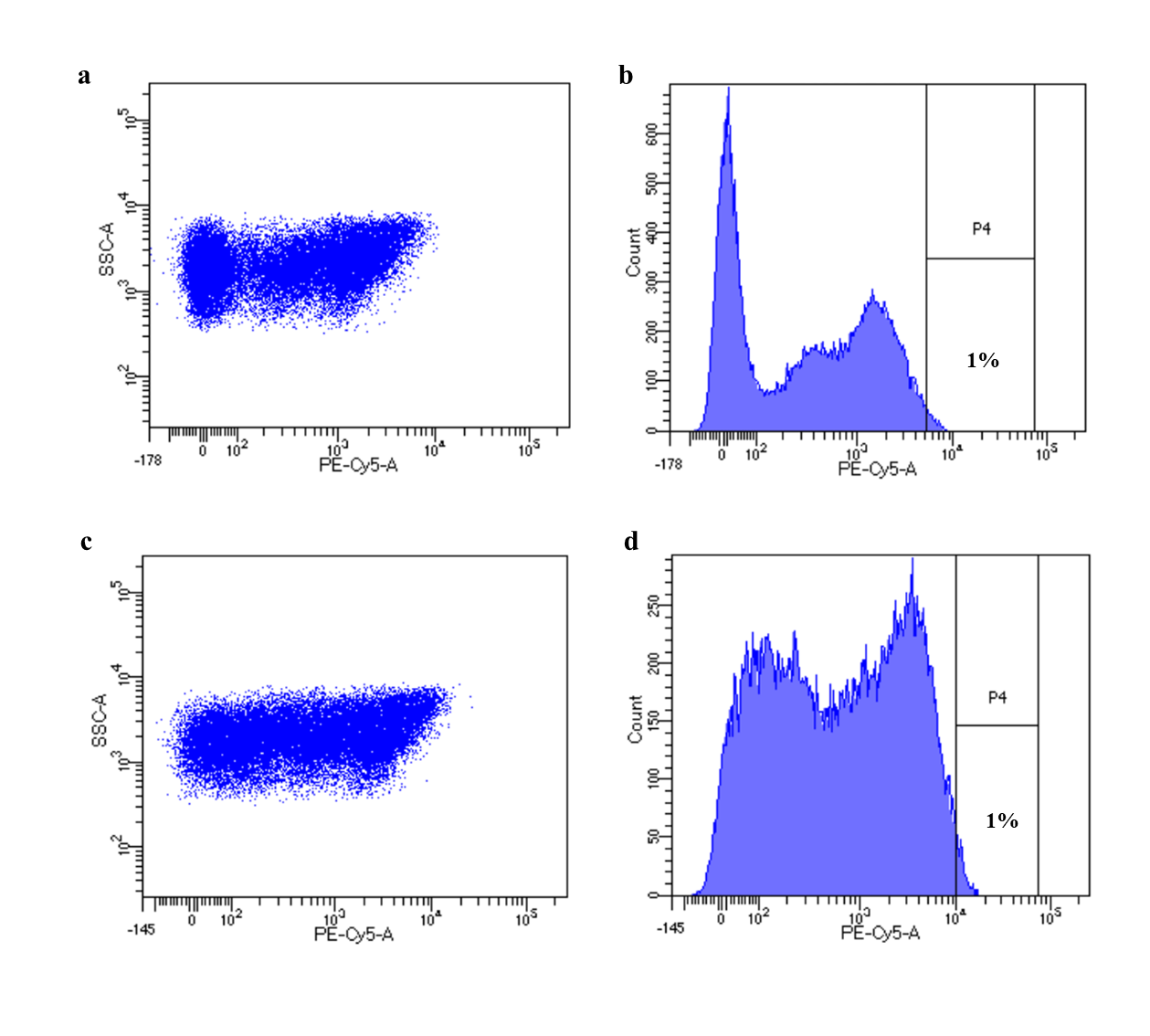


**Fig. S3** Flow cytometry sorting of mutagenized strains. **a** and **b** Flow cytometry sorting results after the first ARTP mutagenesis. **c** and **d** Flow cytometry sorting results after the second ARTP mutagenesis. For each round, the top 1% of the fluorescent population was selected as the sorting gate and collected for plating. The first selected population was recovered by cultivation and then subjected to a second round of ARTP mutagenesis before FACS-based screening


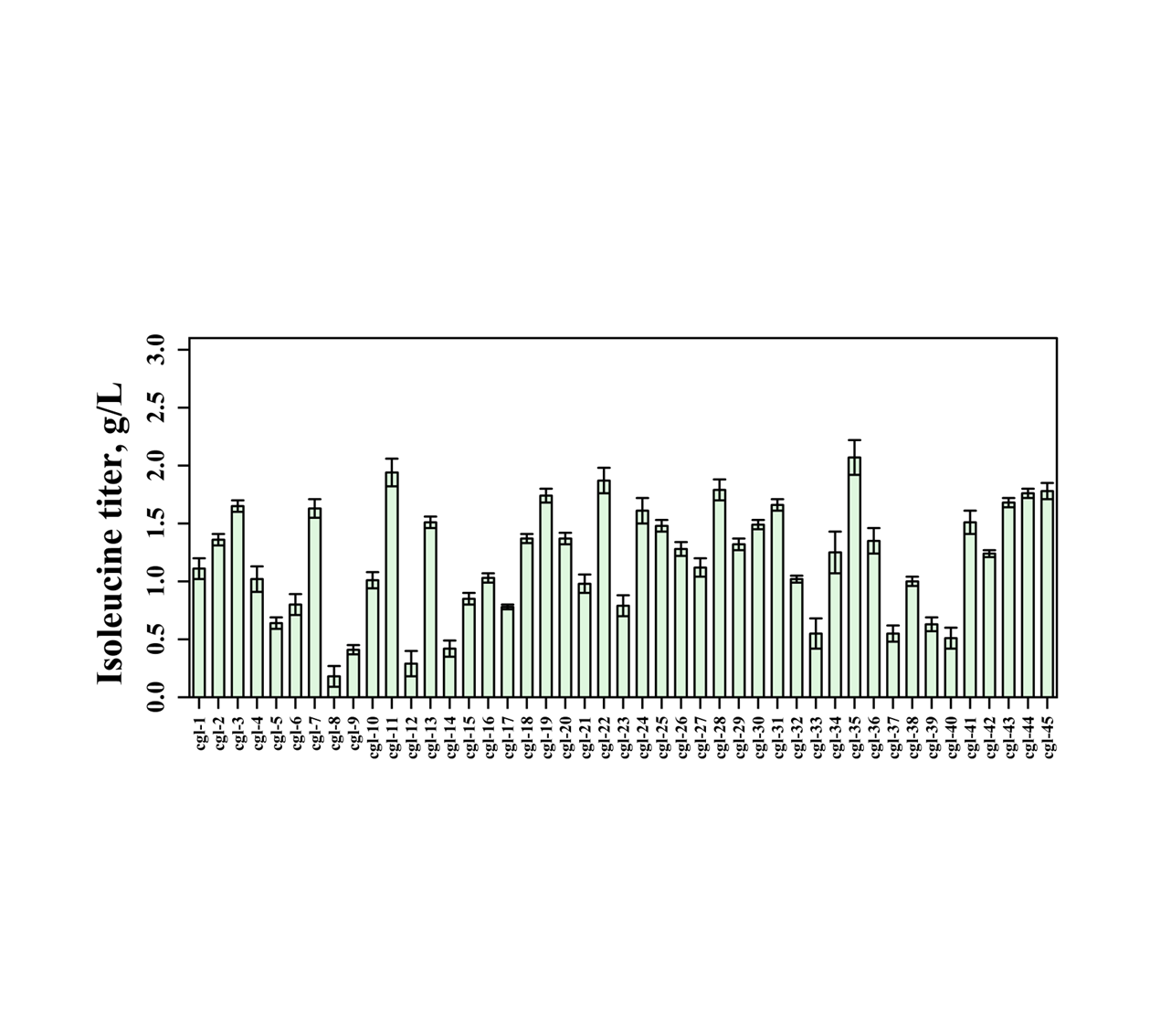


**Fig. S4** The isoleucine titer of mutagenic strains. Values and error bars represent the mean values and standard deviations of three biological repeats


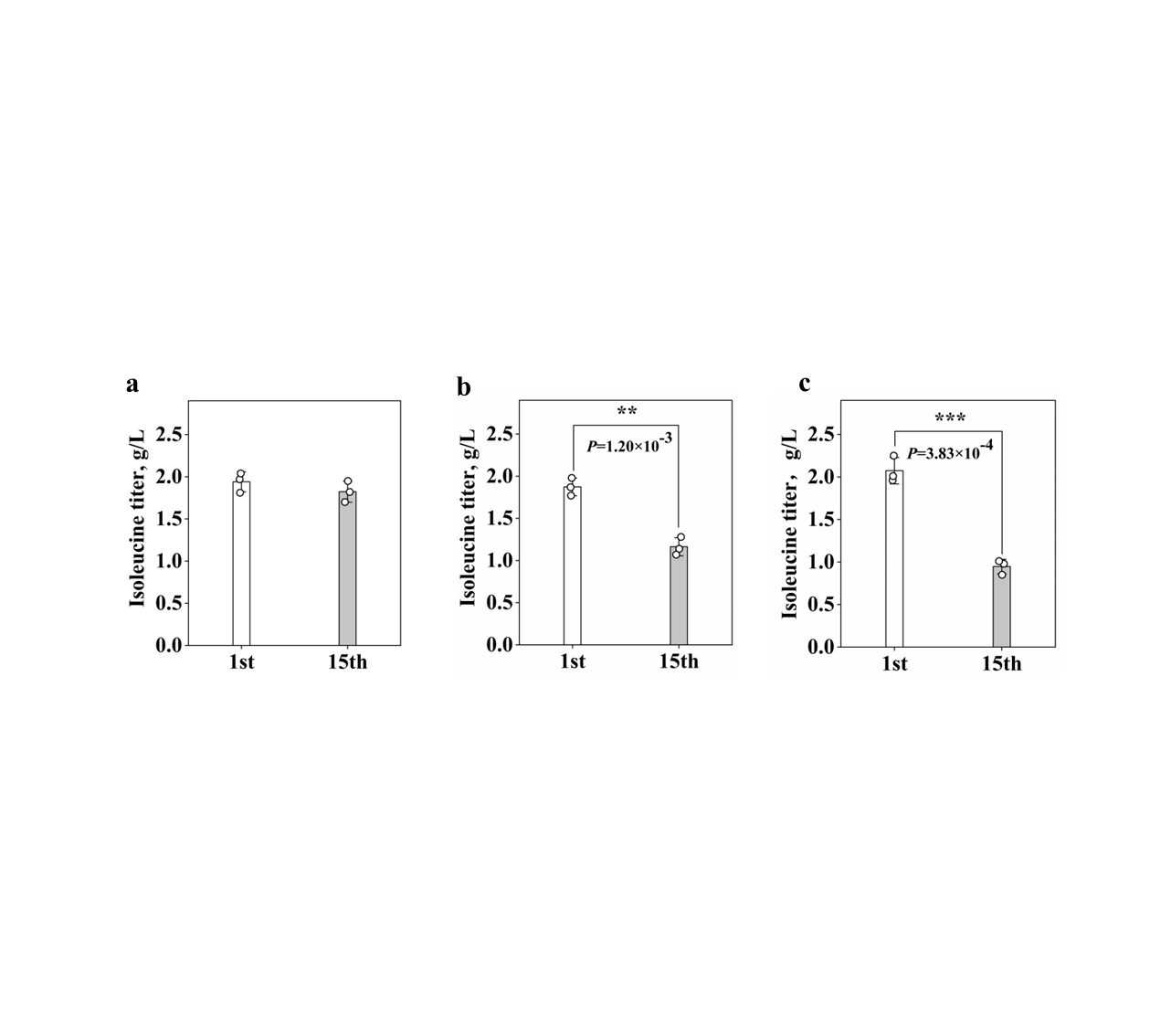


**Fig. S5** Genetic stability evaluation of mutagenic strains. **a** The isoleucine titer of strain cgl-11. **b** The isoleucine titer of strain cgl-22. **c** The isoleucine titer of strain cgl-35. Statistical significance is denoted as ^*^*P* < 0.05, ^**^*P* < 0.01, and ^***^*P* < 0.001. Values and error bars represent the mean values and standard deviations of three biological repeats


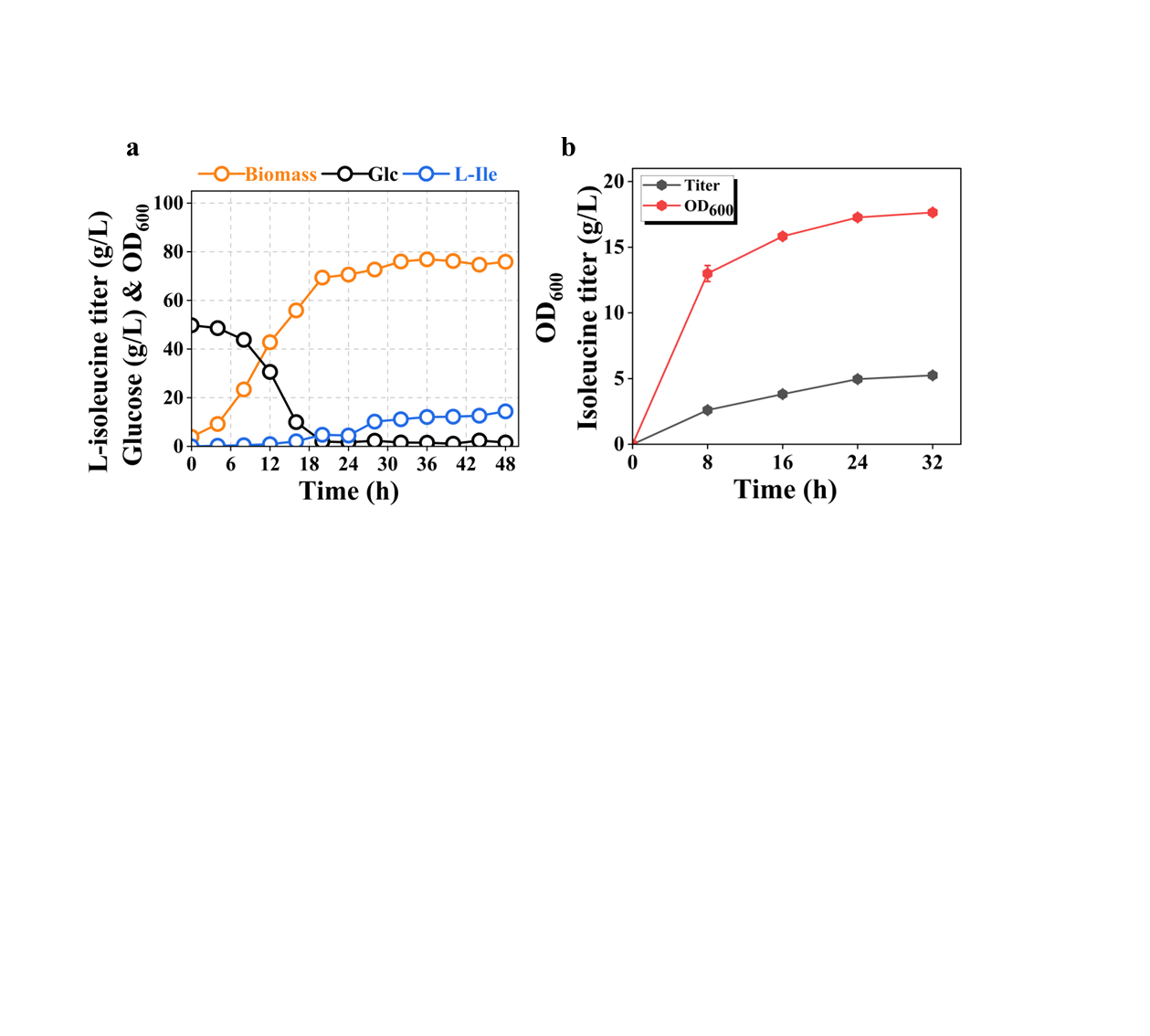


**Fig. S6** Evaluation of the fermentation performance of strain cgl-Ile0. **a** Fermentation curve of strain cgl-Ile0 in a 500-mL shake flask. **b** Fermentation curve of strain cgl-Ile0 in a 5-L fermenter. Values and error bars represent the mean values and standard deviations of three biological repeats


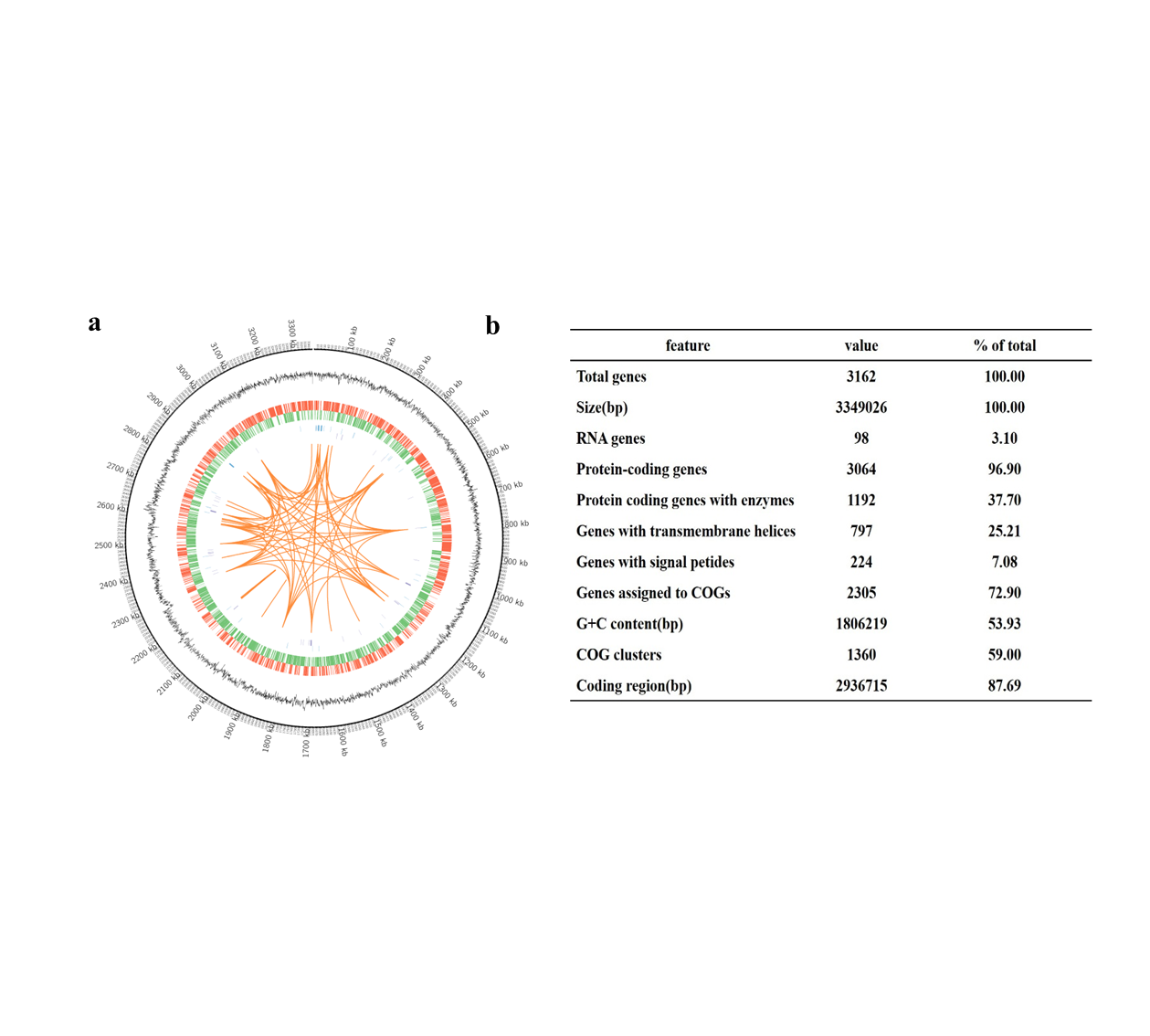


**Fig. S7** Genetic background analysis of strain cgl-Ile0. **a** Circular genome map of strain cgl-Ile0. **b** Genomic features of strain cgl-Ile0


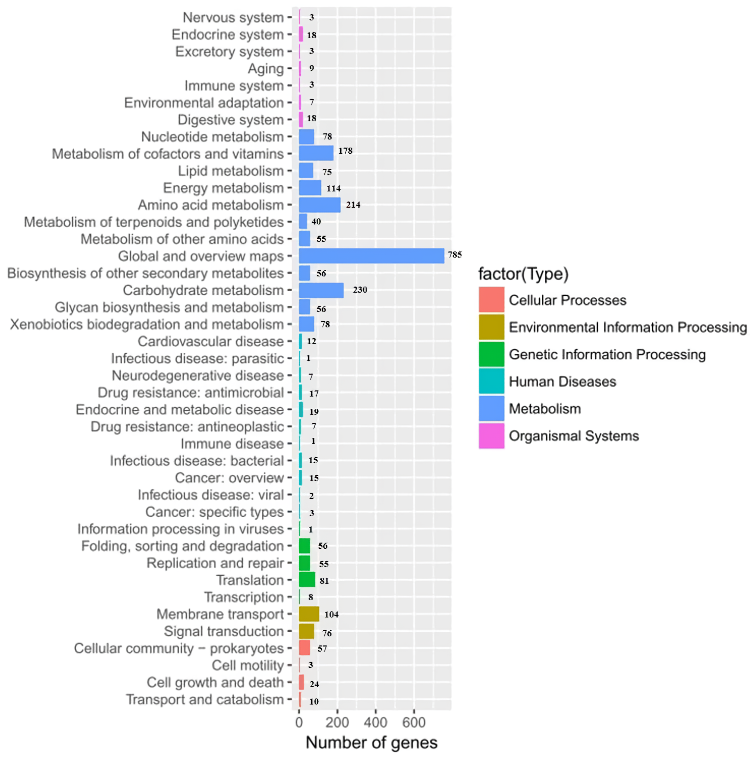


**Fig. S8** Distribution of KEGG functional notes in the mutated genes of strain cgl-Ile0


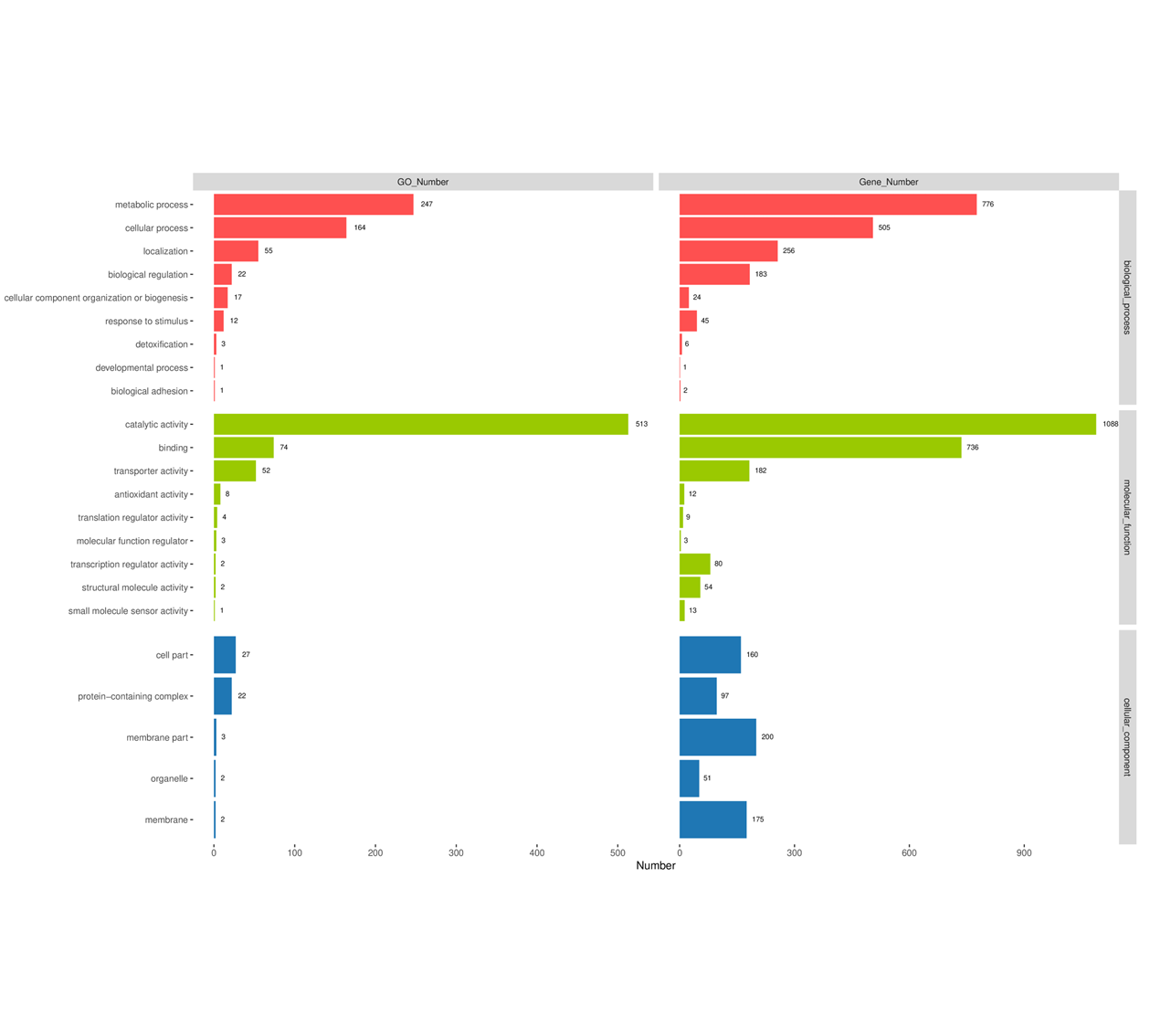


**Fig. S9** Distribution of GO functional notes in the mutated genes of strain cgl-Ile0

**
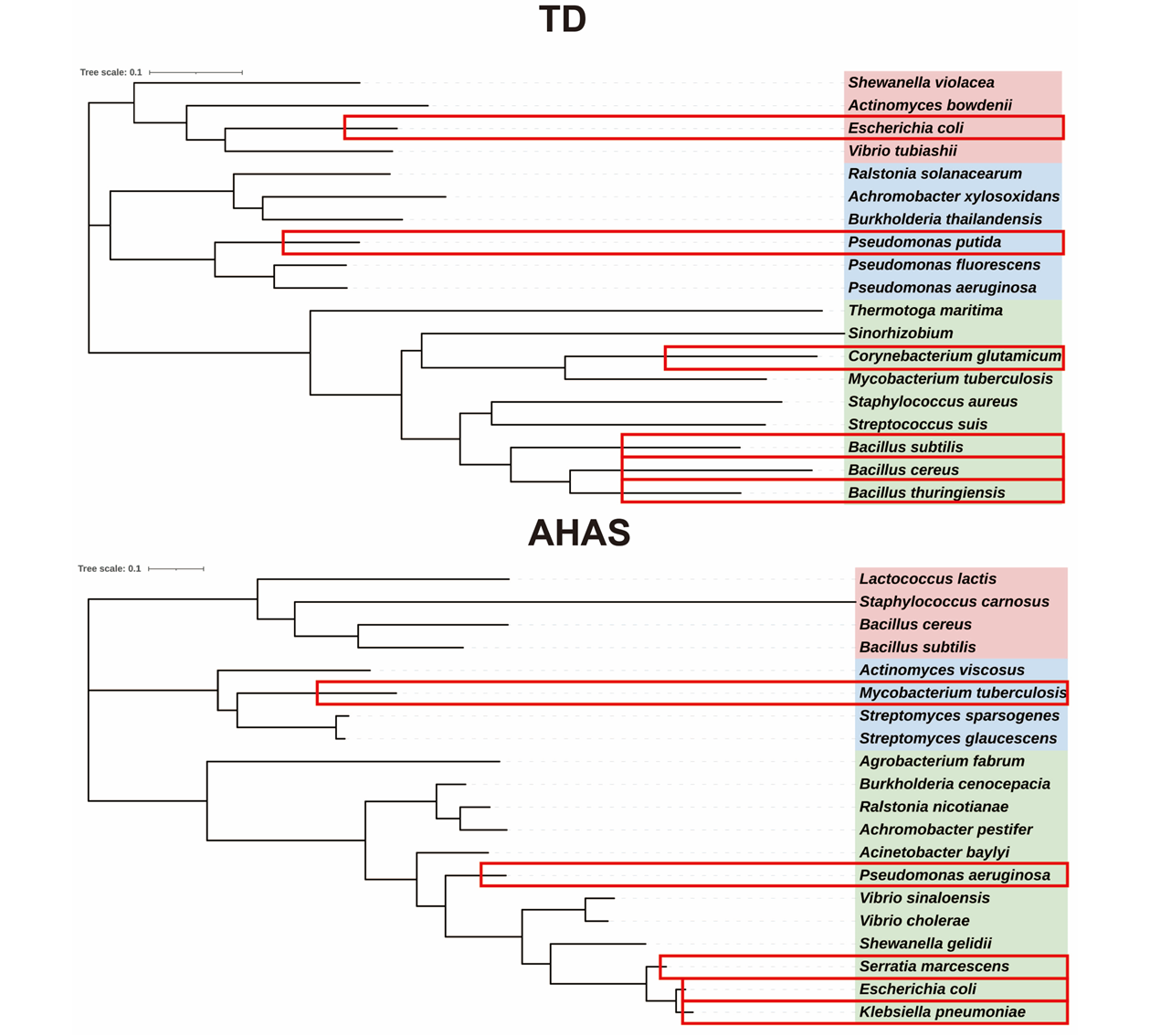
**

**Fig. S10** Evolutionary tree of threonine dehydratase (TD) and acetohydroxyacid synthase (AHAS)


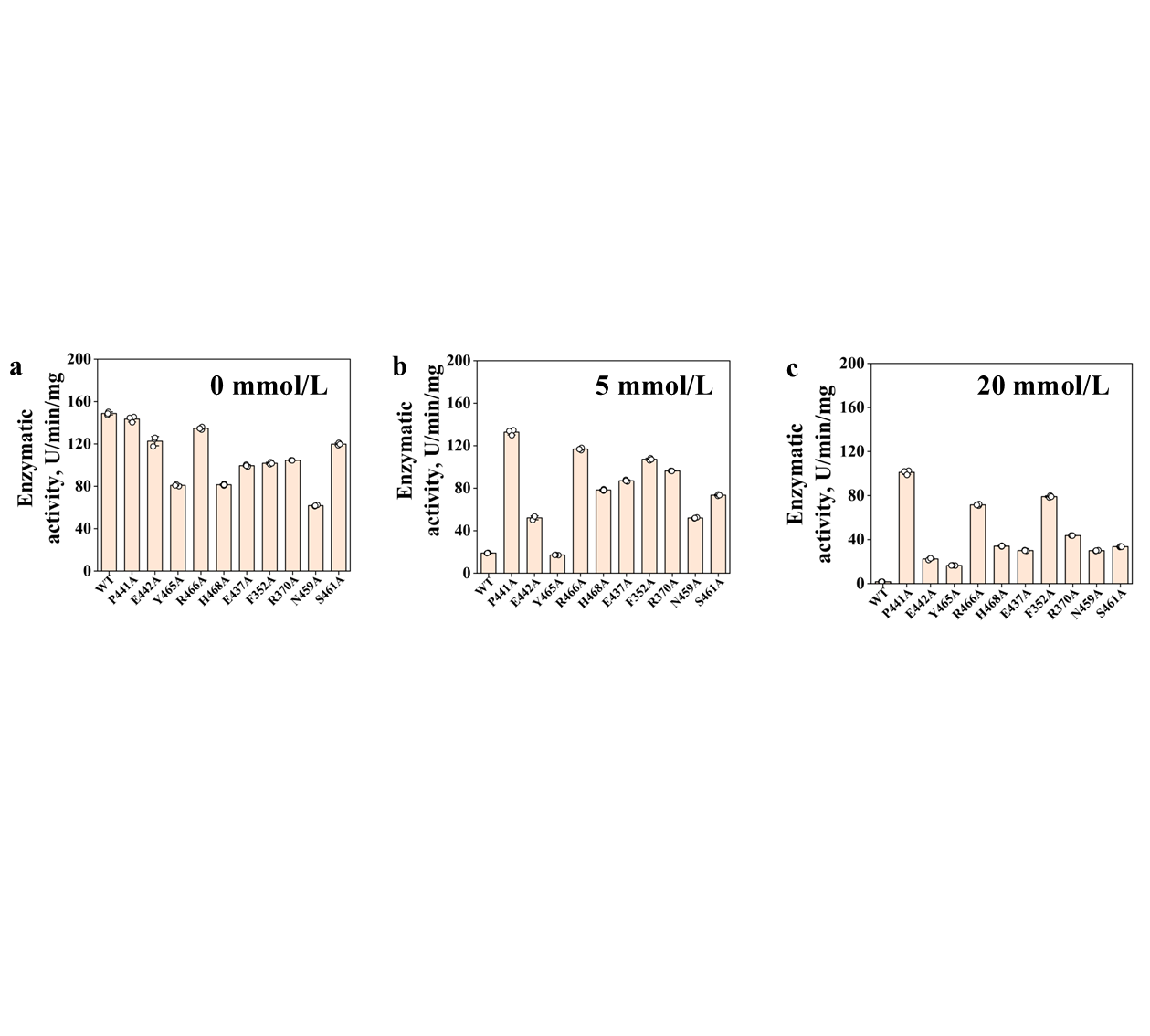


**Fig. S11** Alanine scanning of the key amino acid residues of threonine dehydratase. **a** Enzymatic activity of threonine dehydratase and its mutants under 0 mmol/L isoleucine conditions. **b** Enzymatic activity of threonine dehydratase and its mutants under 5 mmol/L isoleucine conditions. **c** Enzymatic activity of threonine dehydratase and its mutants under 20 mmol/L isoleucine conditions. Values and error bars represent the mean values and standard deviations of three biological repeats


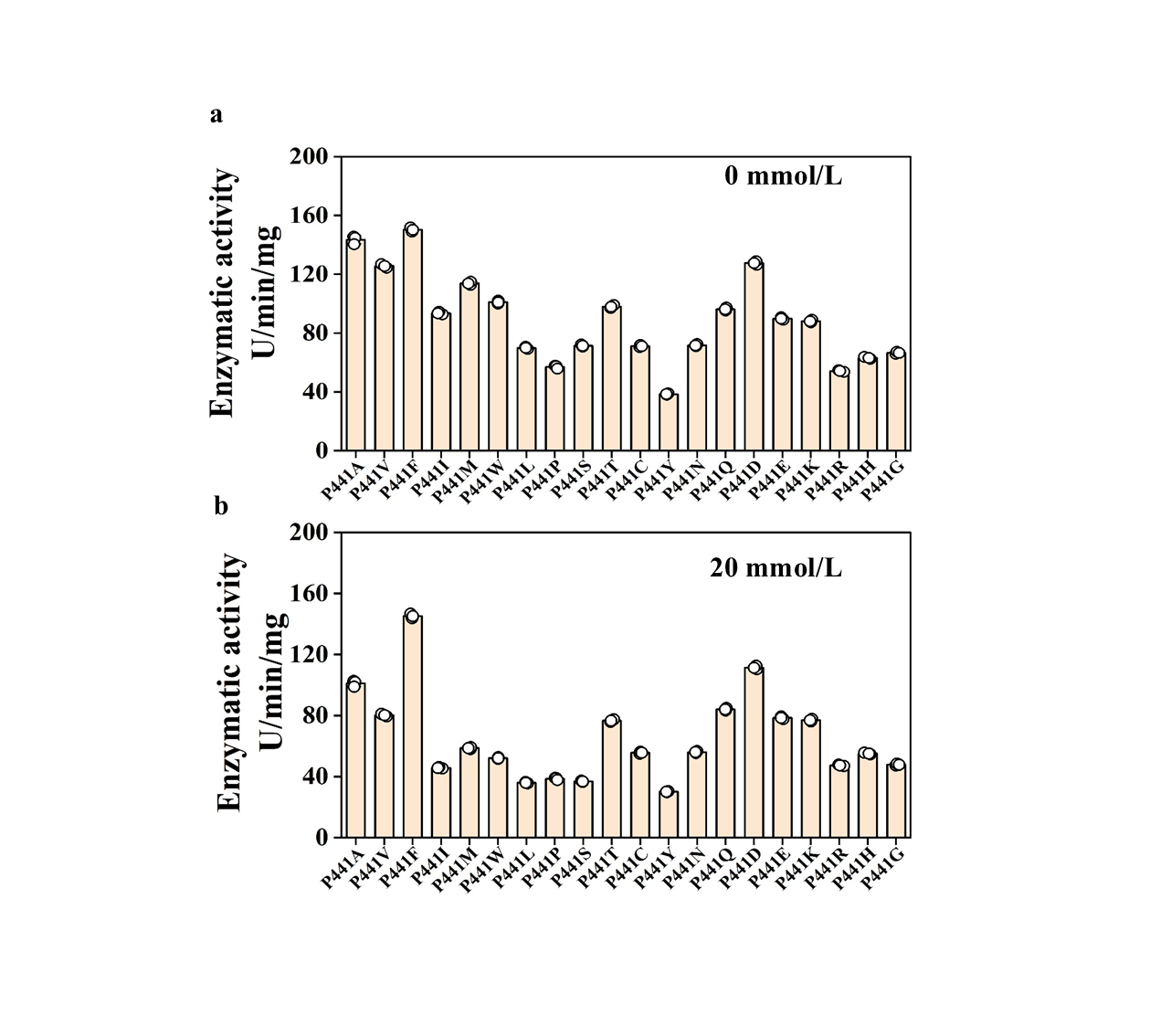


**Fig. S12** Saturated mutations of the key amino acid residues of threonine dehydratase. **a** Enzymatic activity of threonine dehydratase mutants under 0 mmol/L isoleucine conditions. **b** Enzymatic activity of threonine dehydratase mutants under 20 mmol/L isoleucine conditions. Values and error bars represent the mean values and standard deviations of three biological repeats


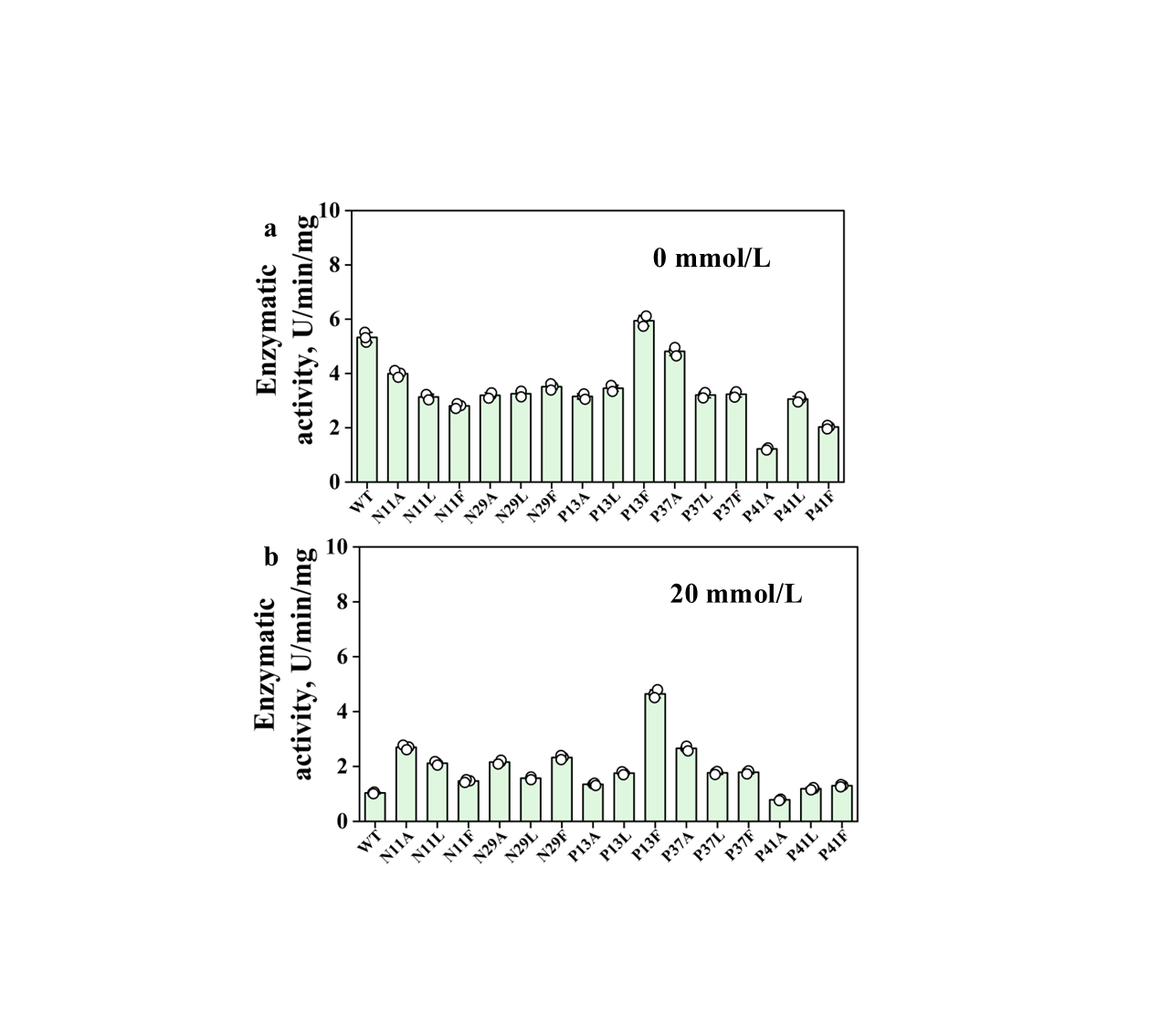


**Fig. S13** ALF scanning of the key amino acid residues of acetyl lactate synthase. **a** Enzymatic activity of acetyl lactate synthase and its mutants under 0 mmol/L isoleucine conditions. **b** Enzymatic activity of acetyl lactate synthase and its mutants under 20 mmol/L isoleucine conditions. Values and error bars represent the mean values and standard deviations of three biological repeats


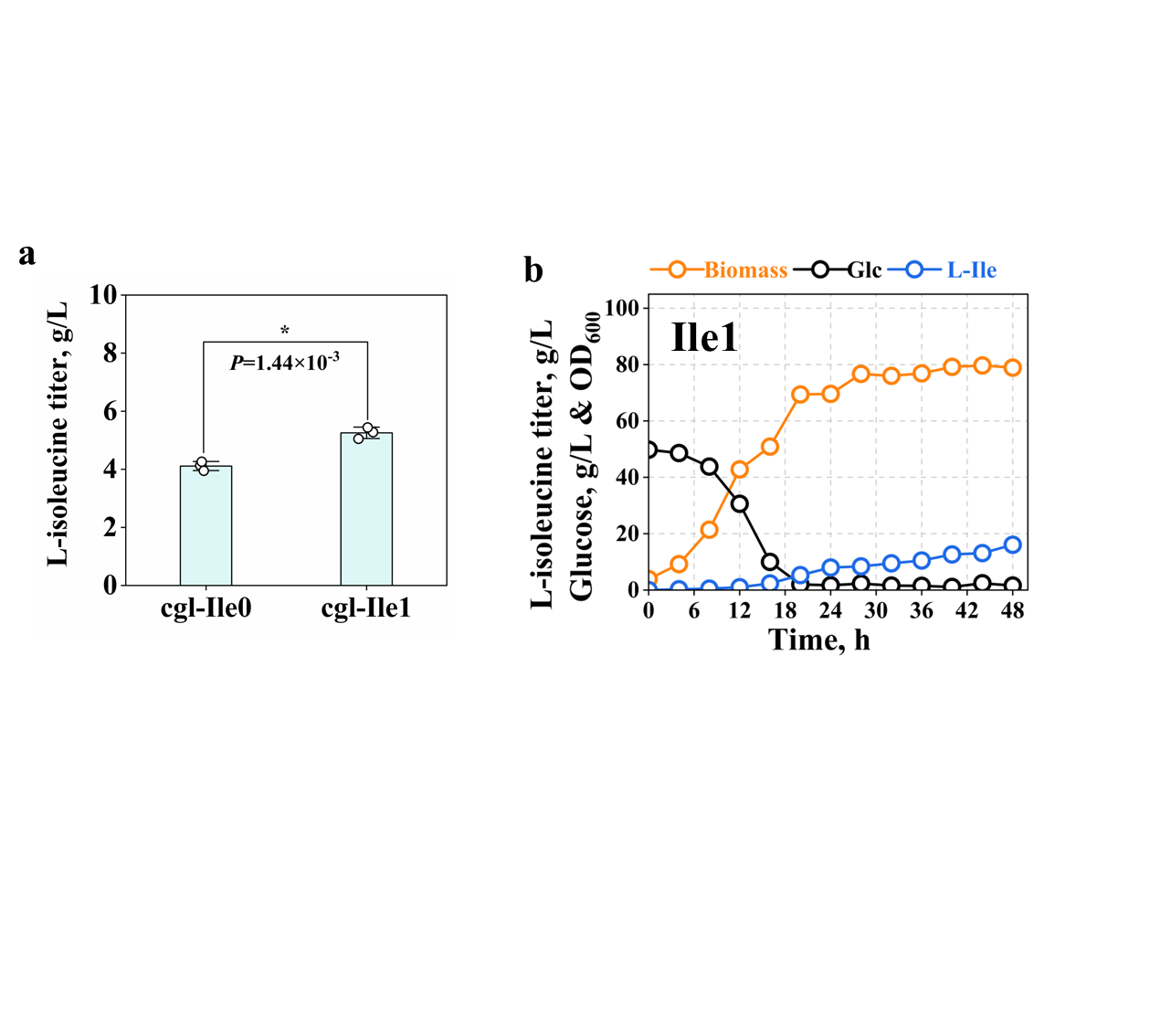


**Fig. S14** Evaluation of the fermentation performance of the strain cgl-Ile1. **a** The isoleucine titer of strains cgl-Ile0 and cgl-Ile1 in 500-mL shake flasks. **b** Fermentation curve of strain cgl-Ile1 in a 5-L fermenter. Statistical significance is denoted as ^*^*P* < 0.05. Values and error bars represent the mean values and standard deviations of three biological repeats


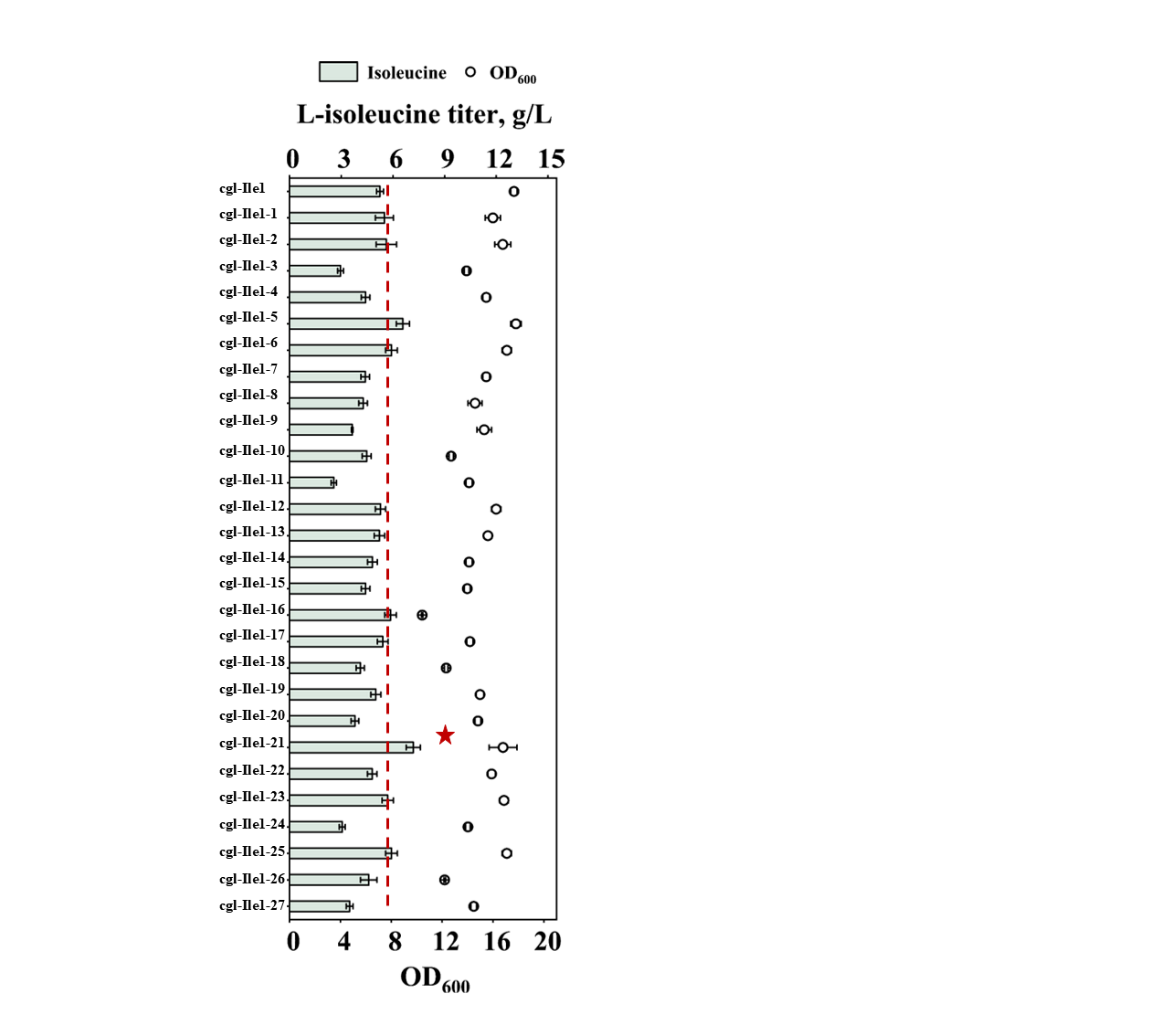


**Fig.** **S15** Evaluation of the fermentation of strain cgl-Ile1-1~27 in a 500-mL shake flask. Values and error bars represent the mean values and standard deviations of three biological repeats. cgl-Ile1, NCgl0008::Ptuf-EcilvAP441Fand NCgl0032::Ptuf-PailvIHP13F based on the strain cgl-Ile0, cgl-Ile1-1, P*_tuf_*-*ilvC*-P*_tuf_*-*ilvD*-P*_tuf_*-*ilvE* based on the strain cgl-Ile1, cgl-Ile1-2, P*_tuf_*-*ilvC*-P*_tuf_*-*ilvD*-P*_gapA_*-*ilvE* based on the strain cgl-Ile1, cgl-Ile1-3, P*_tuf_*-*ilvC*-P*_tuf_*-*ilvD*-P*_sod_*-*ilvE* based on the strain cgl-Ile1, cgl-Ile1-4, P*_tuf_*-*ilvC*-P*_gapA_*-*ilvD*-P*_tuf_*-*ilvE* based on the strain cgl-Ile1, cgl-Ile1-5, P*_tuf_*-*ilvC*-P*_gapA_*-*ilvD*-P*_gapA_*-*ilvE* based on the strain cgl-Ile1, cgl-Ile1-6, P*_tuf_*-*ilvC*-P*_gapA_*-*ilvD*-P*_sod_*-*ilvE* based on the strain cgl-Ile1, cgl-Ile1-7, P*_tuf_*-*ilvC*-P*_sod_*-*ilvD*-P*_tuf_*-*ilvE* based on the strain cgl-Ile1, cgl-Ile1-8, P*_tuf_*-*ilvC*-P*_sod_*-*ilvD*-P*_gapA_*-*ilvE* based on the strain cgl-Ile1, cgl-Ile1-9, P*_tuf_*-*ilvC*-P*_sod_*-*ilvD*-P*_sod_*-*ilvE* based on the strain cgl-Ile1, cgl-Ile1-10, P*_sod_*-*ilvC*-P*_tuf_*-*ilvD*-P*_tuf_*-*ilvE* based on the strain cgl-Ile1, cgl-Ile1-11, P*_sod_*-*ilvC*-P*_tuf_*-*ilvD*-P*_gapA_*-*ilvE* based on the strain cgl-Ile1, cgl-Ile1-12, P*_sod_*-*ilvC*-P*_tuf_*-*ilvD*-P*_sod_*-*ilvE* based on the strain cgl-Ile1, cgl-Ile1-13, P*_sod_*-*ilvC*-P*_gapA_*-*ilvD*-P*_tuf_*-*ilvE* based on the strain cgl-Ile1, cgl-Ile1-14, P*_sod_*-*ilvC*-P*_gapA_*-*ilvD*-P*_gapA_*-*ilvE* based on the strain cgl-Ile1, cgl-Ile1-15, P*_sod_*-*ilvC*-P*_gapA_*-*ilvD*-P*_sod_*-*ilvE* based on the strain cgl-Ile1, cgl-Ile1-16, P*_sod_*-*ilvC*-P*_sod_*-*ilvD*-P*_tuf_*-*ilvE* based on the strain cgl-Ile1, cgl-Ile1-17, P*_sod_*-*ilvC*-P*_sod_*-*ilvD*-P*_gapA_*-*ilvE* based on the strain cgl-Ile1, cgl-Ile1-18, P*_sod_*-*ilvC*-P*_sod_*-*ilvD*-P*_sod_*-*ilvE* based on the strain cgl-Ile1, cgl-Ile1-19, P*_gapA_*-*ilvC*-P*_tuf_*-*ilvD*-P*_tuf_*-*ilvE* based on the strain cgl-Ile1, cgl-Ile1-20, P*_gapA_*-*ilvC*-P*_tuf_*-*ilvD*-P*_gapA_*-*ilvE* based on the strain cgl-Ile1, cgl-Ile1-21, P*_gapA_*-*ilvC*-P*_tuf_*-*ilvD*-P*_sod_*-*ilvE* based on the strain cgl-Ile1, cgl-Ile1-22, P*_gapA_*-*ilvC*-P*_gapA_*-*ilvD*-P*_tuf_*-*ilvE* based on the strain cgl-Ile1, cgl-Ile1-23, P*_gapA_*-*ilvC*-P*_gapA_*-*ilvD*-P*_gapA_*-*ilvE* based on the strain cgl-Ile1, cgl-Ile1-24, P*_gapA_*-*ilvC*-P*_gapA_*-*ilvD*-P*_sod_*-*ilvE* based on the strain cgl-Ile1, cgl-Ile1-25, P*_gapA_*-*ilvC*-P*_sod_*-*ilvD*-P*_tuf_*-*ilvE* based on the strain cgl-Ile1, cgl-Ile1-26, P*_gapA_*-*ilvC*-P*_sod_*-*ilvD*-P*_gapA_*-*ilvE* based on the strain cgl-Ile1, cgl-Ile1-27, P*_gapA_*-*ilvC*-P*_sod_*-*ilvD*-P*_sod_*-*ilvE* based on the strain cgl-Ile1


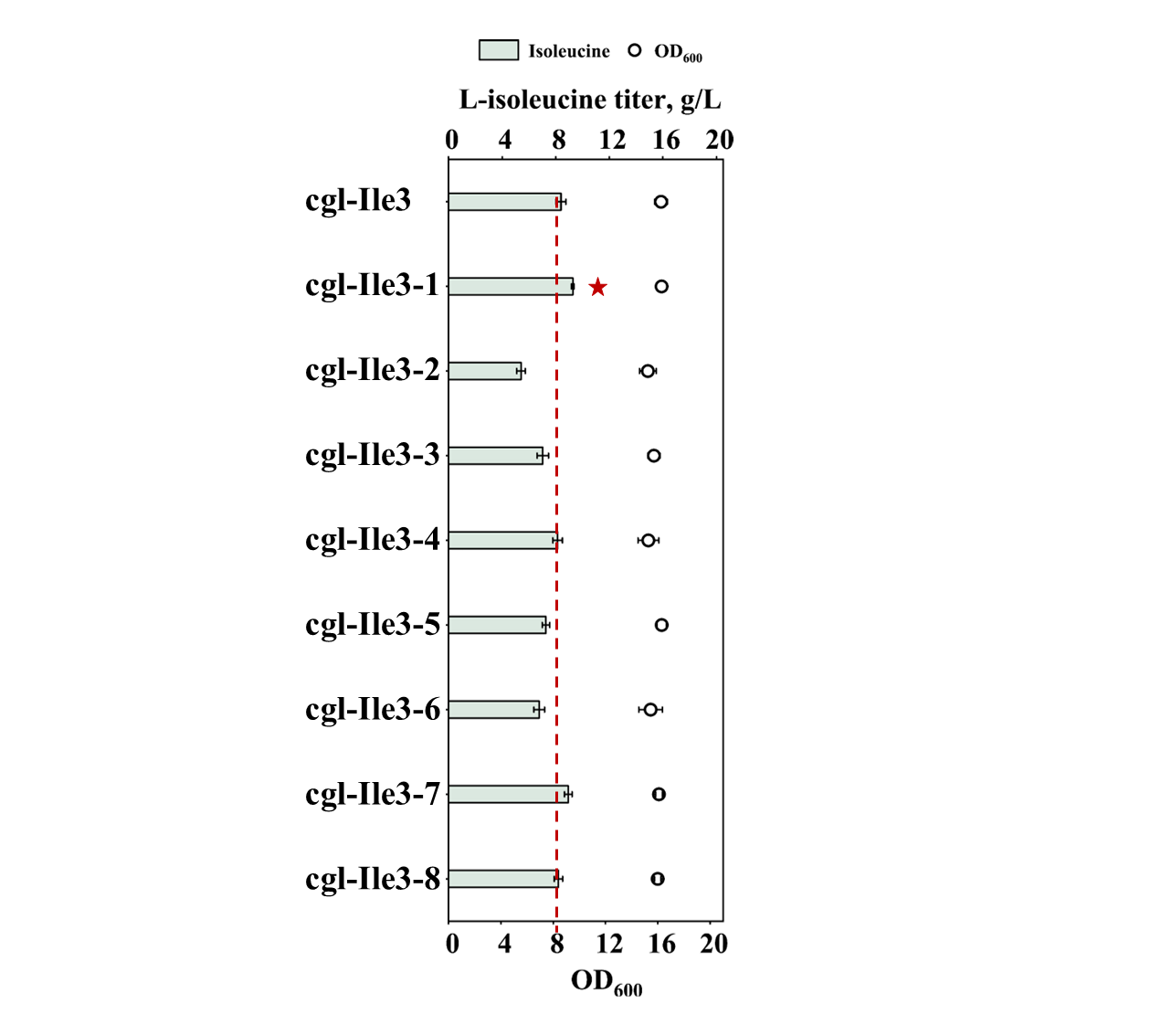


**Fig. S16** Evaluation of the fermentation of strain cgl-Ile3-1~8 in a 500-mL shake flask. Values and error bars represent the mean values and standard deviations of three biological repeats. cgl-Ile3-1, P*_lysC_*-*hom*^G378E^-P*_ncgl0281_*-*lysC^G277A^* based on the strain cgl-Ile3, cgl-Ile3-2, P*_lysC_*-*hom*^G378E^-P*_lysC_*-*lysC^G277A^* based on the strain cgl-Ile3, cgl-Ile3-3, P*_lysC_*-*hom*^G378E^-P*_sod_*-*lysC^G277A^* based on the strain cgl-Ile3, cgl-Ile3-4, P*_ncgl0281_*-*hom*^G378E^-P*_ncgl0281_*-*lysC^G277A^* based on the strain cgl-Ile3, cgl-Ile3-5, P*_ncgl0281_*-*hom*^G378E^-P*_lysC_*-*lysC^G277A^* based on the strain cgl-Ile3, cgl-Ile3-6, P*_ncgl0281_*-*hom*^G378E^-P*_sod_*-*lysC^G277A^* based on the strain cgl-Ile3, cgl-Ile3-7, P*_sod_*-*hom*^G378E^-P*_ncgl0281_*-*lysC^G277A^* based on the strain cgl-Ile3, cgl-Ile3-8, P*_sod_*-*hom*^G378E^-P*_lysC_*-*lysC^G277A^* based on the strain cgl-Ile3


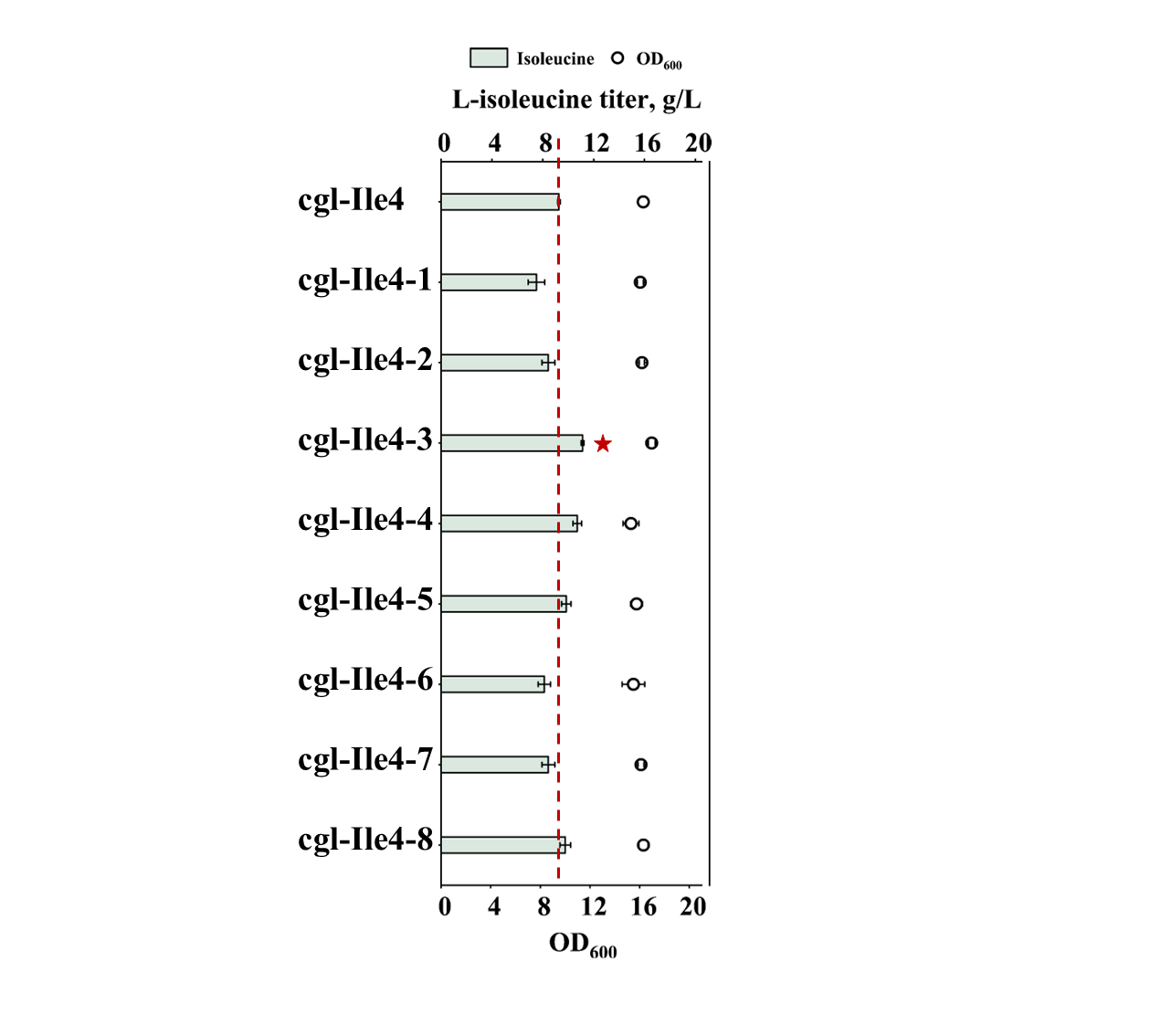


**Fig. S17** Evaluation of the fermentation of strain cgl-Ile4-1~8 in a 500-mL shake flask. Values and error bars represent the mean values and standard deviations of three biological repeats. cgl-Ile4-1, P*_gapA_*-*pyc*-P*_gapA_*-*ppc* based on the strain cgl-Ile4, cgl-Ile4-2, P*_gapA_*-*pyc*-P*_sod_*-*ppc* based on the strain cgl-Ile4, cgl-Ile4-3, P*_gapA_*-*pyc*-P*_lysC_*-*ppc* based on the strain cgl-Ile4, cgl-Ile4-4, P*_sod_*-*pyc*-P*_gapA_*-*ppc* based on the strain cgl-Ile4, cgl-Ile4-5, P*_sod_*-*pyc*-P*_sod_*-*ppc* based on the strain cgl-Ile4, cgl-Ile4-6, P*_sod_*-*pyc*-P*_lysC_*-*ppc* based on the strain cgl-Ile4, cgl-Ile4-7, P*_lysC_*-*pyc*-P*_gapA_*-*ppc* based on the strain cgl-Ile4, cgl-Ile4-8, P*_lysC_*-*pyc*-P*_sod_*-*ppc* based on the strain cgl-Ile4


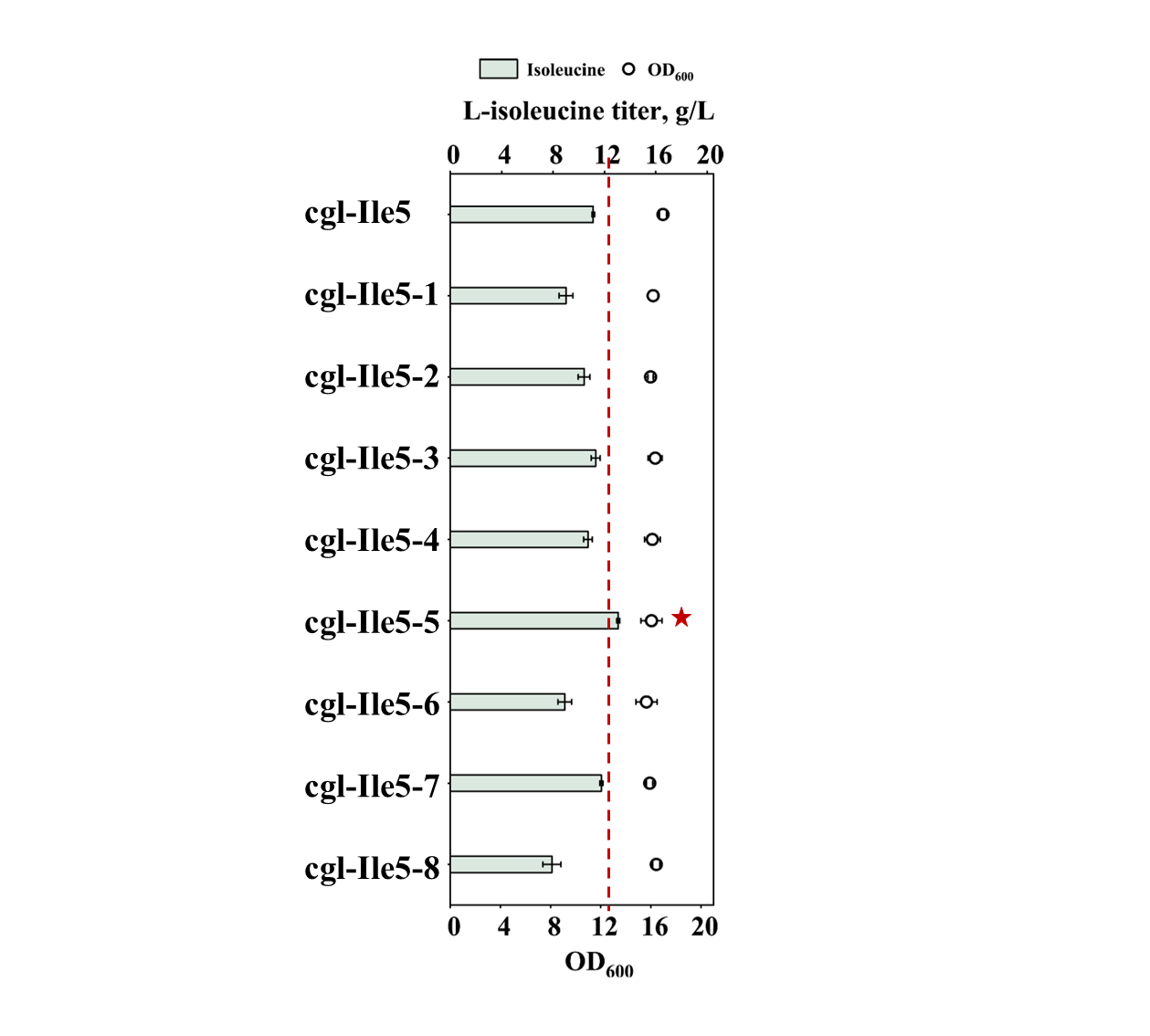


**Fig. S18** Evaluation of the fermentation of strain cgl-Ile5-1~8 in a 500-mL shake flask. Values and error bars represent the mean values and standard deviations of three biological repeats. cgl-Ile5-1, P*_sod_*-*aspB*-P*_sod_*-*gltA* based on the strain cgl-Ile5, cgl-Ile5-2, P*_sod_*-*aspB*-P*_trc_*-*gltA* based on the strain cgl-Ile5, cgl-Ile5-3, P*_sod_*-*aspB*-P*_gapA_*-*gltA* based on the strain cgl-Ile5, cgl-Ile5-4, P*_gapA_*-*aspB*-P*_sod_*-*gltA* based on the strain cgl-Ile5, cgl-Ile5-5, P*_gapA_*-*aspB*-P*_trc_*-*gltA* based on the strain cgl-Ile5, cgl-Ile5-6, P*_gapA_*-*aspB*-P*_gapA_*-*gltA* based on the strain cgl-Ile5, cgl-Ile5-7, P*_trc_*-*aspB*-P*_sod_*-*gltA* based on the strain cgl-Ile5, cgl-Ile5-8, P*_trc_*-*aspB*-P*_gapA_*-*gltA* based on the strain cgl-Ile5


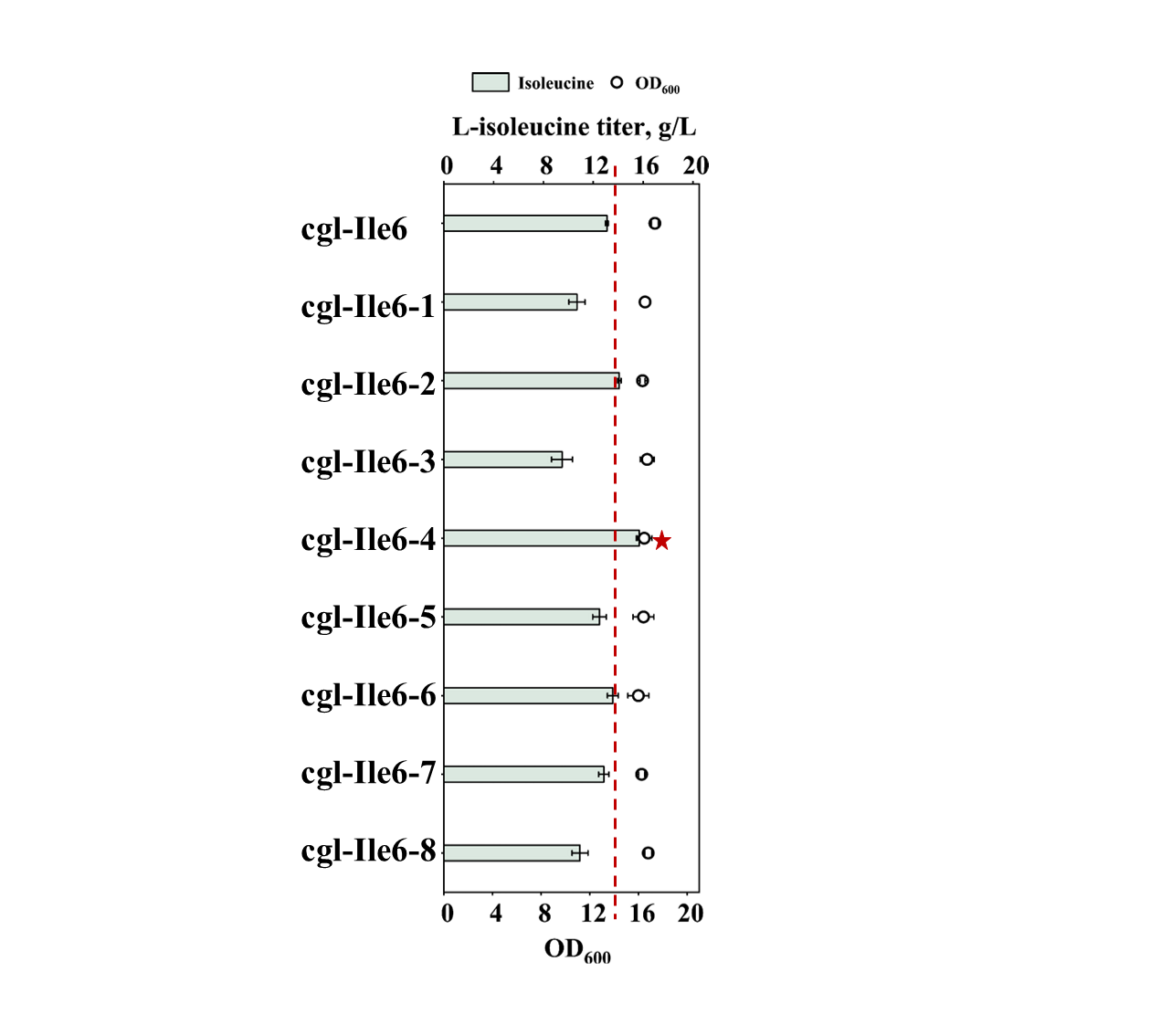


**Fig. S19** Evaluation of the fermentation of strain cgl-Ile6-1~8 in a 500-mL shake flask. Values and error bars represent the mean values and standard deviations of three biological repeats. cgl-Ile6-1, P*_ncgl0281_*-*ppnK* and NCgl0992::P*_lysC_*-*EcpntAB* based on the strain cgl-Ile6, cgl-Ile6-2, P*_ncgl0281_*-*ppnK* and NCgl0992::P*_gapA_*-*EcpntAB* based on the strain cgl-Ile6, cgl-Ile6-3, P*_ncgl0281_*-*ppnK* and NCgl0992::P*_ncgl0281_*-*EcpntAB* based on the strain cgl-Ile6, cgl-Ile6-4, P*_gapA_*-*ppnK* and NCgl0992::P*_lysC_*-*EcpntAB* based on the strain cgl-Ile6, cgl-Ile6-5, P*_gapA_*-*ppnK* and NCgl0992::P*_gapA_*-*EcpntAB* based on the strain cgl-Ile6, cgl-Ile6-6, P*_gapA_*-*ppnK* and NCgl0992::P*_ncgl0281_*-*EcpntAB* based on the strain cgl-Ile6, cgl-Ile6-7, P*_lysC_*-*ppnK* and NCgl0992::P*_gapA_*-*EcpntAB* based on the strain cgl-Ile6, cgl-Ile6-8, P*_lysC_*-*ppnK* and NCgl0992::P*_ncgl0281_*-*EcpntAB* based on the strain cgl-Ile6


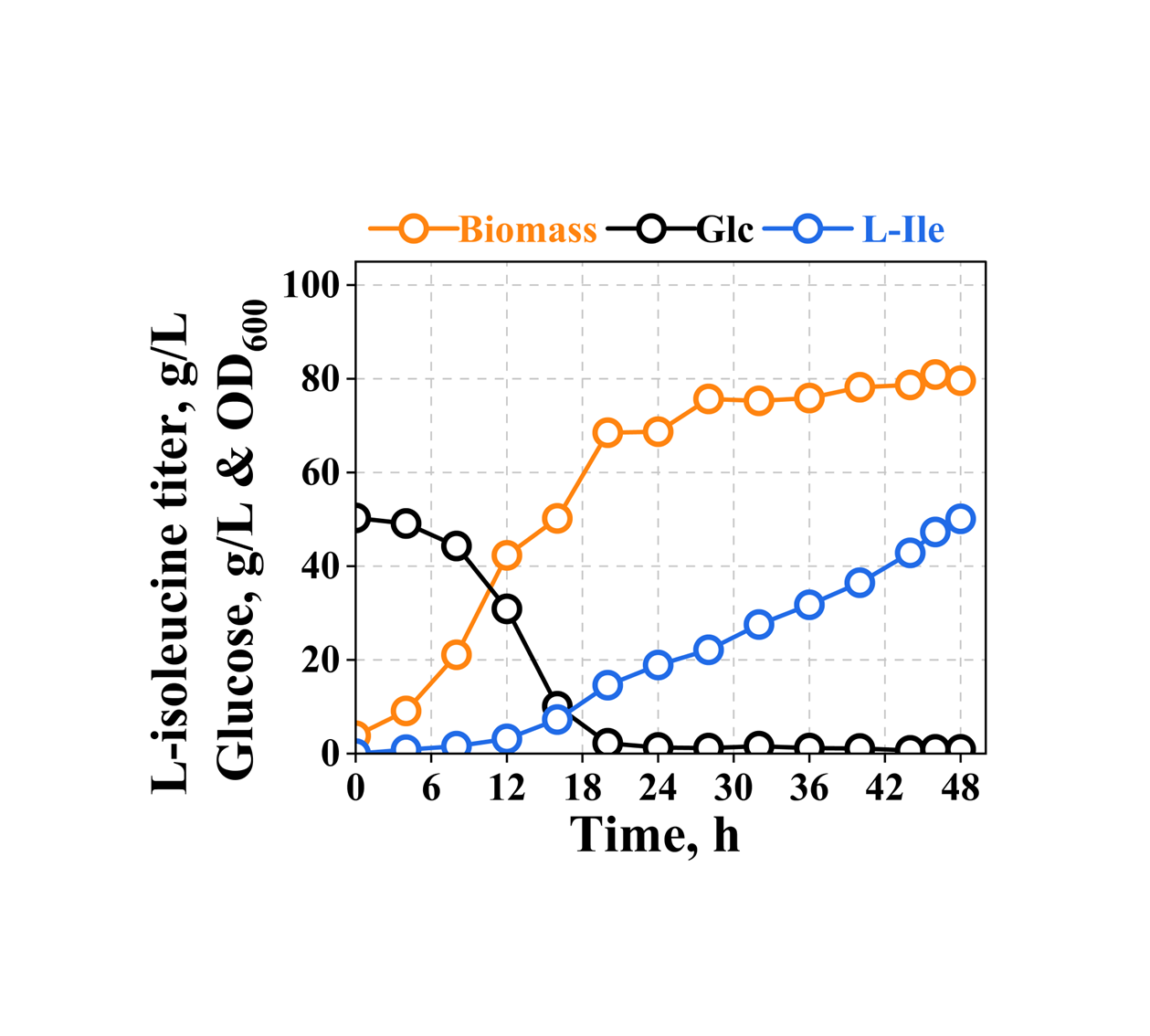


**Fig. S20** Evaluation of the fermentation performance of strain cgl-Ile8 in a 50-L fermenter. Fermentation curve of strain cgl-Ile8 in a 50-L fermenter

# Supplementary tables

# Table S1 Strains & Plasmids used in this study

| **Strain &** **Plasmid** | **Characteristics** |
| --- | --- |
| pK18*mobsacB* | ColE1/pMB1, Km^R^, *sacB*, *oriT*, *lacZ*, RP4 *mob* |
| pECXK99E | ColE1/pMB1, *lacIq*, Km^R^, P_trc_, rrnB T1/T2 terminator, Rep^I325T^ |
| pXMJ19 | ColE1/pMB1, *lacIq*, Cm^R^, P_tac_, rrnB T1/T2 terminator |
| pZK001 | ColE1/pMB1, *lacIq*, Km^R^, P_trc_, rrnB T1/T2 terminator |
| pET-28a(+) | ColE1/pMB1*, lacIq, Km^R^, P_T7_,* T7 terminator |
| DH5α | *E. coli* DH5α (For the preparation of chemically transformed receptive cells) |
| cgl-13032 | *C. glutamicum* ATCC 13032 |
| cgl-Ile0 | *C. glutamicum* ATCC 13032 mutagenic strain |
| cgl-Ile1 | NCgl0008::P*_tuf_*-*EcilvA*^P441F^and NCgl0032::P*_tuf_*-*PailvIH*^P13F^ based on the strain cgl-Ile0 |
| cgl-Ile2 | P*_gapA_*-*ilvC*-P*_tuf_*-*ilvD*-P*_sod_*-*ilvE* based on the strain cgl-Ile1 |
| cgl-Ile3 | NCgl0056:: P*_tuf_*-*EcthrABC* and NCgl0070:: P*_tuf_*-*EcthrABC* based on the strain cgl-Ile2 |
| cgl-Ile4 | P*_lysC_*-*hom*^G378E^-P*_ncgl0281_*-*lysC^G277A^* based on the strain cgl-Ile3 |
| cgl-Ile5 | P*_gapA_*-*pyc*-P*_lysC_*-*ppc* based on the strain cgl-Ile4 |
| cgl-Ile6 | P*_gapA_*-*aspB*-P*_trc_*-*gltA* based on the strain cgl-Ile5 |
| cgl-Ile7 | P*_gapA_*-*ppnK* and NCgl0992::P*_lysC_*-*EcpntAB* based on the strain cgl-Ile6 |
| cgl-Ile8 | NCgl0077::P*_tuf_*-*EcygaZH*and NCgl0092::P*_tuf_*-*EcygaZH* based on the strain cgl-Ile7 |
| cgl-Ile1-1 | P*_tuf_*-*ilvC*-P*_tuf_*-*ilvD*-P*_tuf_*-*ilvE* based on the strain cgl-Ile1 |
| cgl-Ile1-2 | P*_tuf_*-*ilvC*-P*_tuf_*-*ilvD*-P*_gapA_*-*ilvE* based on the strain cgl-Ile1 |
| cgl-Ile1-3 | P*_tuf_*-*ilvC*-P*_tuf_*-*ilvD*-P*_sod_*-*ilvE* based on the strain cgl-Ile1 |
| cgl-Ile1-4 | P*_tuf_*-*ilvC*-P*_gapA_*-*ilvD*-P*_tuf_*-*ilvE* based on the strain cgl-Ile1 |
| cgl-Ile1-5 | P*_tuf_*-*ilvC*-P*_gapA_*-*ilvD*-P*_gapA_*-*ilvE* based on the strain cgl-Ile1 |
| cgl-Ile1-6 | P*_tuf_*-*ilvC*-P*_gapA_*-*ilvD*-P*_sod_*-*ilvE* based on the strain cgl-Ile1 |
| cgl-Ile1-7 | P*_tuf_*-*ilvC*-P*_sod_*-*ilvD*-P*_tuf_*-*ilvE* based on the strain cgl-Ile1 |
| cgl-Ile1-8 | P*_tuf_*-*ilvC*-P*_sod_*-*ilvD*-P*_gapA_*-*ilvE* based on the strain cgl-Ile1 |
| cgl-Ile1-9 | P*_tuf_*-*ilvC*-P*_sod_*-*ilvD*-P*_sod_*-*ilvE* based on the strain cgl-Ile1 |
| cgl-Ile1-10 | P*_sod_*-*ilvC*-P*_tuf_*-*ilvD*-P*_tuf_*-*ilvE* based on the strain cgl-Ile1 |
| cgl-Ile1-11 | P*_sod_*-*ilvC*-P*_tuf_*-*ilvD*-P*_gapA_*-*ilvE* based on the strain cgl-Ile1 |
| cgl-Ile1-12 | P*_sod_*-*ilvC*-P*_tuf_*-*ilvD*-P*_sod_*-*ilvE* based on the strain cgl-Ile1 |
| cgl-Ile1-13 | P*_sod_*-*ilvC*-P*_gapA_*-*ilvD*-P*_tuf_*-*ilvE* based on the strain cgl-Ile1 |
| cgl-Ile1-14 | P*_sod_*-*ilvC*-P*_gapA_*-*ilvD*-P*_gapA_*-*ilvE* based on the strain cgl-Ile1 |
| cgl-Ile1-15 | P*_sod_*-*ilvC*-P*_gapA_*-*ilvD*-P*_sod_*-*ilvE* based on the strain cgl-Ile1 |
| cgl-Ile1-16 | P*_sod_*-*ilvC*-P*_sod_*-*ilvD*-P*_tuf_*-*ilvE* based on the strain cgl-Ile1 |
| cgl-Ile1-17 | P*_sod_*-*ilvC*-P*_sod_*-*ilvD*-P*_gapA_*-*ilvE* based on the strain cgl-Ile1 |
| cgl-Ile1-18 | P*_sod_*-*ilvC*-P*_sod_*-*ilvD*-P*_sod_*-*ilvE* based on the strain cgl-Ile1 |
| cgl-Ile1-19 | P*_gapA_*-*ilvC*-P*_tuf_*-*ilvD*-P*_tuf_*-*ilvE* based on the strain cgl-Ile1 |
| cgl-Ile1-20 | P*_gapA_*-*ilvC*-P*_tuf_*-*ilvD*-P*_gapA_*-*ilvE* based on the strain cgl-Ile1 |
| cgl-Ile1-21 | P*_gapA_*-*ilvC*-P*_tuf_*-*ilvD*-P*_sod_*-*ilvE* based on the strain cgl-Ile1 |
| cgl-Ile1-22 | P*_gapA_*-*ilvC*-P*_gapA_*-*ilvD*-P*_tuf_*-*ilvE* based on the strain cgl-Ile1 |
| cgl-Ile1-23 | P*_gapA_*-*ilvC*-P*_gapA_*-*ilvD*-P*_gapA_*-*ilvE* based on the strain cgl-Ile1 |
| cgl-Ile1-24 | P*_gapA_*-*ilvC*-P*_gapA_*-*ilvD*-P*_sod_*-*ilvE* based on the strain cgl-Ile1 |
| cgl-Ile1-25 | P*_gapA_*-*ilvC*-P*_sod_*-*ilvD*-P*_tuf_*-*ilvE* based on the strain cgl-Ile1 |
| cgl-Ile1-26 | P*_gapA_*-*ilvC*-P*_sod_*-*ilvD*-P*_gapA_*-*ilvE* based on the strain cgl-Ile1 |
| cgl-Ile1-27 | P*_gapA_*-*ilvC*-P*_sod_*-*ilvD*-P*_sod_*-*ilvE* based on the strain cgl-Ile1 |
| cgl-Ile3-1 | P*_lysC_*-*hom*^G378E^-P*_ncgl0281_*-*lysC^G277A^* based on the strain cgl-Ile3 |
| cgl-Ile3-2 | P*_lysC_*-*hom*^G378E^-P*_lysC_*-*lysC^G277A^* based on the strain cgl-Ile3 |
| cgl-Ile3-3 | P*_lysC_*-*hom*^G378E^-P*_sod_*-*lysC^G277A^* based on the strain cgl-Ile3 |
| cgl-Ile3-4 | P*_ncgl0281_*-*hom*^G378E^-P*_ncgl0281_*-*lysC^G277A^* based on the strain cgl-Ile3 |
| cgl-Ile3-5 | P*_ncgl0281_*-*hom*^G378E^-P*_lysC_*-*lysC^G277A^* based on the strain cgl-Ile3 |
| cgl-Ile3-6 | P*_ncgl0281_*-*hom*^G378E^-P*_sod_*-*lysC^G277A^* based on the strain cgl-Ile3 |
| cgl-Ile3-7 | P*_sod_*-*hom*^G378E^-P*_ncgl0281_*-*lysC^G277A^* based on the strain cgl-Ile3 |
| cgl-Ile3-8 | P*_sod_*-*hom*^G378E^-P*_lysC_*-*lysC^G277A^* based on the strain cgl-Ile3 |
| cgl-Ile4-1 | P*_gapA_*-*pyc*-P*_gapA_*-*ppc* based on the strain cgl-Ile4 |
| cgl-Ile4-2 | P*_gapA_*-*pyc*-P*_sod_*-*ppc* based on the strain cgl-Ile4 |
| cgl-Ile4-3 | P*_gapA_*-*pyc*-P*_lysC_*-*ppc* based on the strain cgl-Ile4 |
| cgl-Ile4-4 | P*_sod_*-*pyc*-P*_gapA_*-*ppc* based on the strain cgl-Ile4 |
| cgl-Ile4-5 | P*_sod_*-*pyc*-P*_sod_*-*ppc* based on the strain cgl-Ile4 |
| cgl-Ile4-6 | P*_sod_*-*pyc*-P*_lysC_*-*ppc* based on the strain cgl-Ile4 |
| cgl-Ile4-7 | P*_lysC_*-*pyc*-P*_gapA_*-*ppc* based on the strain cgl-Ile4 |
| cgl-Ile4-8 | P*_lysC_*-*pyc*-P*_sod_*-*ppc* based on the strain cgl-Ile4 |
| cgl-Ile5-1 | P*_sod_*-*aspB*-P*_sod_*-*gltA* based on the strain cgl-Ile5 |
| cgl-Ile5-2 | P*_sod_*-*aspB*-P*_trc_*-*gltA* based on the strain cgl-Ile5 |
| cgl-Ile5-3 | P*_sod_*-*aspB*-P*_gapA_*-*gltA* based on the strain cgl-Ile5 |
| cgl-Ile5-4 | P*_gapA_*-*aspB*-P*_sod_*-*gltA* based on the strain cgl-Ile5 |
| cgl-Ile5-5 | P*_gapA_*-*aspB*-P*_trc_*-*gltA* based on the strain cgl-Ile5 |
| cgl-Ile5-6 | P*_gapA_*-*aspB*-P*_gapA_*-*gltA* based on the strain cgl-Ile5 |
| cgl-Ile5-7 | P*_trc_*-*aspB*-P*_sod_*-*gltA* based on the strain cgl-Ile5 |
| cgl-Ile5-8 | P*_trc_*-*aspB*-P*_gapA_*-*gltA* based on the strain cgl-Ile5 |
| cgl-Ile6-1 | P*_ncgl0281_*-*ppnK* and NCgl0992::P*_lysC_*-*EcpntAB* based on the strain cgl-Ile6 |
| cgl-Ile6-2 | P*_ncgl0281_*-*ppnK* and NCgl0992::P*_gapA_*-*EcpntAB* based on the strain cgl-Ile6 |
| cgl-Ile6-3 | P*_ncgl0281_*-*ppnK* and NCgl0992::P*_ncgl0281_*-*EcpntAB* based on the strain cgl-Ile6 |
| cgl-Ile6-4 | P*_gapA_*-*ppnK* and NCgl0992::P*_lysC_*-*EcpntAB* based on the strain cgl-Ile6 |
| cgl-Ile6-5 | P*_gapA_*-*ppnK* and NCgl0992::P*_gapA_*-*EcpntAB* based on the strain cgl-Ile6 |
| cgl-Ile6-6 | P*_gapA_*-*ppnK* and NCgl0992::P*_ncgl0281_*-*EcpntAB* based on the strain cgl-Ile6 |
| cgl-Ile6-7 | P*_lysC_*-*ppnK* and NCgl0992::P*_gapA_*-*EcpntAB* based on the strain cgl-Ile6 |
| cgl-Ile6-8 | P*_lysC_*-*ppnK* and NCgl0992::P*_ncgl0281_*-*EcpntAB* based on the strain cgl-Ile6 |

##

## Table S2 Genomic integration sites used in this study

| **Genomic integration site** | **Location** | **Length, bp** |
| --- | --- | --- |
| NCgl0008 | 9,471–9,914 | 444 |
| NCgl0032 | 33,462–34,274 | 813 |
| NCgl0056 | 58,087–58,944 | 858 |
| NCgl0070 | 73,844–74,275 | 432 |
| NCgl0992 | 1,087,661–1,088,218 | 558 |
| NCgl0077 | 85,660–86,220 | 561 |

**Table S3** Heterologous genes used in this study

| **Gene** | **Characteristics** | **Source** |
| --- | --- | --- |
| *ilvA*^P441F^ | Mutant of threonine dehydratase | *Escherichia coli* |
| *thrABC* | Fused aspartate kinase/homoserine dehydrogenase,  Homoserine kinase, and  Threonine synthase |  |
| *pntAB* | Pyridine nucleotide transhydrogenase |  |
| *ygaZH* | Branched chain amino acid exporter |  |
| *ilvIH*^P13F^ | Mutant of acetolactate synthase | *Pseudomonas aeruginosa* |

**Table S4** Genetic modification targets used in this study

| **Target** | **Characteristics** | **Size, bp** |
| --- | --- | --- |
| *ilvC* | Ketol-acid reductoisomerase | 1017 |
| *ilvD* | Dihydroxy-acid dehydratase | 1842 |
| *ilvE* | Branched-chain amino acid aminotransferase | 1104 |
| *hom* | Homoserine dehydrogenase | 1338 |
| *lysC* | Aspartate kinase | 1266 |
| *pyc* | Pyruvate carboxylase | 3423 |
| *ppc* | Phosphoenolpyruvate carboxylase | 2760 |
| *aspB* | Aspartate transaminase | 1299 |
| *gltA* | Citrate synthase | 1314 |
| *ppnK* | NAD^+^ kinase | 963 |

**Table S5** Promoters used in this study

| **Promoter** | **Sequence (5^′-^3^′^)** |  |
| --- | --- | --- |
| P*_tuf_* | AGATCGTTTAGATCCGAAGGAAAACGTCGAAAAGCAATTTGCTTTTCGACGCCCCACCCCGCGCGTTTTAGCGTGTCAGTAGGCGCGTAGGGTAAGTGGGGTAGCGGCTTGTTAGATATCTTGAAATCGGCTTTCAACAGATTGATTTCGATGTATTTAGCTGGCCGTTACCCTGCGAATGTCCACAGGGTAGCTGGTAGTTTGAAAATCAACGCCGTTGCCCTTAGGATTCAGTAACTGGCACATTTTGTAATGCGCTAGATCTGTGTGCTCAGTCTTCCAGGCTGCTTATCACAGTGAAAGCAAAACCAATTCGTGGCTGCGAAAGTCGTAGCCACC ACGAAGTCCA GGAGGACATACA |  |
| P*_sod_* | AGAAACAACCAAGGCCAATCCAAATTCGCTGCTGGCCTTCTTTCCACGGACCAGTCACAGGAGTTCCCAACACAGTGTGCGGCGCAGGATTTTCTAAAACAGGATGCCTGCCACCTTTAAGCGCCTCATCAGCGGTAACCATCACGGGTTCGGGTGCGAAAAACCATGCCATAACAGGAATGTTCCTTTCGAAAATTGAGGAAGCCTTATGCCCTTCAACCCTACTTAGCTGCCAATTATTCCGGGCTTGTGACCCGCTACCCGATAAATAGGTCGGCTGAAAAATTTCGTTGCAATATCAACAAAAAGGCCTATCATTGGGAGGTGTCGCACCAAGTACTTTTGCGAAGCGCCATCTGACGGATTTTCAAAAGATGTATATGCTCGGTG CGGAAACCTACGAAAGGATTTTTTACCC |  |
| P*_gapA_* | | ATGATTGAAGCCTAAAAACGACCGAGCCTATTGGGATTACCATTGAAGCCAGTGTGAGTTGCATCACATTGGCTTCAAATCTGAGACTTTAATTTGTGGATTCACGGGGGTGTAATGTAGTTCATAATTAACCCCATTCGGGGGAGCAGATCGTAGTGCGAACGATTTCAGGTTCGTTCCCTGCAAAAACTATTTAGCGCAAGTGTTGGAAATGCCCCCGTTTGGGGTCAATGTCCATTTTTGAATGTGTCTGTATGATTTTGCATCTGCTGCGAAATCTTTGTTTCCCCGCTAAAGTTGAGGACAGGTTGACACGGAGTTGACTCGACGAATTATCCAATGTGAGTAGGTTTGGTGCGTGAGTTGGAAAAATTCGCCATACTCGCCCTTGGGTTCTGTCAGCTCAAGAATTCTTGAGTGACCGATGCTCTGATTGACCTAACTGCTTGACACATTGCATTTCCTACAATCTTTAGAGGAGACACAAC |
| P*_lysC_* | | CCATCTTTTGGGGTGCGGAGCGCGATCCGGTGTCTGACCACGGTGCCCCATGCGATTGTTAATGCCGATGCTAGGGCGAAAAGCACGGCGAGCAGATTGCTTTGCACTTGATTCAGGGTAGTTGACTAAAGAGTTGCTCGCGAAGTAGCACCTGTCACTTTTGTCTCAAATATTAAATCGAATATCAATATATGGTCTGTTTATTGGAACGCGTCCCAGTGGCTGAGACGCATCCGCTAAAGCCCCAGGAACCCTGTGCAGAAAGAAAACACTCCTCTGGCTAGGTAGACACAGTTTATAAAGGTAGAGTTGAGCGGGTAACTGTCAGCACGTAGATCGAAAGGTGCACAAAG |
| P*_ncgl0281_* | | AGTGGCTCCATGTGAACTGGCTGAAAAATAGTTTCGATCTTCAATCATTTGAACATGCATGCATTCTGTGCGTCAAGCGAACGATGTAAGTTTTCAAAATTAATAGTTGACATTTTCAACGTTATGAGTTTTCATTGGTATCACGCCCCGACGAAGTGTCTGGGATCACAAACCTTCAAAGGAGTTTGAA |
| P*_trc_* | TTGACAATTAATCATCCGGCTCGTATAATGCTTAGCGAAAGGATGGGC |  |

**Table S6** Replacement of the promoters of the target genes

| **Regulation** | **Gene** | **Alternative promoter** |
| --- | --- | --- |
| Up-regulation | *ilvC* | P*_sod_*, P*_tuf_*, P*_gapA_* |
|  | *ilvD* | P*_sod_*, P*_tuf_*, P*_gapA_* |
|  | *ilvE* | P*_sod_*, P*_tuf_*, P*_gapA_* |
|  | *hom* | P*_lysC_*, P*_ncgl0281_*, P*_sod_* |
|  | *lysC* | P*_lysC_*, P*_ncgl0281_*, P*_sod_* |
|  | *pyc* | P*_sod_*, P*_lysC_*, P*_gapA_* |
|  | *ppc* | P*_sod_*, P*_lysC_*, P*_gapA_* |
|  | *aspB* | P*_sod_*, P*_trc_*, P*_gapA_* |
|  | *gltA* | P*_sod_*, P*_trc_*, P*_gapA_* |
|  | *ppnK* | P*_lysC_*, P*_ncgl0281_*, P*_gapA_* |

**Table S7** Promoter strength reference

| **Promoter** | **Promoter strength (24 h)** | **error** |
| --- | --- | --- |
| P*_tuf_* | 7715.04 | 334.10 |
| P*_lysC_* | 3320.56 | 148.37 |
| P*_gapA_* | 5770.66 | 249.42 |
| P*_sod_* | 867.24 | 37.97 |
| P*_ncgl0281_* | 4299.65 | 254.43 |
| P*_trc_* | 1497.79 | 66.92 |
| P*_ilvC_* | 1746.73 | 114.29 |
| P*_ilvD_* | 2335.67 | 143.76 |
| P*_ilvE_* | 637.66 | 24.02 |
| P*_hom_* | 2630.95 | 187.90 |
| P*_pyc_* | 3029.44 | 119.91 |
| P*_ppc_* | 2228.69 | 264.99 |
| P*_aspB_* | 1896.30 | 170.88 |
| P*_gltA_* | 1219.74 | 179.58 |
| P*_ppnK_* | 2053.17 | 55.91 |

**Table S8** Primers used in this study

| **Primers** | **Sequences (5’→3’)** |
| --- | --- |
| 99E-Test-F | GGATAACAATTTCACACAGGAAACAGACCAT |
| 99E-Test-R | TCTGCGTTCTGATTTAATCTGTATCAGGCT |
| P*_brnEF_*7-*gfp*-F | CTGGCAACAAAACTGACTATGGGGTATATTGGTAGTGATGGTGAGCAAGGGCGAGGAG |
| 99E-*gfp*-R | AGCTCGGTACCCGGGGATTACTTGTACAGCTCGTCCATGCC |
| P*_brnEF_*7-Lrp-F | CACTACCAATATACCCCATAGTCAGTTTTGTTGCCAGTTTGCGCACCTCAACTAG |
| 99E-Lrp-R | TGCATGCCTGCAGGTCGACTCTAGAGGAAGATGAAAAAGATTCATGCTTCAAAATGCATGCTT |
| Lrp-99E-F | ATTTTGAAGCATGAATCTTTTTCATCTTCCTCTAGAGTCGACCTGCAGGCAT |
| *gfp*-99E-R | AGCTGTACAAGTAATCCCCGGGTACCGAGCTCGAATT |
| XMJ19-Test-F | GCGGATAACAATTTCACACAGGAAACAGAATT |
| XMJ19-Test-R | TGCGTTCTGATTTAATCTGTATCAGGCT |
| Lrp-XMJ19-F | ATTTTGAAGCATGAATCTTTTTCATCTTCCCCGGGTACCGAGCTCGAATTCAG |
| *gfp*-XMJ19-R | ACGAGCTGTACAAGTAATCCTCTAGAGTCGACCTGCAGGCAT |
| XMJ19-*gfp*-R | CAGGTCGACTCTAGAGGATTACTTGTACAGCTCGTCCATGCC |
| XMJ19-Lrp-R | AGCTGAATTCGAGCTCGGTACCCGGGGAAGATGAAAAAGATTCATGCTTCAAAATGCATGCTT |
| ZK001-Test-F | AAGATCTGAAGATTCCTGATACAAATTCTGTTGT |
| ZK001-Test-R | TTATCAGACCGCTTCTGCGTTCTGATTTAAT |
| Lrp-ZK001-F | ATTTTGAAGCATGAATCTTTTTCATCTAGATCTCCATGGCTGTTTTGGCGGAT |
| *gfp*-ZK001-R | ACGAGCTGTACAAGTAACTAGATTTCTTCCAACAAATCTTCCGTCAC |
| ZK001-*gfp*-R | TTGTTGGAAGAAATCTAGTTACTTGTACAGCTCGTCCATGCC |
| ZK001-Lrp-R | TCATCCGCCAAAACAGCCATGGAGATCTAGATGAAAAAGATTCATGCTTCAAAATGCATGCTT |
| 99E-*brnEF*-R | AGCTCGGTACCCGGGGATTGAGGGCATGCGGATTTTTGTTAT |
| *brnEF*-99E-R | AAATCCGCATGCCCTCAATCCCCGGGTACCGAGCTCGAATT |
| *brnEF*-*gfp*-R | GAATCTCTTGCGTTTTTTGCACTTACTTGTACAGCTCGTCCATGCC |
| *gfp*-*brnEF*-F | CATGGACGAGCTGTACAAGTAAGTGCAAAAAACGCAAGAGATTCATTC |
| pBL1-Test-F | GCAGCTTATCGTGTTTTCTTCGTAAAT |
| pBL1-Test-R | TTCCTTTAGCAGCCCTTGCGC |
| 99E-pBL1-F | TTGTTAAACGTGGCAAATAGGCATTAGTGGGTGCGAAGAATAGTCTGCTCATT |
| 99E-pBL1-R | CGTGAAGCTAGATCCCCATCAATCCATTCGGGGTCGTTCACTGGTT |
| pBL1-99E-F | GAACCAGTGAACGACCCCGAATGGATTGATGGGGATCTAGCTTCACG |
| pBL1-99E-R | AGCAGACTATTCTTCGCACCCACTAATGCCTATTTGCCACGTTTAACAAGGT |
| pK18-Test-F | CGTTGTAAAACGACGGCCAGT |
| pK18-Test-R | CCGGCTCGTATGTTGTGTGGAATT |
| pK18-Vec-F | CTAGAGGATCCCCGGGTACCGAGCT |
| pK18-Vec-R | AGTCGACCTGCAGGCATGCAAG |
| Ncgl0008-UP-F | CTTGCATGCCTGCAGGTCGACTGCCGGCCGAGTGATTAGGTAT |
| Ncgl0008-UP-R | TTCGACGTTTTCCTTCGGATCTAAACGATCTGTTCTACATCTTAAGTGAAATGAGAAAAGACCG |
| P*_tuf_*-F | AGATCGTTTAGATCCGAAGGAAAACGTC |
| P*_tuf_*-R | TGTATGTCCTCCTGGACTTCGTGG |
| Ncgl0008-DW-F | GTTCAGGTTCTTTTTGGCGGGTTAGTTAGGTGAGCAACCCTAATAAGAAACCTTTAG |
| Ncgl0008-DW-R | AGCTCGGTACCCGGGGATCCTCTAGGCGTTTGGGATTGGGTGCGGTGC |
| *EcilvA*-F | CCACGAAGTCCAGGAGGACATACAATGGCTGACTCGCAACCCCTGT |
| *EcilvA*-R | TTATTAGGGTTGCTCACCTAACTAACCCGCCAAAAAGAACCTGAAC |
| Ncgl0008-Test-F | TTGGCCTGCGCAGCACGGATTTT |
| Ncgl0008-Test-R | CGCCGTTTAGGCCTACGTTTAGT |
| Ncgl0032-UP-F | CTTGCATGCCTGCAGGTCGACTGGTATAGGCGATGTTGATGGCGAT |
| Ncgl0032-UP-R | TTTTCGACGTTTTCCTTCGGATCTAAACGATCTATTCAACTCTCCTTAAATTGGTCGCGT |
| *PailvIH*-F | CCACGAAGTCCAGGAGGACATACAGTGGAGCTTTTATCTGGCGCTGAAAT |
| *PailvIH*-R | AGCCACGAATTGAAGCGTTAACTCAGATGCTCAGAGTCTTGTCGCC |
| Ncgl0032-DW-F | GACAAGACTCTGAGCATCTGAGTTAACGCTTCAATTCGTGGCTGAGT |
| Ncgl0032-DW-R | AGCTCGGTACCCGGGGATCCTCTAGGTTCGTTGGTTTTGCCGCCT |
| Ncgl0032-Test-F | CCTCCAAGGTGTCCCCACAGAG |
| Ncgl0032-Test-R | CATCGGCAACTGGATTTTTGCGGT |
| *ilvC*-Test-F | AAGGTTTCCCCATTAAGATCGCACT |
| *ilvC*-Test-R | ACAATTAGTCCGGAGGGCATTTCCT |
| *ilvC*-UP-F | CTTGCATGCCTGCAGGTCGACTTGGGCATGGTTCGCCAATGGC |
| P*_tuf_*-*ilvC*-UP-R | TTCGACGTTTTCCTTCGGATCTAAACGATCTACCACACACATGTAATTAGACGGTGT |
| P*_tuf_*-*ilvC*-DW-F | CCACGAAGTCCAGGAGGACATACAATGGCTATTGAACTGCTTTATGATGC |
| *ilvC*-DW-R | AGCTCGGTACCCGGGGATCCTCTAGGGTTTCTGCGCGAGCGTCAAC |
| P*_sod_*-F | AGAAACAACCAAGGCCAATCCAAATT |
| P*_sod_*-R | GGGTAAAAAATCCTTTCGTAGGTTTCCGC |
| P*_sod_*-*ilvC*-DW-F | GCGGAAACCTACGAAAGGATTTTTTACCCATGGCTATTGAACTGCTTTATGATGC |
| P*_sod_*-*ilvC*-UP-R | GATTGGCCTTGGTTGTTTCTACCACACACATGTAATTAGACGGTGT |
| P*_gapA_*-F | ATGATTGAAGCCTAAAAACGACCGAG |
| P*_gapA_*-R | GTTGTGTCTCCTCTAAAGATTGTAGG |
| P*_gapA_*-*ilvC*-DW-F | TTCCTACAATCTTTAGAGGAGACACAACATGGCTATTGAACTGCTTTATGATGC |
| P*_gapA_*-*ilvC*-UP-R | CGGTCGTTTTTAGGCTTCAATCATACCACACACATGTAATTAGACGGTGT |
| *ilvD*-Test-F | GAGAATTGGCAAAACTCCAGTGGAT |
| *ilvD*-Test-R | CAGGCCACCGCGGTTGAGCTC |
| *ilvD*-UP-F | CTTGCATGCCTGCAGGTCGACTCTCAACTGCGGTGACCACCT |
| P*_tuf_*-*ilvD*-UP-R | TTCGACGTTTTCCTTCGGATCTAAACGATCTAGCTTCTACTCGTGCAGAGGTGT |
| P*_tuf_*-*ilvD*-DW-F | CCACGAAGTCCAGGAGGACATACAATGATCCCACTTCGTTCAAAAGTCACC |
| *ilvD*-DW-R | AGCTCGGTACCCGGGGATCCTCTAGGCCACCGGCGCGGTGGACGT |
| P*_sod_*-*ilvD*-DW-F | GCGGAAACCTACGAAAGGATTTTTTACCCATGATCCCACTTCGTTCAAAAGTCACC |
| P*_sod_*-*ilvD*-UP-R | CAGCGAATTTGGATTGGCCTTGGTTGTTTCTAGCTTCTACTCGTGCAGAGGTGT |
| P*_gapA_*-*ilvD*-DW-F | TTCCTACAATCTTTAGAGGAGACACAACATGATCCCACTTCGTTCAAAAGTCACC |
| P*_gapA_*-*ilvD*-UP-R | CGGTCGTTTTTAGGCTTCAATCATAGCTTCTACTCGTGCAGAGGTGT |
| *ilvE*-Test-F | GGATGGCATGTTCTCAGCCATT |
| *ilvE*-Test-R | GGTTTCACGAAGCTTCATCGTGATTTCT |
| *ilvE*-UP-F | CTTGCATGCCTGCAGGTCGACTCAAACGAGCTGCAGCGATAATG |
| P*_tuf_*-*ilvE*-UP-R | TTCGACGTTTTCCTTCGGATCTAAACGATCTGGCTAGGCTTGTTAAAAGTTAGTTTCAATTTG |
| P*_tuf_*-*ilvE*-DW-F | CCACGAAGTCCAGGAGGACATACAATGACGTCATTAGAGTTCACAGTAACCC |
| *ilvE*-DW-R | AGCTCGGTACCCGGGGATCCTCTAGTTGTTCACTTCGAAGGTGCCGT |
| P*_sod_*-*ilvE*-DW-F | GCGGAAACCTACGAAAGGATTTTTTACCCATGACGTCATTAGAGTTCACAGTAACCC |
| P*_sod_*-*ilvE*-UP-R | CAGCGAATTTGGATTGGCCTTGGTTGTTTCTGGCTAGGCTTGTTAAAAGTTAGTTTCAATTTG |
| P*_gapA_*-*ilvE*-DW-F | TTCCTACAATCTTTAGAGGAGACACAACATGACGTCATTAGAGTTCACAGTAACCC |
| P*_gapA_*-*ilvE*-UP-R | CGGTCGTTTTTAGGCTTCAATCATGGCTAGGCTTGTTAAAAGTTAGTTTCAATTTG |
| Ncgl0056-Test-F | CCCTCCAAAACAGCCACGGT |
| Ncgl0056-Test-R | TGGCTGTAGGAGATGTGGCGGT |
| Ncgl0056-UP-F | CTTGCATGCCTGCAGGTCGACTCAGCTTTTGTTGTGCCTGACCT |
| Ncgl0056-UP-R | TTCGACGTTTTCCTTCGGATCTAAACGATCTTAGTGCAAAGGTATCAGTGCACCTAGT |
| *EcthrABC*-F | CCACGAAGTCCAGGAGGACATACAATGCGAGTGTTGAAGTTCGGCGGT |
| *EcthrABC*-R | TTACTGATGATTCATCATCAATTTACGCAACGC |
| Ncgl0056-DW-F | GTTGCGTAAATTGATGATGAATCATCAGTAACTGGTCATAGACCTGCTGTTTTCCTATAAGG |
| Ncgl0056-DW-R | AGCTCGGTACCCGGGGATCCTCTAGCGATGGAGGTCAGGACGAAGGAC |
| Ncgl0070-Test-F | CCGGGCGACCAGAACATCTATATT |
| Ncgl0070-Test-R | GGTAGTTTCCGACGATCATCGCATT |
| Ncgl0070-UP-F | CTTGCATGCCTGCAGGTCGACTGTCCACCTCGGTCAGCATCTTT |
| Ncgl0070-UP-R | TTCGACGTTTTCCTTCGGATCTAAACGATCTTAAAGTTCCTCCAAAAGAGATTGTTGATTCG |
| Ncgl0070-DW-F | GTTGCGTAAATTGATGATGAATCATCAGTAAGCTTGCTTGAACGGCGCTTGTT |
| Ncgl0070-DW-R | AGCTCGGTACCCGGGGATCCTCTAGGCTTGCTCGGAACCCTTGTCG |
| *hom*-Test-F | TTGCTCGACTTCACGTGTGCT |
| *hom*-Test-R | TTGTCTGGAATTGGCTCAGTGGACAG |
| *hom*-UP-F | CTTGCATGCCTGCAGGTCGACTCAACTTCTCATGAGGCGACATCAGT |
| P*_sod_*-*hom*-UP-R | CAGCGAATTTGGATTGGCCTTGGTTGTTTCTACCTAGCCACTATAGACCAAGCTGTT |
| P*_sod_*-*hom*-F | GCGGAAACCTACGAAAGGATTTTTTACCCATGACCTCAGCATCTGCCCCAAG |
| *hom*-R | CAGTTCAATTGCCATGTCAGTAAAATTAGTCCCTTTCGAGGCGGATCAC |
| *hom*-DW-F | TCCGCCTCGAAAGGGACTAATTTTACTGACATGGCAATTGAACTGAACG |
| *hom*-DW-R | AGCTCGGTACCCGGGGATCCTCTAGCGCGTAGCCACGGTTGCGCAG |
| P*_ncgl0281_*-F | AGTGGCTCCATGTGAACTGGCT |
| P*_ncgl0281_*-R | TTCAAACTCCTTTGAAGGTTTGTGATCCCAG |
| P*_ncgl0281_*-*hom*-F | GGGATCACAAACCTTCAAAGGAGTTTGAAATGACCTCAGCATCTGCCCCAAG |
| P*_ncgl0281_*-*hom*-UP-R | TTTTCAGCCAGTTCACATGGAGCCACTACCTAGCCACTATAGACCAAGCTGTT |
| P*_lysC_*-F | CCATCTTTTGGGGTGCGGAGC |
| P*_lysC_*-R | CTTTGTGCACCTTTCGATCTACGTG |
| P*_lysC_*-*hom*-F | TCAGCACGTAGATCGAAAGGTGCACAAAGATGACCTCAGCATCTGCCCCAAG |
| P*_lysC_*-*hom*-UP-R | GATCGCGCTCCGCACCCCAAAAGATGGACCTAGCCACTATAGACCAAGCTGTT |
| *lysC*-Test-F | CTTGTACGAGACAGTGCAATGGT |
| *lysC*-Test-R | GAATGGCAGCACGTTGTAAGCGAT |
| *lysC*-UP-F | CTTGCATGCCTGCAGGTCGACTTCATCACCAGTAGTGCGATGCC |
| P*_sod_*-*lysC*-UP-R | CAGCGAATTTGGATTGGCCTTGGTTGTTTCTCTGTGTCTACCTAGCCAGAGGAGT |
| P*_sod_*-*lysC*-F | GCGGAAACCTACGAAAGGATTTTTTACCCGTGGCCCTGGTCGTACAGAAATAT |
| *lysC*-R | CGTTCTGCAGAACCATGTCAATGTTGATTTCTGCATCAGCCAACG |
| *lysC*-DW-F | TTGGCTGATGCAGAAATCAACATTGACATGGTTCTGCAGAACGTCTCTT |
| *lysC*-DW-R | AGCTCGGTACCCGGGGATCCTCTAGCGCCTGCGTCAGCAGCCTGT |
| P*_ncgl0281_*-*lysC*-F | GGGATCACAAACCTTCAAAGGAGTTTGAAGTGGCCCTGGTCGTACAGAAATAT |
| P*_ncgl0281_*-*lysC*-UP-R | TTTTCAGCCAGTTCACATGGAGCCACTCTGTGTCTACCTAGCCAGAGGAGT |
| P*_lysC_*-*lysC*-F | TCAGCACGTAGATCGAAAGGTGCACAAAGGTGGCCCTGGTCGTACAGAAATAT |
| *pyc*-Test-F | GCGGAGTATCCCTGGAAAAGCAT |
| *pyc*-Test-R | TGCACCGTGGGTCTTGATCTTATCTT |
| *pyc*-UP-F | CTTGCATGCCTGCAGGTCGACTAGTCTGTCACCCGCACCGAAG |
| P*_gapA_*-*pyc*-UP-R | CGGTCGTTTTTAGGCTTCAATCATGATTCCCCCAATCAAACATCGGTTTTT |
| P*_gapA_*-*pyc*-DW-F | TTCCTACAATCTTTAGAGGAGACACAACATGTCGACTCACACATCTTCAACGCT |
| *pyc*-DW-R | AGCTCGGTACCCGGGGATCCTCTAGCCTTCAAGGTTGCACCAGCAG |
| P*_lysC_*-*pyc*-DW-F | TCAGCACGTAGATCGAAAGGTGCACAAAGATGTCGACTCACACATCTTCAACGCT |
| P*_lysC_*-*pyc*-UP-R | GATCGCGCTCCGCACCCCAAAAGATGGGATTCCCCCAATCAAACATCGGTTTTT |
| P*sod*-*pyc*-DW-F | GCGGAAACCTACGAAAGGATTTTTTACCCATGTCGACTCACACATCTTCAACGCT |
| P*sod*-*pyc*-UP-R | CAGCGAATTTGGATTGGCCTTGGTTGTTTCTGATTCCCCCAATCAAACATCGGTTTTT |
| *ppc*-Test-F | GGCGCATCCCTCGAGTACCTT |
| *ppc*-Test-R | CCGTCGTCGCGAGGATACGT |
| *ppc*-UP-F | CTTGCATGCCTGCAGGTCGACTCCCAGGCGTTGCAATTCTCG |
| P*_gapA_*-*ppc*-UP-R | CGGTCGTTTTTAGGCTTCAATCATCAGCAAAACAGGTGTTTAGCTGG |
| P*_gapA_*-*ppc*-DW-F | TTCCTACAATCTTTAGAGGAGACACAACATGACTGATTTTTTACGCGATGACATCAG |
| *ppc*-DW-R | AGCTCGGTACCCGGGGATCCTCTAGATGGACGGCGCGTCGATAAGG |
| P*_lysC_*-*ppc*-DW-F | TCAGCACGTAGATCGAAAGGTGCACAAAGATGACTGATTTTTTACGCGATGACATCAG |
| P*_lysC_*-*ppc*-UP-R | GATCGCGCTCCGCACCCCAAAAGATGGCAGCAAAACAGGTGTTTAGCTGGG |
| P*_sod_*-*ppc*-DW-F | GCGGAAACCTACGAAAGGATTTTTTACCCATGACTGATTTTTTACGCGATGACATCAG |
| P*_sod_*-*ppc*-UP-R | CAGCGAATTTGGATTGGCCTTGGTTGTTTCTCAGCAAAACAGGTGTTTAGCTGG |
| *aspB*-Test-F | GCCAGGATTGGGCCTGGT |
| *aspB*-Test-R | TTGAACTTCGGAGCCAACGAC |
| *aspB*-UP-F | CTTGCATGCCTGCAGGTCGACTTTCCCAAGATATCGATCACCATTTGATT |
| P*_gapA_*-*aspB*-UP-R | CGGTCGTTTTTAGGCTTCAATCATCTCAAGCCACTACTCTAGACGTTT |
| P*_gapA_*-*aspB*-DW-F | TTCCTACAATCTTTAGAGGAGACACAACATGCGGAGGTACGCAGTTATGAGT |
| *aspB*-DW-R | AGCTCGGTACCCGGGGATCCTCTAGCTTACGCATCACCGCGCGCACT |
| P*_sod_*-*aspB*-DW-F | GCGGAAACCTACGAAAGGATTTTTTACCCATGCGGAGGTACGCAGTTATGAGT |
| P*_sod_*-*aspB*-UP-R | CAGCGAATTTGGATTGGCCTTGGTTGTTTCTCTCAAGCCACTACTCTAGACGTTT |
| P*_trc_*-*aspB*-DW-F | ATTAATCATCCGGCTCGTATAATGCTTAGCGAAAGGATGGGCATGCGGAGGTACGCAGTTATGAGT |
| P*_trc_*-*aspB*-UP-R | CTTTCGCTAAGCATTATACGAGCCGGATGATTAATTGTCAACTCAAGCCACTACTCTAGACGTTT |
| *gltA*-Test-F | CGGTGACGATCTCTGGCTCGT |
| *gltA*-Test-R | CGAGGTGCTCGAGGATCTCGT |
| *gltA*-UP-F | CTTGCATGCCTGCAGGTCGACTAGGAGCAAGCTTAGAAGCCTTTGC |
| P*_gapA_*-*gltA*-UP-R | CGGTCGTTTTTAGGCTTCAATCATAAGCGCATGTCAGTTTTTGTTACTCG |
| P*_gapA_*-*gltA*-DW-F | TTCCTACAATCTTTAGAGGAGACACAACATGTTTGAAAGGGATATCGTGGCTACT |
| *gltA*-DW-R | AGCTCGGTACCCGGGGATCCTCTAGGATTGCTGCACGTGGATCGTAGT |
| P*_sod_*-*gltA*-DW-F | GCGGAAACCTACGAAAGGATTTTTTACCCATGTTTGAAAGGGATATCGTGGCTACT |
| P*_sod_*-*gltA*-UP-R | CAGCGAATTTGGATTGGCCTTGGTTGTTTCTAAGCGCATGTCAGTTTTTGTTACTCG |
| P*_trc_*-*gltA*-DW-F | ATTAATCATCCGGCTCGTATAATGCTTAGCGAAAGGATGGGCATGTTTGAAAGGGATATCGTGGCTACT |
| P*_trc_*-*gltA*-UP-R | CTTTCGCTAAGCATTATACGAGCCGGATGATTAATTGTCAAAAGCGCATGTCAGTTTTTGTTACTCG |
| *ppnK*-Test-F | ATTCACCAAAGCCTCATGATCCTCGT |
| *ppnK*-Test-R | CAATGTCTACGAGCATGCCTCAAAGTGTA |
| *ppnK*-UP-F | CTTGCATGCCTGCAGGTCGACTCCGGAAGAAATTGCGGGAAAAGTCAAT |
| P*_gapA_*-*ppnK*-UP-R | CGGTCGTTTTTAGGCTTCAATCATTTATTGCGGACCTTCCTTTACAGCC |
| P*_gapA_*-*ppnK*-DW-F | TTCCTACAATCTTTAGAGGAGACACAACATGACTGCACCCACGAACGCT |
| *ppnK*-DW-R | AGCTCGGTACCCGGGGATCCTCTAGAACAATTTATGCGAACGTTTTTTCTAAATCCT |
| P*_lysC_*-*ppnK*-DW-F | CAGCACGTAGATCGAAAGGTGCACAAAGATGACTGCACCCACGAACGCTGG |
| P*_lysC_*-*ppnK*-UP-R | GATCGCGCTCCGCACCCCAAAAGATGGTTATTGCGGACCTTCCTTTACAGCC |
| P*_ncgl0281_*-*ppnK*-DW-F | GGGATCACAAACCTTCAAAGGAGTTTGAAATGACTGCACCCACGAACGCT |
| P*_ncgl0281_*-*ppnK*-UP-R | TTTTCAGCCAGTTCACATGGAGCCACTTTATTGCGGACCTTCCTTTACAGCC |
| Ncgl0992-Test-F | TACCCCGCATTCGAACATGTCCT |
| Ncgl0992-Test-R | GGAGAACCTGCCGCATGCAAT |
| Ncgl0992-UP-F | CTTGCATGCCTGCAGGTCGACTCACCGCAAGTGGATTCTCCACCTT |
| P*_gapA_*-Ncgl0992-UP-R | CGGTCGTTTTTAGGCTTCAATCATCGGTGACGTCCATCTCGAGGGG |
| P*_gapA_*-*EcpntAB*-F | TTCCTACAATCTTTAGAGGAGACACAACATGCGAATTGGCATACCAAGAGAAC |
| *EcpntAB*-R | TCAGTCGCGGGGGACGCCAGTCGCTCGCCTCAGCAGAGGCCGTCAGGGTT |
| Ncgl0992-DW-F | AACCCTGACGGCCTCTGCTGAGGCGAGCGACTGGCGTCCCCCGCGACT |
| Ncgl0992-DW-R | AGCTCGGTACCCGGGGATCCTCTAGGATGGGCCAGGTAGACATGCGGAGTT |
| P*_lysC_*-*EcpntAB*-F | CAGCACGTAGATCGAAAGGTGCACAAAGATGCGAATTGGCATACCAAGAGAAC |
| P*_lysC_*-Ncgl0992-UP-R | GATCGCGCTCCGCACCCCAAAAGATGGCGGTGACGTCCATCTCGAGGGGG |
| P*_ncgl0281_*-*EcpntAB*-F | GGATCACAAACCTTCAAAGGAGTTTGAAATGCGAATTGGCATACCAAGAGAAC |
| P*_ncgl0281_*-Ncgl0992-UP-R | TTTTCAGCCAGTTCACATGGAGCCACTCGGTGACGTCCATCTCGAGGGG |
| Ncgl0077-Test-F | CTTCCAATTTCTTAAGAGCGAGATCACT |
| Ncgl0077-Test-R | CTGGAGGGCCTTCGCTTTAATGT |
| Ncgl0077-UP-F | CTTGCATGCCTGCAGGTCGACTTGTCATCAAAGGCCGGTGCAACG |
| Ncgl0077-UP-R | TTTCGACGTTTTCCTTCGGATCTAAACGATCTAACCAATAACCATACGGATGTAACTGAAATAC |
| *EcygaZH*-F | CCACGAAGTCCAGGAGGACATACAATGGAAAGCCCTACTCCACAGC |
| *EcygaZH*-R | GTAACGACAGTGACAAATAAAGTAAATGTATTG |
| Ncgl0077-DW-F | CAATACATTTACTTTATTTGTCACTGTCGTTACATCTATTCACATCCCTTAACCCTCACG |
| Ncgl0077-DW-R | AGCTCGGTACCCGGGGATCCTCTAGTGATGTGGAAGCCTTGGGTCTTGC |
| Ncgl0092-Test-F | AAACCATGTTGGGTCTTCTCACCTT |
| Ncgl0092-Test-R | CACCTCCGGTGAGTACCACCAT |
| Ncgl0092-UP-F | CTTGCATGCCTGCAGGTCGACTGTGGTCGCATCTCGATCGGTG |
| Ncgl0092-UP-R | TTTCGACGTTTTCCTTCGGATCTAAACGATCTAGCCGGATCGCGGTCATGCAT |
| Ncgl0092-DW-F | CAATACATTTACTTTATTTGTCACTGTCGTTACTCAACACCAAGCTGAACTGAAACTGCT |
| Ncgl0092-DW-R | AGCTCGGTACCCGGGGATCCTCTAGAAGCACACCGCCTGCGAGCAAACTT |
| *EC*-P441A-F | CTCCTTCGAATTCGCAGAATCTCCAGGC |
| *EC*-P441A**-**R | GCCTGGAGATTCTGCGAATTCGAAGGAG |
| *EC*TD-E442A-F | CTTCGAATTCCCAGCATCTCCAGGCGCTC |
| *EC*TD-E442A**-**R | GAGCGCCTGGAGATGCTGGGAATTCGAAG |
| *EC*TD-Y465A-F | CTCCCTGTTCCACGCTCGCTCCCACGGC |
| *EC*TD-Y465A-R | GCCGTGGGAGCGAGCGTGGAACAGGGAG |
| *EC*TD-R466A-F | CCTGTTCCACTATGCCTCCCACGGCACCG |
| *EC*TD-R466A**-**R | CGGTGCCGTGGGAGGCATAGTGGAACAGG |
| *EC*TD-H468A-F | CCACTATCGCTCCGCCGGCACCGATTACG |
| *EC*TD-H468A**-**R | CGTAATCGGTGCCGGCGGAGCGATAGTGG |
| *EC*TD-E347A-F | CAGTGACCATTCCAGCAGAAAAGGGCTCC |
| *EC*TD-E347A-R | GGAGCCCTTTTCTGCTGGAATGGTCACTG |
| *EC*TD-F352A-F | GAAAAGGGCTCCGCCCTGAAGTTCTGCC |
| *EC*TD-F352A-R | GGCAGAACTTCAGGGCGGAGCCCTTTTC |
| *EC*TD-R370A-F | CGAATTCAACTACGCCTTCGCAGATGC |
| *EC*TD-R370A-R | GCATCTGCGAAGGCGTAGTTGAATTCG |
| *EC*TD-N459A-F | GGGCACCTACTGGGCCATCTCCCTGTTCCAC |
| *EC*TD-N459A-R | GTGGAACAGGGAGATGGCCCAGTAGGTGCCC |
| *EC*TD-S461A-F | CTACTGGAACATCGCCCTGTTCCACTATC |
| *EC*TD-S461A-R | GATAGTGGAACAGGGCGATGTTCCAGTAG |
| *EC*TD-P441L-F | CTCCTTCGAATTCCTGGAATCTCCAGGCG |
| *EC*TD-P441L-R | CGCCTGGAGATTCCAGGAATTCGAAGGAG |
| *EC*-P441G-F | CTCCTTCGAATTCGGAGAATCTCCAGGC |
| *EC*-P441G**-**R | GCCTGGAGATTCTCCGAATTCGAAGGAG |
| *EC*-P441V-F | CTCCTTCGAATTCGTAGAATCTCCAGGC |
| *EC*-P441V**-**R | GCCTGGAGATTCTACGAATTCGAAGGAG |
| *EC*-P441I-F | CTCCTTCGAATTCATAGAATCTCCAGGC |
| *EC*-P441I**-**R | GCCTGGAGATTCTATGAATTCGAAGGAG |
| *EC*-P441M-F | CTCCTTCGAATTCATGGAATCTCCAGGC |
| *EC*-P441M**-**R | GCCTGGAGATTCCATGAATTCGAAGGAG |
| *EC*-P441W-F | CTCCTTCGAATTCTGGGAATCTCCAGGC |
| *EC*-P441W**-**R | GCCTGGAGATTCCCAGAATTCGAAGGAG |
| *EC*-P441F-F | CTCCTTCGAATTCTTTGAATCTCCAGGC |
| *EC*-P441F**-**R | GCCTGGAGATTCAAAGAATTCGAAGGAG |
| *EC*-P441S-F | CTCCTTCGAATTCTCAGAATCTCCAGGC |
| *EC*-P441S-R | GCCTGGAGATTCTGAGAATTCGAAGGAG |
| *EC*-P441T-F | CTCCTTCGAATTCACAGAATCTCCAGGC |
| *EC*-P441T-R | GCCTGGAGATTCTGTGAATTCGAAGGAG |
| *EC*-P441C-F | CTCCTTCGAATTCTGTGAATCTCCAGGC |
| *EC*-P441C-R | GCCTGGAGATTCACAGAATTCGAAGGAG |
| *EC*-P441Y-F | CTCCTTCGAATTCTATGAATCTCCAGGC |
| *EC*-P441Y-R | GCCTGGAGATTCATAGAATTCGAAGGAG |
| *EC*-P441N-F | CTCCTTCGAATTCAACGAATCTCCAGGC |
| *EC*-P441N-R | GCCTGGAGATTCGTTGAATTCGAAGGAG |
| *EC*-P441Q-F | CTCCTTCGAATTCCAAGAATCTCCAGGC |
| *EC*-P441Q-R | GCCTGGAGATTCTTGGAATTCGAAGGAG |
| *EC*-P441D-F | CTCCTTCGAATTCGATGAATCTCCAGGC |
| *EC*-P441D-R | GCCTGGAGATTCATCGAATTCGAAGGAG |
| *EC*-P441E-F | CTCCTTCGAATTCGAAGAATCTCCAGGC |
| *EC*-P441E-F | GCCTGGAGATTCTTCGAATTCGAAGGAG |
| *EC*-P441K-F | CTCCTTCGAATTCAAAGAATCTCCAGGC |
| *EC*-P441K-R | GCCTGGAGATTCTTTGAATTCGAAGGAG |
| *EC*-P441R-F | CTCCTTCGAATTCCGAGAATCTCCAGGC |
| *EC*-P441R-R | GCCTGGAGATTCTCGGAATTCGAAGGAG |
| *EC*-P441H-F | CTCCTTCGAATTCCACGAATCTCCAGGC |
| *EC*-P441H-R | GCCTGGAGATTCGTGGAATTCGAAGGAG |
| *PA*H-N11A-F | CCTGCTGCTGGAAGCCGAGCCAGGCGCATTG |
| *PA*H-N11A-R | CAATGCGCCTGGCTCGGCTTCCAGCAGCAGG |
| *PA*H-N11L-F | CCTGCTGCTGGAACTCGAGCCAGGCGCATTG |
| *PA*H-N11L-R | CAATGCGCCTGGCTCGAGTTCCAGCAGCAGG |
| *PA*H-N11F-F | CCTGCTGCTGGAATTCGAGCCAGGCGCATTG |
| *PA*H-N11F-R | CAATGCGCCTGGCTCGAATTCCAGCAGCAGG |
| *PA*H P13A-F | CTGGAAAACGAGGCAGGCGCAT |
| *PA*H P13A-R | GGACAATGCGCCTGCCTCGTTTTC |
| *PA*H P13L-F | CTGGAAAACGAGCTGGGCGCAT |
| *PA*H P13L-R | GGACAATGCGCCCAGCTCGTTTTC |
| *PA*H P13F-F | CTGGAAAACGAGTTCGGCGCAT |
| *PA*H P13F-R | GGACAATGCGCCGAACTCGTTTTC |
| PAH N29A-F | CCAACGCAACTACGCCATCGAAAGCCTGAC |
| *PA*H N29A-R | GTCAGGCTTTCGATGGCGTAGTTGCGTTGG |
| *PA*H N29L-F | CCAACGCAACTACCTGATCGAAAGCCTGAC |
| *PA*H N29L-R | GTCAGGCTTTCGATCAGGTAGTTGCGTTGG |
| *PA*H N29F-F | CCAACGCAACTACTTCATCGAAAGCCTGAC |
| *PA*H N29F-R | GTCAGGCTTTCGATGAAGTAGTTGCGTTGG |
| *PA*H P37A-F | CCTGACCGTGGCGGCGACCGAGGACCCG |
| *PA*H P37A-R | CGGGTCCTCGGTCGCCGCCACGGTCAGG |
| *PA*H P37L-F | CCTGACCGTGGCGCTGACCGAGGACCCG |
| *PA*H P37L-R | CGGGTCCTCGGTCAGCGCCACGGTCAGG |
| *PA*H P37F-F | CCTGACCGTGGCGTTCACCGAGGACCCG |
| *PA*H P37F-R | CGGGTCCTCGGTGAACGCCACGGTCAGG |
| *PA*H P41A-F | GCCGACCGAGGACGCGACCCTGTCGCGTC |
| *PA*H P41A-R | GACGCGACAGGGTCGCGTCCTCGGTCGGC |
| *PA*H-P41L-F | GCCGACCGAGGACCTGACCCTGTCGCGTC |
| *PA*H-P41L-R | GACGCGACAGGGTCAGGTCCTCGGTCGGC |
| *PA*H P41F-F | GCCGACCGAGGACTTCACCCTGTCGCGTC |
| *PA*H P41F-R | GACGCGACAGGGTGAAGTCCTCGGTCGGC |

**Table S9** The compositions of the Seed solid medium

| **Compositions** | **Concentration, g/L** |
| --- | --- |
| Sucrose | 1.5 |
| NaCl | 5 |
| Yeast extract | 7.5 |
| Yeast extract Powder | 10 |
| Tryptone | 2.5 |
| Urea | 18 |

Sodium hydroxide was used to adjust the pH to 7.0-7.2

**Table S10** The compositions of the shaking flask medium

| **Compositions** | **Concentration, g/L** |
| --- | --- |
| Glucose | 20 |
| MgSO_4_·7H_2_O | 0.5 |
| [(NH_4_)_2_SO_4_](https://baike.baidu.com/item/%E7%A1%AB%E9%85%B8%E9%93%B5/7564893) | 5 |
| KH₂PO₄ | 4 |
| K_2_HPO₄·3H_2_O | 10.5 |
| Yeast extract powder | 5 |
| Peptone | 10 |
| Urea | 3.5 |
| Leucine | 0.1 |
| Vitamin B5 | 0.02 |
| Vitamin B1 | 0.02 |
| Vitamin B3 | 0.01 |
| Biotin | 0. 055 |
| FeSO_4_·7H_2_O | 0.027 |
| MnSO_4_·H_2_O | 0.017 |
| ZnSO_4_·7H_2_O | 0.038 |
| CuSO_4_·5H_2_O | 0.6 |

Sodium hydroxide was used to adjust the pH to 7.0-7.2

**Table S11** The compositions of the fermentation tank medium

| **Compositions** | **Concentration, g/L** |
| --- | --- |
| Glucose | 75 |
| Cane molasses | 37.5 |
| Corn starch powder | 1.25 |
| Yeast extract powder | 0.5 |
| KH₂PO₄ | 0.9375 |
| Leucine | 0.1 |
| MgSO_4_·7H_2_O | 0.625 |
| MnSO_4_·H_2_O | 0. 25 |
| FeSO_4_·7H_2_O | 0.45 |
| CuSO_4_·5H_2_O | 0.1 |
| ZnSO_4_·7H_2_O | 0.015 |
| Vitamin B5 | 0.025 |
| Vitamin B1 | 0.025 |
| Vitamin B3 | 0.0125 |
| Biotin | 0.1 |
| pH | 7.0-7.2 |

Sodium hydroxide was used to adjust the pH to 7.0-7.2

**Table S12** The compositions of LBGB

| **Compositions** | **Concentration, g/L** |
| --- | --- |
| Yeast extract | 5 |
| NaCl | 10 |
| Peptone | 10 |
| Glucose | 5 |
| Brain heart infusion | 18.5 |

**Table 13** The compositions of the BT medium

| **Compositions** | **Concentration, g/L** |
| --- | --- |
| Glucose | 40 |
| (NH2)_2_CO | 2 |
| (NH_4_)_2_SO_4_ | 7 |
| K_2_HPO_4_ | 0.5 |
| KH_2_PO_4_ | 0.5 |
| MgSO_4_·7H_2_O | 0.5 |
| FeSO_4_·7H_2_O | 0.006 |
| MnSO_4_·H_2_O | 0.0042 |
| Biotin | 0.0002 |
| Thiamine | 0.0002 |

NH_3_·H_2_O was used to adjust the pH to 7.6

BT medium used for evaluation of the dynamic response range of the isoleucine biosensor

**Supplementary methods**

**Method S1 Measurement of intracellular cofactors**

The intracellular content of NADPH, NADH and ATP were quantiﬁed using the Biochemical reagent kit (Beyotime Biotechnology, China). Samples were individually extracted with the extraction buffers and centrifuged and resuspended in sterile PBS buffer. All subsequent steps were performed according to the manufacturer’s instructions. In the coenzyme II (NADP^+^/NADPH) content test kit, Glucose-6-phosphate was oxidized to 6-phosphogluconate (6-PG) by glucose-6-phosphate dehydrogenase (G6PDH), in which NADP^+^ is reduced to NADPH. The resulting NADPH was used to reduce WST-8 to orange-yellow formazan by the electron coupling reagent 1-mPMS (1-Methoxy-5-methylphenazinium Methyl Sulfate), and the maximum absorption peak was detected at about 450 nm. The amount of formazan generated in the reaction system is proportional to the total amount of NADP^+^ and NADPH in the sample. The amount of NADPH was determined separately: After 30min of heating in a 60 ºC water bath, NADP^+^ in the sample would decompose and only NADPH would be retained. NADPH reduces WST-8 to formazan, and the amount of formazan generated by the reaction is determined by colorimetry, which eventually allows the amount of NADPH in the sample to be determined. In the coenzyme I (NAD^+^/NADH) content test kit, ethanol was oxidized to acetaldehyde under the action of alcohol dehydrogenase (ADH), in which NAD^+^ was reduced to NADH. The generated NADH reduces WST-8 to orange-yellow formazan with a maximum absorption peak around 450nm in the presence of the electronically coupled reagent 1-mPMS (1-Methoxy-5-methylphenazinium Methyl Sulfate). The formazan generated in the reaction system was proportional to the total amount of NAD^+^ and NADH in the sample. The amount of NADH was determined separately: After 30min of heating in a 60 ºC water bath, NAD^+^ in the sample would decompose and only NADH would be retained. NADH reduces WST-8 to formazan, and the amount of formazan generated by the reaction is determined by colorimetry, which eventually allows the amount of NADH in the sample to be determined.

**Method S2 HPLC mobile phases and gradient program**

HPLC analysis was performed using gradient elution with UV detection at 254 nm. Mobile phase consisted of acetonitrile–methanol–water (50:30:10, v/v/v). Mobile phase B was 0.01 mol/L KH_2_PO_4_ buffer: KH_2_PO_4_ (1.361 g) was dissolved in 950 mL ultrapure water, the pH was adjusted to 5.30 with KOH, the volume was brought to 1.0 L, and the solution was subsequently filtered and sonicated.

The column temperature was maintained at 35 °C, with a flow rate of 1.0 mL/min and an injection volume of 15 μL. The total run time was 30 min The gradient program was applied as follows (A/B, v/v): 0 min, 20/80; 5 min, 35/65; 10 min, 65/35; 15 min, 70/30; 20 min, 50/50; 23 min, 20/80.
